# CRlncRC: a machine learning-based method for cancer-related long noncoding RNA identification using integrated features

**DOI:** 10.1186/s12920-018-0436-9

**Published:** 2018-12-31

**Authors:** Xuan Zhang, Jun Wang, Jing Li, Wen Chen, Changning Liu

**Affiliations:** 1 CAS Key Laboratory of Tropical Plant Resources and Sustainable Use, Xishuangbanna Tropical Botanical Garden, Chinese Academy of Sciences, Menglun, 666303 Yunnan People’s Republic of China; 2 University of Chinese Academy of Sciences, Beijing, 100049 People’s Republic of China; 3 Institute of Medical Sciences, Xiangya Hospital, Central South University, Changsha, 410008 People’s Republic of China

**Keywords:** Cancer-related, LncRNA, Classification, Integrated features, Machine learning

## Abstract

**Background:**

Long noncoding RNAs (lncRNAs) are widely involved in the initiation and development of cancer. Although some computational methods have been proposed to identify cancer-related lncRNAs, there is still a demanding to improve the prediction accuracy and efficiency. In addition, the quick-update data of cancer, as well as the discovery of new mechanism, also underlay the possibility of improvement of cancer-related lncRNA prediction algorithm. In this study, we introduced CRlncRC, a novel Cancer-Related lncRNA Classifier by integrating manifold features with five machine-learning techniques.

**Results:**

CRlncRC was built on the integration of genomic, expression, epigenetic and network, totally in four categories of features. Five learning techniques were exploited to develop the effective classification model including Random Forest (RF), Naïve bayes (NB), Support Vector Machine (SVM), Logistic Regression (LR) and K-Nearest Neighbors (KNN). Using ten-fold cross-validation, we showed that RF is the best model for classifying cancer-related lncRNAs (AUC = 0.82). The feature importance analysis indicated that epigenetic and network features play key roles in the classification. In addition, compared with other existing classifiers, CRlncRC exhibited a better performance both in sensitivity and specificity. We further applied CRlncRC to lncRNAs from the TANRIC (The Atlas of non-coding RNA in Cancer) dataset, and identified 121 cancer-related lncRNA candidates. These potential cancer-related lncRNAs showed a certain kind of cancer-related indications, and many of them could find convincing literature supports.

**Conclusions:**

Our results indicate that CRlncRC is a powerful method for identifying cancer-related lncRNAs. Machine-learning-based integration of multiple features, especially epigenetic and network features, had a great contribution to the cancer-related lncRNA prediction. RF outperforms other learning techniques on measurement of model sensitivity and specificity. In addition, using CRlncRC method, we predicted a set of cancer-related lncRNAs, all of which displayed a strong relevance to cancer as a valuable conception for the further cancer-related lncRNA function studies.

**Electronic supplementary material:**

The online version of this article (10.1186/s12920-018-0436-9) contains supplementary material, which is available to authorized users.

## Background

Cancers are multi-factor complicated diseases, and primarily triggered by genetic alteration and gene-regulatory-network disorder under various environmental irritations [1]. Increasing evidence showed that long non-coding RNAs (lncRNAs), a class of transcripts with a very low protein-coding potential and length more than 200 bp, could widely participate in the occurrence and progression of multiple cancers, with the capability to perturb the cellular homeostasis potentially by remodeling chromatin architecture or regulating the transcriptional outcomes [2–6]. Recent rapid development of next-generation sequencing has promoted the detection of thousands of expression profiles between pairs of cancer and regular transcriptomes, revealing that there were many aberrant lncRNAs emerged in the course of cancer occurrence and development [7–9]. However, for the vast majority of them, it is hard to distinguish which are functioning or what their potential roles in cancers, due to the low expression level, poor conservation, uncertain mutation mode, and diverged tissue specificity. Therefore, it is imperative to develop systematic and bioinformatics tools for further predicting and exploring the possible functions of lncRNAs in cancer.

Recently, several methods have been designed to identify potential cancer-related lncRNAs. For example, Zhao et al. developed a naïve-Bayesian-based classifier to identify cancer-related lncRNAs by integrating both genome, regulome and transcriptome data, and identified 707 potential cancer-related lncRNAs [10]. They also found that four of six mouse orthologous lncRNAs were significantly involved in many cancer-related processes, based on 147 lncRNA knockdown data in mice. Lanzós Andrés et al. conceived a tool (ExInAtor) to identify cancer driver lncRNA genes with an excess load of somatic single nucleotide variants (SNVs) and consequently found 15 high-confidence candidates: 9 novel and 6 known cancer-related lncRNA genes [11]. However, this kind of studies is still at infancy, and would be bound to a measure of limitations in the aspects of accuracy and sensitivity. For example, ExInAtor that aimed at discovering driver lncRNAs in cancer was subjected to the likelihood of losing the prediction sensitivity, as mentioned by themselves. Therefore, different algorithms of the classification model should be developed reasonably, and important features should be further explored systematically, in order to advance the sensitivity and accuracy when we are seeking the cancer-related lncRNAs.

Besides, some cancer-related features of lncRNAs are necessary for the purpose of distinction. Apparently, the ordinary differential expression analysis between pairs of cancerous and normal tissue could not favor the prediction requirements, due to the high false positive rate. Hence, other features of lncRNAs (like genomic location, tissue specialty, exon mutation frequency, somatic single nucleotide variants, co-expression relationships between lncRNAs and protein-coding genes, etc.) were integrated into the computational analysis to better discriminate the cancer-related lncRNAs from the negative ones. However, mining these features is also a gradually evolutional process. For example, Chen. et al. found broad H3K4me3a was associated with increased transcription elongation and enhancer activity of tumor suppressor genes [12], implying that some epigenetic features could be added into cancer-related lncRNAs’ identification.

Here, we developed a compounded computational method, CRlncRC, to predict cancer-related lncRNAs. CRlncRc was based on five machine learning models, including Random Forest (RF), Naïve bayes (NB), Support Vector Machine (SVM), Logistic Regression (LR) and K-Nearest Neighbors (KNN). Beyond that, CRlncRC was built on the integration of four categories of features (i.e. genomic, expression, epigenetic and network), more lncRNA’s features were introduced into our analysis to enhance the prediction sensitivity. We demonstrated that our integrative method significantly improves the accuracy of identification of cancer-related lncRNAs, as compared with some previous methods. RF model outperforms other learning models on measurements of model sensitivity and specificity. We also showed that machine learning-based integration of multiple features had a great contribution to the cancer-related lncRNA prediction, wherein epigenetic and genomic features play key roles in the classification. Next, we used CRlncRC method to predict a set of cancer-related lncRNAs from the TANRIC dataset. These novel cancer-related candidates were further evaluated by somatic mutation number in cancer genome, distance with the cancer-related proteins, differential expression fold change in tumor and normal tissues, and GO enrichment analysis. The results indicated that the predicted set have a strong cancer correlation, many of which could find convincing literature supports. We believed that these fresh cancer-related lncRNAs would be a valuable starting point for the further cancer-related lncRNA functional study.

## Results and discussion

### Overview of CRlncRC

**Fig. 1:**
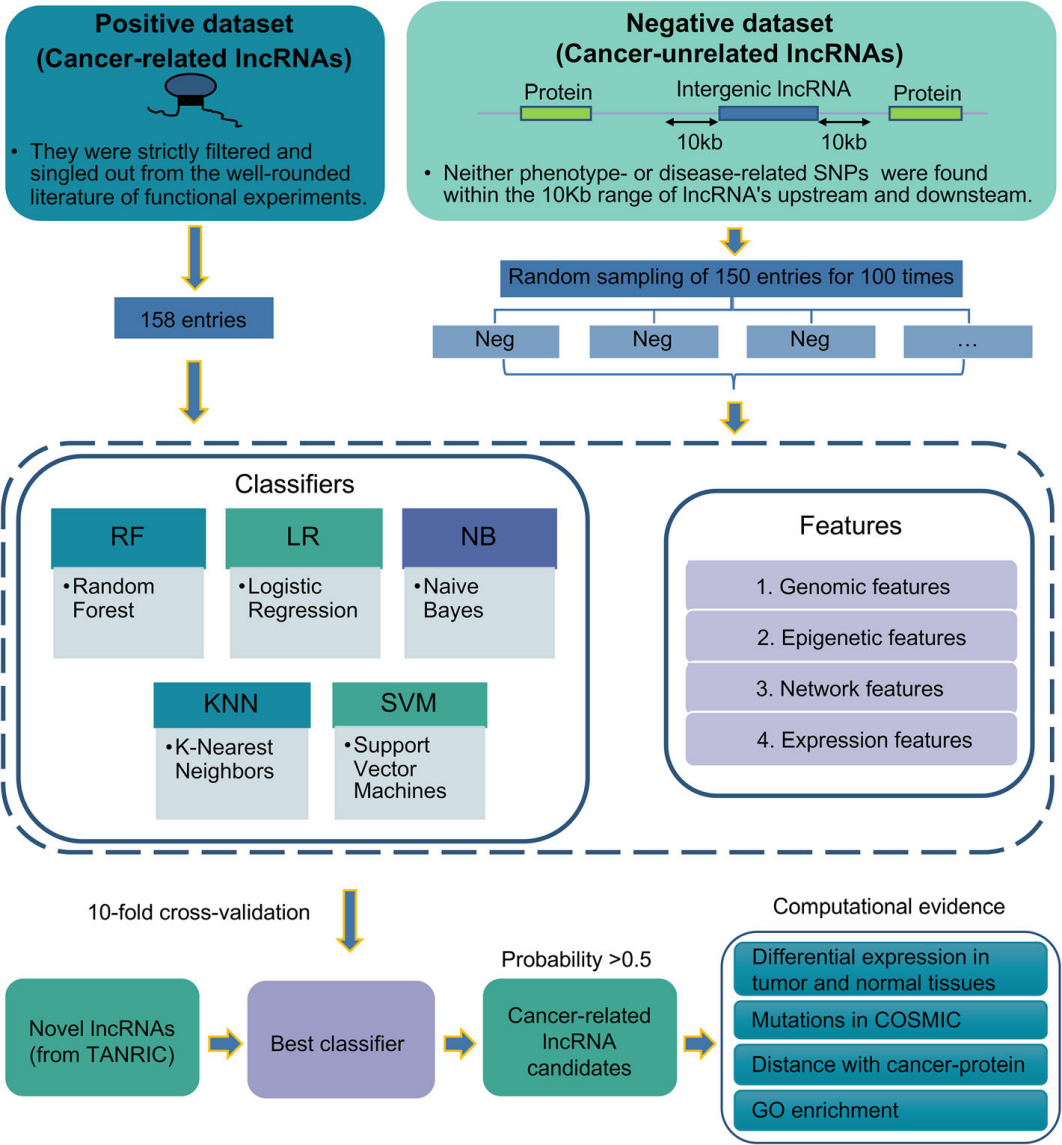
A systematic overview of the CRlncRC. A work frame of CRlncRC (cancer-related lncRNA classifier) for predicting and characterizing novel cancer-related lncRNA candidates in human

An integrated machine-learning pipeline was designed and designated as CRlncRC (Cancer-Related lncRNA Classifier). The pipeline was shown in Fig. 1.

Firstly, in order to increase the precision of predictions, we strictly selected the positive and negative collection for training. The positive dataset consisted of 158 experimentally-validated cancer-related lncRNAs curated from the scientific literature (Additional file 1); while the negative was randomly sampled from long intergenic noncoding RNAs whose 10 kb upstream and downstream had no cancer-related SNPs (in total 4553 lncRNAs, Additional file 2), repeatedly 100 times. For lncRNAs from each dataset, we constructed four categories of features including genomic, expression, epigenetic and network (Additional file 3).

Second, to evaluate the performance of different machine-learning algorithms, we used five popular algorithms, including Random Forest (RF), Naïve bayes (NB), Support Vector Machine (SVM), Logistic Regression (LR) and K-Nearest Neighbors (KNN), to proceed with ten-fold cross-validation in 100 training datasets. For each test, the receiver operating characteristic curves (ROCs) were calculated, and the average area under the ROC curve (AUC) was used to assess the best performance for each algorithm in 100 training sets. For the best performance model, we further compared it with other existing cancer-related lncRNA classifiers in terms of performance, and evaluated the weightiness of four categories of features contributing to cancer-related classification.

At last, we managed to use the best performance model to predict novel cancer-related lncRNAs. Here, we adopted all lncRNAs from the TANRIC dataset (Additional file 4), which were completely separate from our positive and negative datasets, to examine the prediction performance of CRlncRC. For these novel cancer-related candidates, we utilized genome-wide data to assess the probability of their associations with cancer, which include their enrichment of somatic mutations in cancer genome, distance with the cancer-related proteins, differential expression fold-change between pairs of tumor and normal tissues, and GO enrichment analysis. In addition, we also inspected the potent experimental supports from literature.

### Cross-validation accuracy

**Fig. 2:**
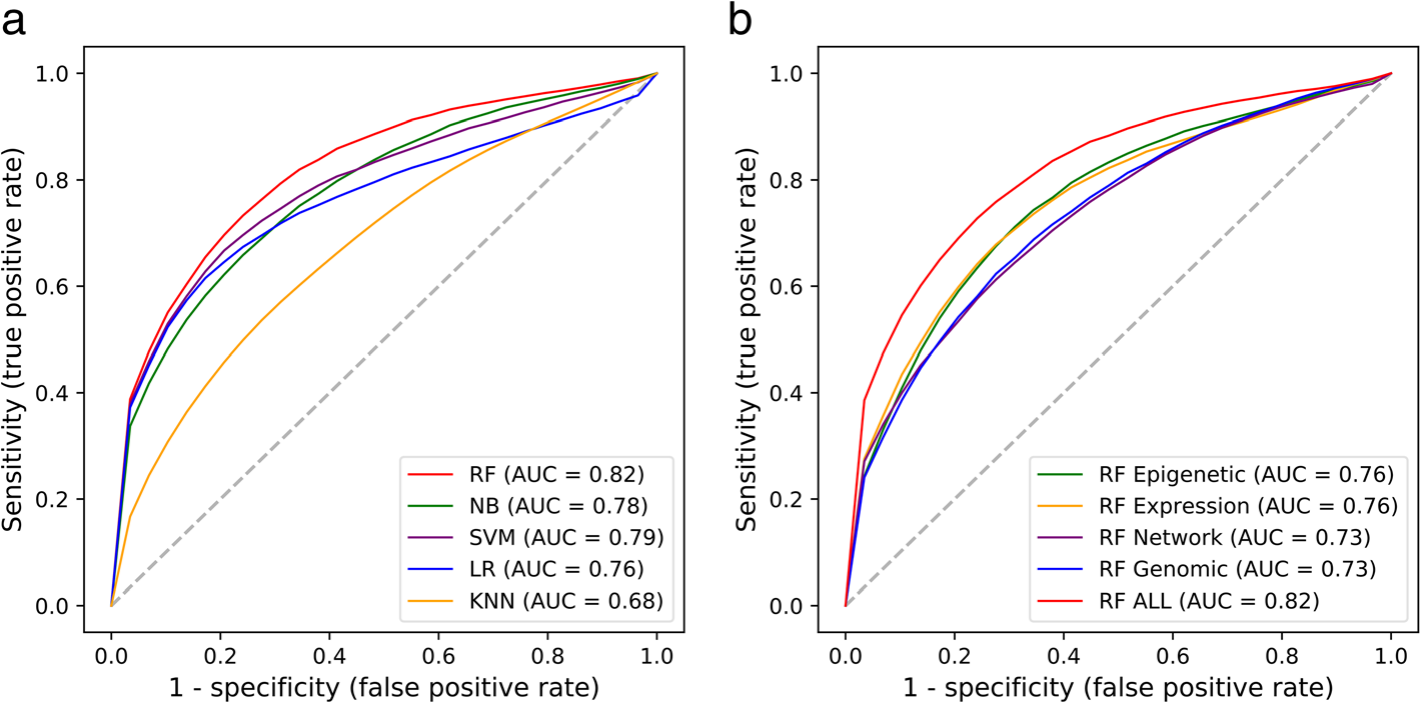
Prediction performance of ten-fold cross-validation. **a** Comparison between the performance of five machine learning methods. **b** Comparison between the performance of RF corresponding to individual types of features and all features

We used ten-fold cross-validation to evaluate the model accuracy. As shown in Fig. 2a, RF, NB, SVM, LR and KNN achieved average AUC scores of 0.82, 0.78, 0.79, 0.76 and 0.68, respectively. Apparently, there are four models achieving an average score of AUC more than 0.75 except that of KNN, wherein RF model shows the best performance. We next checked the resulting accuracy of RF classifier when we used only one category of features. As shown in Fig. 2b, training RF model with epigenetic, expression, network and genomic features, our model achieved AUC scores of 0.76, 0.73, 0.76 and 0.73, respectively. So, using RF model, any feature solely could gain an average AUC score of > 0.7, much less an extra 6–9% AUC score when combining all the features. No single category of feature could achieve the top performance as that of united features, which strongly suggests the complementary nature between features and the advantage of integrative approaches. In addition to AUC value, more evaluation indicators were used to assess our results such as precision, recall, accuracy, and AUC confidence interval (Additional file 5). In order to perform a comprehensive analysis of the effect of features on model’s performance, we also compared two types of features and three types of features (Additional file 6).

### The contribution of features to identify cancer-related lncRNAs

**Fig. 3:**
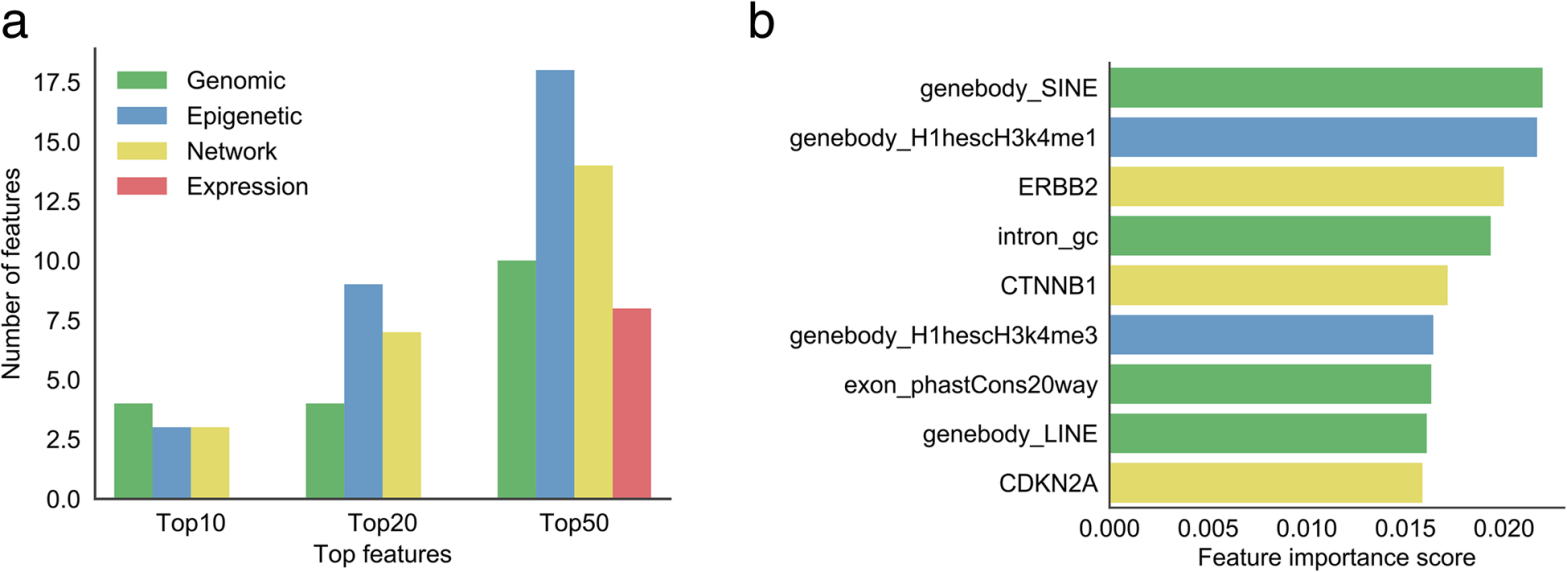
Feature importance. **a** The distribution of four feature categories (genomic, epigenetic, network, and expression) in top 10, top 20, and top 50 features. **b** The rank of top 9 features (genomic, epigenetic, network, expression features colored in green, blue, yellow, and red, respectively)

To better comprehensively understand the significance of features to identification of the cancer-related lncRNAs, we used ExtraTreeClassifier [13] in scikit-learn package as a measurement for further evaluating the hierarchies of all features in terms of importance (Fig. 3) (Additional file 3). Here, we summarized the amount of four categories of features located in Top10/20/50 feature importance list (Fig. 3a). In the Top10 features, four features are pertinent to genomic features, epigenetic and network features each have three positions. However, as respects of the Top20 and Top50 features, the first and the second occupancy among features belong to epigenetic (9 in Top20 and 18 in Top50) and network features (7 in Top20 and 14 in Top50), respectively. It surprised us that no expression features emerged in the Top10 and Top20 features, which only occupy 8 locations in the Top50 features, indicating that expression features are still necessary though less important than other types of features. The fact that lncRNA expression always had a strong tissue specificity with a relatively low level might explain the less importance of expression-related features on cancer-related lncRNAs prediction.

**Fig. 4:**
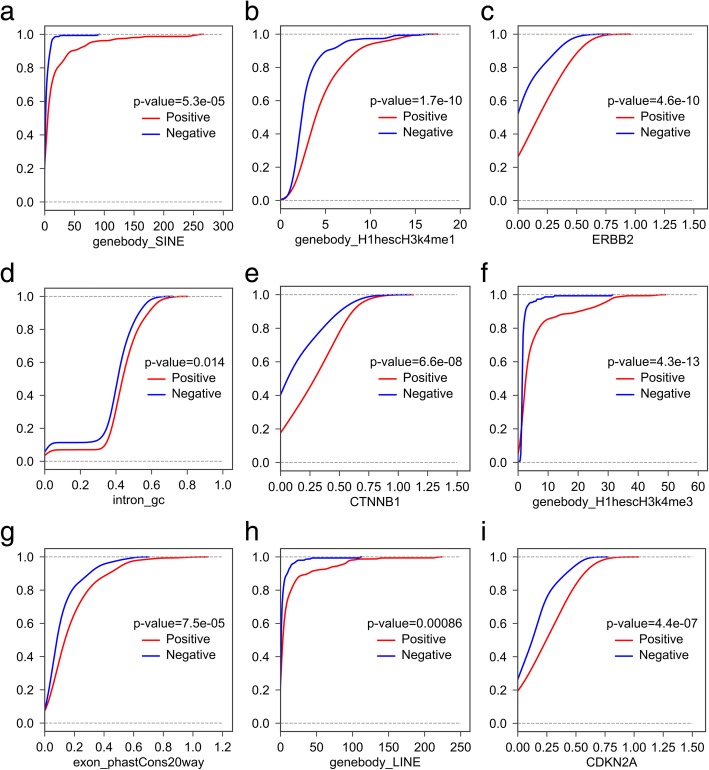
Cumulative percentage comparisons (Kolmogorov-Smirnov test) of the top 9 features between the positive and negative lncRNAs. **a** SINE number in gene body. **b** Average H1hescH3K4me1 signal in gene body. **c** Spearman’s correlation coefficient with ERBB2. **d** GC content of intron. **e** Spearman’s correlation coefficient with CTNNB1. **f** Average H1hescH3K4me3 signal in gene body. **g** Conservation level computed using PhastCons applied to the 20-way whole-genome in the exon. **h** LINE number in gene body. **i** Spearman’s correlation coefficient with CDKN2A

We calculated the cumulative distribution and corresponding Kolmogorov-Smirnov test *p*-value of all the features in the positive and negative lncRNA datasets (Additional file 7). Figure 3b and Fig. 4 showed the top nine features sorted by importance, and their corresponding cumulative distribution in the positive and negative datasets. Interestingly, ‘SINE (Short interspersed nuclear element) numbers in gene body’ was the most important feature. The cumulative curve also showed that cancer-related lncRNAs have obviously higher SINE numbers than cancer-unrelated lncRNAs (Fig. 4a, *p*-value = 5.3e-05). LINE (Long interspersed nuclear element) was another example of the repeats which contributes to the classifier. ‘LINE numbers in gene body’ ranks the No.8 in all of the features. Similar with SINE, we found that cancer-related lncRNAs have obviously higher LINE numbers than cancer-unrelated lncRNAs (Fig. 4h, *p*-value = 0.00086). We further compared the length distribution of positive and negative lncRNAs, and found that these two distributions only have slight difference (Additional file 8). Our results implied that repeat element might be an important functional element for lncRNAs and widely participant in the cancer-related process, which is consistent with a lot of published researches. For example, Alu is a subtype of SINE and has been implicated in several inherited human diseases and in various forms of cancers; and, LINE can activate immune responses and contribute to disease progression [14, 15], as well as potentially affect chromatin formation [16].

Apart from the repeat, there are other two genomic-related features in the top nine features: ‘intron GC content’ and ‘exon phastCons score’. Compared with lncRNAs from negative dataset, the introns in cancer-related lncRNAs have a relatively higher GC content (Fig. 4d, *p*-value = 0.014). The GC content was related to the stability of gene and regulation and might have played a significant role in the evolution [17]. Besides, the composite patterns of GC content between intron and exon would likely affect gene splicing [18, 19]. These facts hinted the relationship between ‘intron GC content’ and cancer-related lncRNA. Moreover, the exon sequences in cancer-related lncRNAs showed obviously higher conservation than negative set, implying that cancer-related lncRNAs may undergo evolutionary pressure for maintaining some important functions relevant to normal cell behavior (Fig. 4g, *p*-value = 7.5e-05).

In the top nine features, two epigenetic features ranked at NO.2 and No.6, they are “H3k4me1” and “H3k4me3” epigenetic modification signals within lncRNA gene body region in H1hesc cell line, respectively. Both signals in positive dataset are significantly higher than the negative set (Fig. 4b, *p*-value = 1.7e-10; Fig. 4f, *p*-value = 4.3e-13). Epigenetic feature H3k4me3 are likely associated with the expression of cancer-related lncRNAs. High levels of H3K4me3 are often found near the promoter region, and commonly associated with the activation of transcription of nearby genes [20, 21]. A broad H3K4me3 is associated with increased transcription elongation and enhancer activity at tumor-suppressor genes [12]. While H3K4me1 is usually found in intergenic region with enrichment at enhancers [22]. Recent studies have demonstrated that many enhancer elements can be transcribed into a novel class of lncRNAs, enhancer RNAs (eRNAs) [23–25]. These eRNAs could exert cancer-related functions through their associated enhancers, as in the case of eRNAs from p53-bound enhancer region that are required for p53-dependent enhancer activity and gene transcription [26]. On the other hand, the fact that these two histone modification related features listed in top 9 features are associated with H1hesc cell line, instead of Gm12878 and K562 cell lines, indicated that the effects of histone modifications to cancer-related lncRNAs might have tissue/cell type-specificity.

Consistent with the other papers, the cancer-related lncRNAs tend to be more likely interacted with cancer-related proteins. In the top nine features, network features ranked the position of No.3, No.5 and No.9. The lncRNAs in the positive set displayed more strongly co-expression with ERBB2, CTNNB1 and CDKN2A than the negative set (Fig. 4c, *p*-value = 4.6e-10; Fig. 4e, *p*-value = 6.6e-08; Fig. 4i, *p*-value = 4.4e-07). Wherein, ERBB2 was found associated with Glioma Susceptibility 1 and Lung Cancer; CTNNB1 is part of a complex of proteins that constitute adherens junctions, mutations in CTNNB1 are a cause of colorectal cancer, pilomatrixoma, medulloblastoma, and ovarian cancer; while CDKN2A (i.e. p16) is frequently mutated or deleted in a wide variety of tumors and is known to be an important tumor suppressor gene.

### Comparison with other cancer-related lncRNA prediction algorithms

We used ten-fold cross-validation to compare the prediction performance of our CRlncRC with that of the other two developed prediction algorithms as mentioned in Background. Considering that the latter two developed early and were comprised of relatively small-scale datasets (for example, Zhao et al. collected 70 cancer-related lncRNAs as positive dataset, while Lanzós Andrés et al. collected 45 cancer-related lncRNAs as positive dataset), for fairness, we applied their datasets for retraining our RF model rather than our own well-established model.

**Fig. 5:**
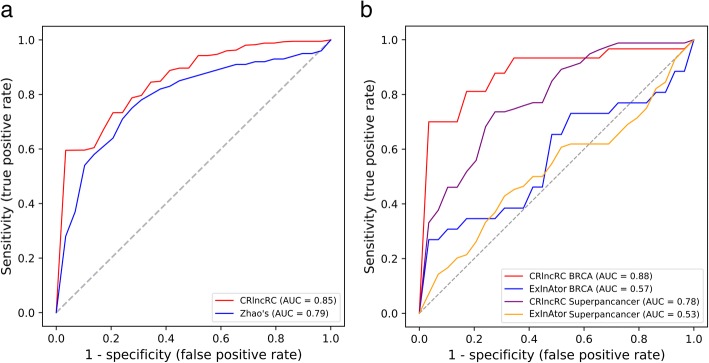
Compare with other methods. **a** Comparison between the performance of CRlncRC and Zhao’s method. **b** Comparison between the performance of CRlncRC and ExInAtor in BRCA and Superpancancer

As shown in Fig. 5a, the AUC score of our method reached 0.85, much higher than 0.79 reported in Zhao’s results. In the aspects of the feature choice, we adopted genomic, network, expression, and epigenetic (totally four categories of features) in our model, while Zhao et al. selected three features of genome, regulome and transcriptome in their prediction model. This result suggested that the newly introduced epigenetic features in CRlncRC, which not include in Zhao’s study, may have a great contribution to the classification between cancer-related lncRNAs and cancer-unrelated lncRNAs. On the other hand, compared with the NB model used in Zhao’s method, CRlncRC employed RF as its learning model after broad evaluation of five learning techniques, with a dominant consequence of performance enhancements.

A cancer driver gene is defined as one whose mutations increase net cell growth under the specific micro-environmental conditions that exist in the cell in vivo [27]. While a cancer-related lncRNA can be defined as it can promote or inhibits the growth of cancer cells through some mechanism [28]. To comprehensively discover the candidates of cancer driver lncRNAs, Lanzós Andrés et al. developed ExInAtor and run it on 23 tumor types. We choose ‘BRCA’ that is believed as the best tumor type of prediction in Lanzós Andrés’s work and ‘Superpancancer’ to do the comparison, two of which respectively represent the type-specific and the ubiquitous cancer-related lncRNA gene discovery. As shown in Fig. 5b, our model had an obvious superiority against ExInAtor in both ‘Superpancancer’ (AUC score 0.78 vs. 0.53) and ‘BRCA’ (AUC score 0.88 vs. 0.57). These results suggested that ExInAtor is probably just perfect for finding cancer driver lncRNAs when considering of only one feature of genomic somatic mutation, but do not suit for the prediction of cancer-related lncRNAs.

### Systematic evaluation of predicted novel cancer-related lncRNAs

We used CRlncRC to predict novel cancer-related lncRNAs from TANRIC lncRNA dataset (Additional file 4), which was completely separate from our positive and negative datasets. The 11,656 unknown lncRNAs were assessed by use of the best RF model we trained. In total, 121 cancer-related lncRNA candidates were identified (Additional file 9), including 55 antisense lncRNAs, 57 intergenic lncRNAs and 9 overlapping lncRNAs. For these novel cancer-related candidates, we further utilized genome-wide data to systematically evaluate the probability of their associations with cancer. For that purpose, three types of lncRNA set were applied to our analysis, including cancer-related lncRNAs (positive), cancer-unrelated lncRNAs (negative), and predicted novel cancer-related lncRNAs (predict).

**Fig. 6:**
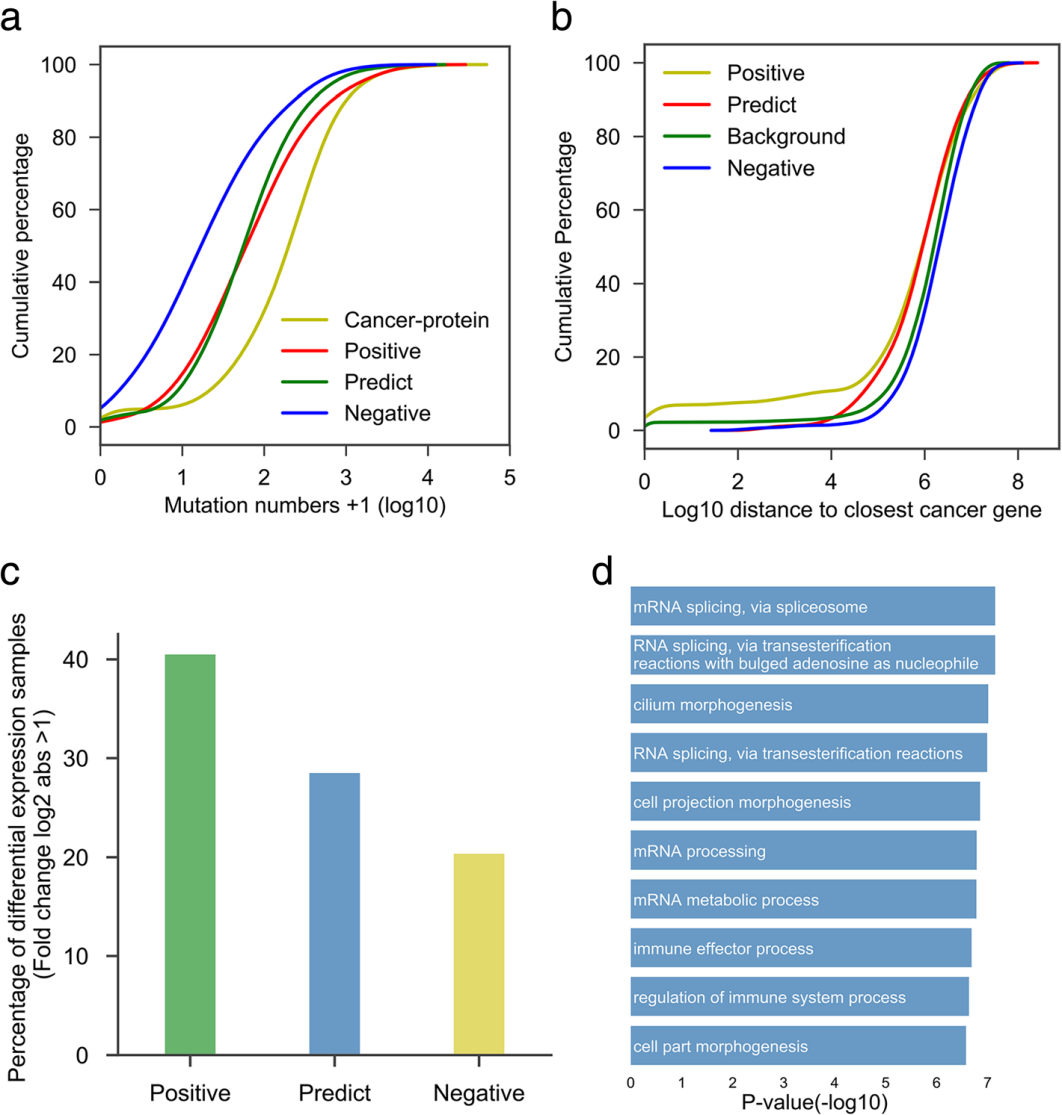
Validation of our novel cancer-related lncRNAs candidates. **a** Cumulative distribution of mutation number. **b** Cumulative distribution of the closest distance to cancer-related proteins. **c** Bar plot of the percentage of differential expression lncRNAs. **d** The Top 10 GO BP terms of cancer-related lncRNA candidates (Fisher’s exact test)

First, we assumed that these potential cancer-related lncRNAs were likely to have more somatic mutations in cancer genomes, since many previous studies had demonstrated that mutation in function genes is a main cause of cancer induction. To validate the assumption, we made a comparison of the number of somatic mutations (documented in COSMIC) between different lncRNA sets and cancer-related protein set (Fig. 6a). As a result, cancer-related protein set as the positive control possessed far more somatic mutations than cancer-unrelated lncRNA set, which is the negative control (Kolmogorov-Smirnov test, *p*-value = 6.10e-33). The somatic mutation numbers in both positive and predicted cancer-related lncRNA sets are between cancer-unrelated lncRNAs and cancer-related proteins, with a significant higher quantities than that of cancer-unrelated lncRNAs (Kolmogorov-Smirnov test, *p*-value 2.35e-07 and 3.25e-06 respectively).

Because a number of lncRNAs exert their function *in cis* to influence their neighboring genes, we assumed that these potential cancer-related lncRNAs likely have a closer distance with cancer-related proteins by comparison of cancer-unrelated lncRNAs. Therefore, we calculated the distance of different lncRNA sets to their closest cancer-related proteins, and compared it with the random background (that is the distance between cancer-related proteins and random positions in genome) (Fig. 6b). We found that the distances between cancer-unrelated lncRNAs and cancer-related proteins are significantly larger than that between cancer-related lncRNAs and cancer-related proteins (Kolmogorov-Smirnov test, *p*-value = 0.00041). Similarly, the distance of predicted cancer-related lncRNAs to cancer-related proteins is far closer than to cancer-unrelated lncRNAs (Kolmogorov-Smirnov test, *p*-value = 0.00116). Moreover, no significant difference was detected between background and cancer-unrelated lncRNAs set, as expected.

Next, we examined whether it is possible that the expression levels of cancer-related lncRNAs in cancer have a more marked change as compared with that of cancer-unrelated lncRNAs (Fig. 6c). By using lncRNA expression data from TANRIC database, we calculated the percentage of lncRNA differential expressed between pairs of cancer and paracancerous tissues (lncRNAs with absolute log2-fold change greater than 1), to see if there is a difference among different lncRNA sets. We found that lncRNAs in positive set had the highest percentage of differential expressed genes (about 40%), while negative set only with about 20%. For those predicted cancer-related lncRNAs, over 28% of them showed differential expression. This result further supported our prediction products have an evident association with cancer, and also revealed that simple dependence on differential expression is far from enough for identification of cancer-related lncRNAs.

Finally, we investigated the GO (Gene Ontology) annotations of these cancer-related lncRNAs candidates. LncRNA’s GO annotations were predicted according to the enriched GO terms of its neighboring proteins in the co-expression network. The Top10 (sorted by *p*-value, Fisher’s exact test) enriched GO terms were listed in Fig. 6d. From the list, we can found that the functions of these cancer-related lncRNA candidates mainly focused on the following keywords: 1) ‘RNA splicing’, such as ‘mRNA splicing, via spliceosome’, ‘RNA splicing, via transesterification reactions with bulged adenosine as nucleophile’, and ‘RNA splicing, via transesterification reactions’; 2) ‘morphogenesis’, such as ‘cilium morphogenesis’, ‘cell projection morphogenesis’, and ‘cell part morphogenesis’; 3) ‘immune’, such as ‘immune effector process’ and ‘regulation of immune system process’; 4) ‘mRNA processing ‘, such as ‘mRNA processing’ and ‘mRNA metabolic process’. These annotations revealed the potential action modes of cancer-related lncRNAs, which is consistent with many of the latest studies. For example, Simon et al. discovered that a bifunctional RNA, encoding both PNUTS mRNA and lncRNA-PNUTS, could mediate EMT and tumor progression when its splice switches from coding to noncoding transcript [29]. Musahl et al. found ncRNA-RB1 could positively regulate the expression of calreticulin (CALR) and sequentially activate anticancer immune responses [30].

### Case study of the cancer-related lncRNA candidates

Besides utilizing genome-wide data to systematically evaluate these cancer-related lncRNA candidates, we also drilled down into some lncRNA cases. To our amazement, in the Top10 cancer-related lncRNA candidates in our prediction results, there are six predicted lncRNAs (NNT-AS1, TP53TG1, LINC01278, LRRC75A-AS1, MAGI2-AS3, EIF3J-AS1) to be found with literature supports. For example, lncRNA NNT-AS1 could promote cell proliferation and invasion through Wnt/β-catenin signaling pathway in cervical cancer [31] and contribute to proliferation and migration of colorectal cancer cells both in vitro and in vivo [32]. Besides, it can promote hepatocellular carcinoma and breast cancer progression through targeting miR-363/CDK6 axis [33] and miR-142-3p/ZEB1 axis [34], respectively. Another example is a p53-induced lncRNA TP53TG1, which is a newly identified tumor-suppressor gene and plays a distinct role in the p53 response to DNA damage. TP53TG1 hypermethylation in primary tumors is shown to be associated with poor outcome [35]. According the newest research findings, TP53TG1 participated in the stress response under glucose deprivation in glioma [36], and enhanced cisplatin sensitivity of non-small cell lung cancer cells through regulating miR-18a/PTEN axis [37].

Besides the lncRNAs mentioned above, another very interesting lncRNA -- UBR5-AS1 (UBR5 antisense RNA1) -- came into our view. UBR5-AS1 sits between two protein-coding genes (UBR5 and P53R2). The 3′ terminal sequence of UBR5 is partial antisense to UBR5, the latter is an oncogene in many cancers and contributes to cancer progression, cell proliferation [38, 39]. The 5′ end of UBR5-AS1 is positioned head-to-head (or divergent) to P53R2, which is believed to play essential roles in DNA repair, mtDNA synthesis and protection against oxidative stress, and has a positive correlation with drug sensitivity and tumor invasiveness [40]. Since a host of studies had demonstrated that lncRNAs often exert their function *in cis* to influence their neighboring genes, we have good reasons to believe that UBR5-AS1 is very likely to be associated with cancer. However, till now UBR5-AS1 has not been studied by researchers.

**Fig. 7:**
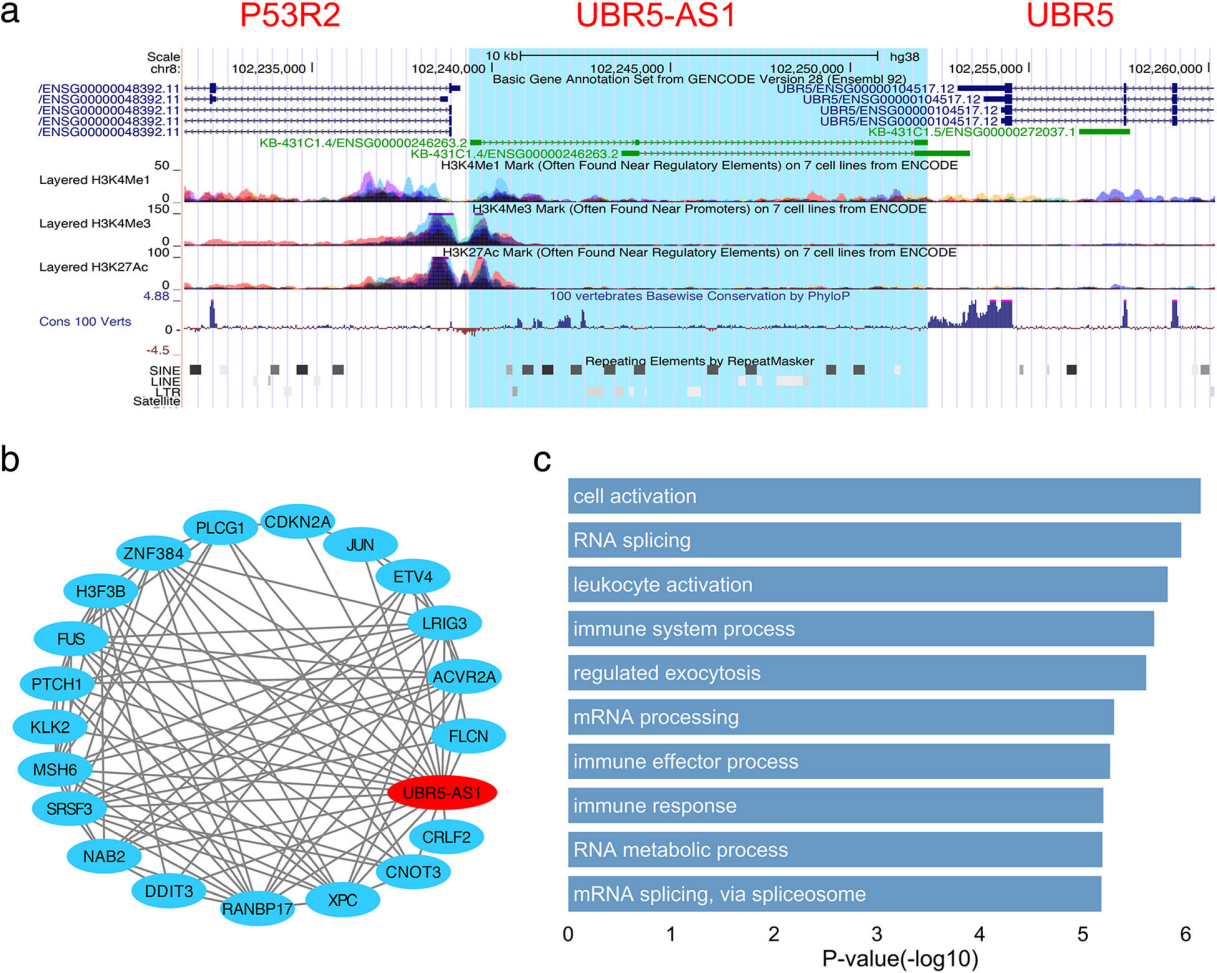
Characterization of lncRNA UBR5-AS1. **a** The gene structure, epigenetic, conservation and repeat features of UBR5-AS1 in UCSC genome browser. **b** The co-expression sub-network of UBR5-AS1. **c** The Top 10 GO BP annotations of UBR5-AS1 (Fisher’s exact test)

Figure 7a showed UBR5-AS1 and its neighbor region, with a variety of information about epigenetics, conservation and repeats (as visualized by UCSC genome browser). We can see that the shared promoter region between UBR5-AS1 and P53R2 had high H3K4me3 and H3K27Ac signals, which are normally associated with active transcription. On the other hand, although lncRNAs often show less conservation compared with protein-coding genes, the lncRNA UBR5-AS1 presented a much strong sequence conservation that is nearly comparable to the proteins of P53R2 and UBR5 (scoring by 100 vertebrates Basewise Conservation by PhyloP). This result suggested that UBR5-AS1 may undergo evolutionary pressure for maintaining some important functions. In addition, in the gene-body region of UBR5-AS1, a great number of SINE and LTR repeats were found, both of which had been extensively proved to be associated with lncRNA’s regulatory function [41]. Next, we identified up to 20 cancer-related proteins co-expressed with UBR5-AS1 (Fig. 7b) and predicted the GO annotations of UBR5-AS1 via GO enrichment analysis (Fig. 7c), by using the co-expression sub-network centralized on UBR5-AS1. The Top10 (sorted by *p*-value, Fisher’s exact test) enriched GO terms showed that UBR5-AS1 was functionally relevant with ‘RNA splicing’, ‘leukocyte activation’, ‘immune system process’ and so on. All these findings indicate that UBR5-AS1 underlines a highly potential cancer-related lncRNA and is worthy of more intensive study.

## Conclusions

Based on the consideration of massive outbreak of cancer transcriptome data and the need of identification of cancer-related lncRNAs, as well as the disadvantage of current prediction model, in this work, we developed a novel machine-learning-based classifier -- CRlncRC -- for cancer-related lncRNAs, with integrating multiple features and optimizing algorithms to enhance its prediction performance. According to our results, CRlncRC has a significant preponderance of prediction sensitivity and accuracy over some previous models. Moreover, by using CRlncRC method, we predicted a set of cancer-related lncRNAs, all of which displayed a strong relevance to cancer as indicated by somatic mutation number, distance with genes encoding cancer-related proteins, differential expression fold-change between pairs of tumor and normal tissues, and GO enrichment analysis. Consequently, our predicted cancer-related lncRNAs could be a valuable conception for further cancer-related lncRNA function studies.

## Methods

### Construction of the positive and negative lncRNA sets

We manually reviewed more than 2500 published literature (Additional file 10), and finally collected 158 cancer-related lncRNAs as the positive set (Additional file 1). Cancer-related lncRNAs complied with the following standards: the selected lncRNAs were either differentially expressed in cancer (as verified by qRT-PCR), co-occurred with a significant pertinence to clinicopathological parameters (e.g., tumor differentiation, clinical stage, survival time); or else, were proven by functional experiments (e.g., colony formation assay, matrigel invasiveness assay, xenograft mouse model, and metastasis nude mouse model) to participate in cancer development.

To create the negative set, we located a large number of SNPs derived from NHGRI-EBI GWAS Catalog [42] into the sequences of lncRNAs, and selected only those in which no SNP was detected within 10 kb range as cancer-unrelated lncRNAs. We finally obtained 4553 lncRNAs as the negative set (Additional file 2). Since the size of the negative set greatly outnumbered the positive set, for pairwise comparison in the same dimension, 100 sub-negative sets were constructed by random sampling of 150 entries for 100 times from 4553 cancer-unrelated lncRNAs.

### Construction of the four categories of features

To reflect the differences between cancer-related lncRNAs and cancer-unrelated lncRNAs, we collected 85 features that could potentially facilitate the recognition of cancer-related lncRNAs and grouped them into 4 different categories (Additional file 3): Genomic features (18), Expression features (16), Epigenetic features (27), and Network features (24).

### Genomic features

1. GC content, which is much probable to influence the stability of gene [4], and gene splicing [18, 19]. According to gene structures, we considered five types of features, that is GC contents in TSS (transcription start site) up- and down-stream 1 kb/5 kb, gene body, exon, and intron.
2. Sequence conservation score. We considered sequence conservation in both lncRNA’s exon and intron as well as TSS up- and down-stream 1 kb, according to the phastCons scores pre-calculated by the UCSC genome database (https://genome.ucsc.edu).
3. Repeat. Recent research has revealed that repeat elements can play important roles in transcriptional and post-transcriptional regulation [43–48]. We extracted the number of LINEs, LTRs, Satellites and SINEs in either gene body or TSS up-stream /down-stream 1k as repeat features. The transposable elements were downloaded from UCSC genome database (genome version GRCh38/hg38, genome annotation version GENCODE v24).
4. MiRNA host. LncRNAs may host miRNA both within their exons and introns. We counted the number of miRNAs (obtained from miRBase, version 21) residing in the region of each lncRNA by using BEDTools [49].
5. Micropeptide. Functional micropeptides can be concealed within lncRNAs [50]. Meanwhile, the length of these short peptides is likely to affect the localization of lncRNAs. We obtained the short peptide sequence of each transcript from the LncRNAWiki [51] and calculated the average peptide length.

### Expression features

We intend to comprehensively depict the highly temporal and spatial expression specificity of lncRNAs, with the multi-tissue data as complete as possible. The expression profiles of 16 different tissue types were downloaded from Human Body Map project [52] and normalized by our in-house scripts with DEseq [53] method. The 16 different tissue types include adipose, adrenal gland, brain, breast, colon, heart, kidney, leukocyte, liver, lung, lymph node, ovary, prostate gland, skeletal muscle, testis, thyroid gland.

### Epigenetics features

The importance of maintaining or reprogramming histone methylation appropriately is illustrated by links to disease and aging, or possibly transmission of traits across generations [54]. For example, Wan at al. found that lncRNAs may be transcriptionally regulated by histone modification in Alzheimer’s Disease [55]. Here, we obtained nine epigenetics tracks. They are three types of epigenetic signals (H3k4me1, H3k4me3, and H3k27ac) in three types of cell lines (Gm12878, K562, and H1hesc) from UCSC genome database. The average epigenetic signals were calculated on gene body, TSS up- and down-stream 1 kb/5 kb, respectively.

### Network features

We constructed a gene co-expression network between protein-coding and lncRNA genes from the above normalized expression profiles. Spearman’s rank correlation coefficient (SCC, cutoff scc-value = 0.6) was used for calculating the correlation of each gene pair across the samples. Then we achieved three types of features of co-expression network:

1. Co-expression with cancer driver genes. The SCC values with Top20 mutational hotspots cancer driver genes were used as network features. These cancer driver genes were downloaded from http://cancerhotspots.org, including BRAF, CDKN2A, CTNNB1, EGFR, ERBB2, FBXW7, GNAS, H3F3A, HRAS, IDH1, KRAS, NRAS, PIK3CA, PTEN, RAC1, SF3B1, TP53, and U2AF1.
2. Co-expression interactions with cancer-related proteins. We calculated the number of interactions between lncRNA and cancer-related protein-coding genes in the co-expression network. The cancer-related protein-coding gene list is downloaded from Cancer Gene Census (https://cancer.sanger.ac.uk/census).
3. Total degree in co-expression network. Hub genes in the gene network usually means functional important genes. Thus we checked the number of neighbors in co-expression network of each lncRNA.

We also investigated the miRNA-target interaction network between miRNA lncRNA. miRNAs are higher relevant to cancer, with many key effects on various biological processes, e.g., embryonic development, cell division, differentiation, and apoptosis, are widely recognized [56, 57]. We downloaded cancer-related miRNA from HMDD v2.0 [58]. For each lncRNA, we counted the number of its regulatory cancer-related miRNAs, as well as that of all the involved miRNAs. We download the interaction information between miRNA and lncRNA from starBase [57].

### Machine learning algorithms

Scikit-learn [59] is a python package that exposes a wide variety of machine learning algorithms which enabling easy comparison of methods. We use five machine learning algorithms in this package to train and validate our data. The detail algorithms parameter can be found in Additional file 11. The python script we performed our analysis can be found in Github (https://github.com/xuanblo/CRlncRC).

### Coding-lncRNA gene co-expression network construction

A gene co-expression network was constructed between protein-coding and lncRNA genes from the above normalized expression profiles. We calculated the Spearman’s correlation coefficient and its corresponding *P*-value (Eq. 1) between the expression profiles of each gene-pair using the in-house Perl script. Only gene-pair with an adjusted *P*-value of 0.01 or less and with a Spearman’s correlation coefficient no less than 0.6 is regarded as co-expression in our coding-lncRNA gene co-expression network.

1 $$ \left\{\begin{array}{l}\mathrm{Rs}=\frac{\sum_i\left({x}_i-\overline{x}\right)\left({y}_i-\overline{y}\right)}{\sqrt{\sum_i{\left({x}_i-\overline{x}\right)}^2{\sum}_i{\left({y}_i-\overline{y}\right)}^2}}\\ {}\mathrm{F}\left({\mathrm{R}}_{\mathrm{s}}\right)=\frac{1}{2}\ln \frac{1+\mathrm{Rs}}{1-\mathrm{Rs}}\\ {}\mathrm{Z}=\sqrt{\frac{\mathrm{n}\hbox{-} 3}{1.06}}\mathrm{F}\left(\mathrm{Rs}\right)\end{array}\right. $$

Where x or y represents the vector of the ranked expression value of each gene, Rs is the Spearman’s correlation coefficient between x and y, *x*_*i*_ or *y*_*i*_ stands for the rank of each expression value, $\overline{x}$ or $\overline{y}$, is the mean value of these ranks. F(Rs) is the Fisher transformation of Rs, and n is the sample size i.e. the vector length. The corresponding *P*-value of each Rs is calculated from Z, which is a z-score for Rs that approximately follows a standard normal distribution under the null hypothesis of statistical independence [60, 61].

### LncRNA functional annotation

The GO annotation of protein coding-genes was downloaded from Gene Ontology Consortium (only biological process annotations were considered). While, GO annotation of lncRNA was predicted using the GOATOOLS (version 0.6.4) [62], which determines the GO annotation of one gene in our network according to the GO annotations of its immediate neighbor genes (*P*-value < 0.05).

## Additional files

Additional file 1: Positive lncRNA dataset. (XLSX 10 kb)

Additional file 2: Negative lncRNA dataset. (XLSX 64 kb)

Additional file 3: Features importance. (XLSX 11 kb)

Additional file 4: TANRIC lncRNA dataset. (XLSX 176 kb)

Additional file 5: Model evaluate indicators. (DOCX 85 kb)

Additional file 6: ROC curve of combined feature class. (PDF 19 kb)

Additional file 7: Cumulative percentage curve of features. (PDF 208 kb)

Additional file 8: Gene and transcript length distribution. (PDF 14 kb)

Additional file 9: Predict results. (XLSX 29 kb)

Additional file 10: Cancer-related lncRNA Papers. (XLSX 326 kb)

Additional file 11: Model parameters. (PDF 46 kb)

## Abbreviations

AUC: Area under the ROC curve
BP: Biological Process
GO: Gene Ontology
KNN: K-Nearest Neighbors
LINE: Long interspersed nuclear elements
lncRNA: Long non-coding RNA
LR: Logistic Regression
NB: Naive bayes
RF: Random Forest
ROC: Receiver operating characteristic
SCC: Spearman’s rank correlation coefficient
SINE: Short Interspersed Nuclear Elements
SVM: Support Vector Machines
