# Supplementary material for: CRlncRC: a machine learning-based method for cancer-related long noncoding RNA identification using integrated features
Source: BMC Med Genomics. 2018 Dec 31;11(Suppl 6):120. doi: 10.1186/s12920-018-0436-9 (PMC6311943; doi:10.1186/s12920-018-0436-9)
Supplement: Supplementary file 5 — Model evaluate indicators. (DOCX 85 kb) [file 12920_2018_436_MOESM5_ESM.docx]

Table1 Performance of different algorisms (average Precision/Recall/Accuracy/AUC).

| Evaluation indicators | RF | NB | SVM | LR | KNN |
| --- | --- | --- | --- | --- | --- |
| Precision | 0.76(+/-0.02) | 0.71(+/-0.02) | 0.76(+/-0.04) | 0.75(+/-0.03) | 0.68(+/-0.03) |
| Recall | 0.75(+/-0.02) | 0.74(+/-0.01) | 0.69(+/-0.04) | 0.68(+/-0.03) | 0.56(+/-0.04) |
| Accuracy | 0.75(+/-0.02) | 0.71(+/-0.02) | 0.73(+/-0.03) | 0.71(+/- 0.02) | 0.63(+/-0.03) |
| AUC mean | 0.82(+/-0.02) | 0.78(+/-0.02) | 0.79(+/-0.03) | 0.76(+/-0.03) | 0.68(+/-0.03) |
| AUC confidence interval^*^ | 0.8154,0.8225 | 0.7751,0.7822 | 0.7811,0.7909 | 0.7530,0.7638 | 0.6703,0.6825 |

^*^0.95 confidence interval

Table2 Performance of different features (average Precision/Recall/Accuracy/AUC).

| Evaluation indicators | All features | expression | network | epigenetic | genomic |
| --- | --- | --- | --- | --- | --- |
| Precision | 0.76(+/-0.02) | 0.71(+/-0.02) | 0.68(+/-0.03) | 0.70(+/-0.02) | 0.68(+/-0.03) |
| Recall | 0.75(+/-0.03) | 0.75(+/-0.03) | 0.71(+/-0.03) | 0.72(+/-0.03) | 0.69(+/-0.03) |
| Accuracy | 0.75(+/-0.02) | 0.71(+/-0.02) | 0.68(+/-0.02) | 0.70(+/-0.02) | 0.67(+/-0.03) |
| AUC mean | 0.82(+/-0.02) | 0.76(+/- 0.03) | 0.73(+/- 0.03) | 0.76(+/- 0.02) | 0.73(+/-0.02) |
| AUC confidence interval^*^ | 0.8121,0.8189 | 0.7520,0.7619 | 0.7250,0.7362 | 0.7540,0.7624 | 0.7291,0.7385 |

^*^0.95 confidence interval
