# Supplementary figures and images for: CRlncRC: a machine learning-based method for cancer-related long noncoding RNA identification using integrated features
Source: BMC Med Genomics. 2018 Dec 31;11(Suppl 6):120. doi: 10.1186/s12920-018-0436-9 (PMC6311943; doi:10.1186/s12920-018-0436-9)

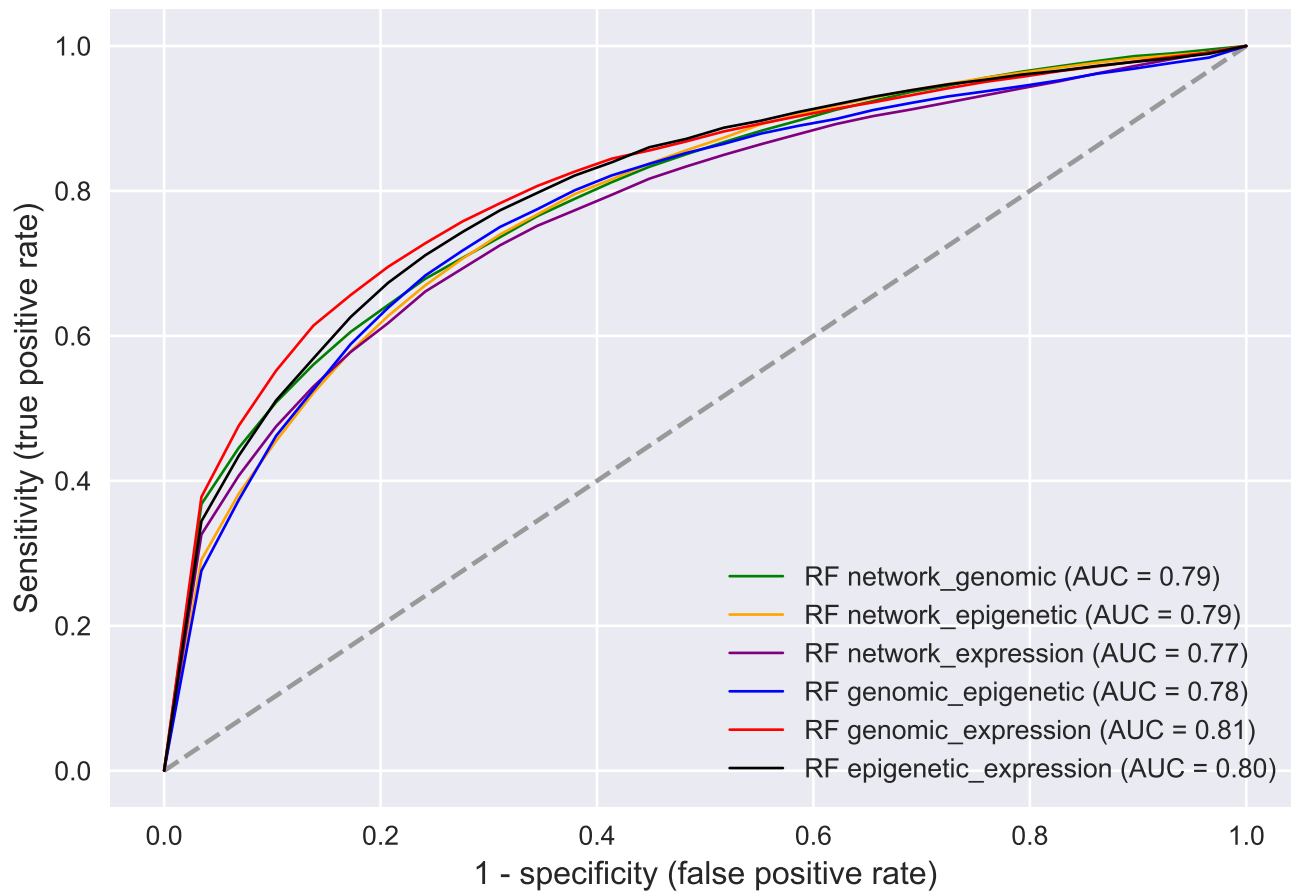

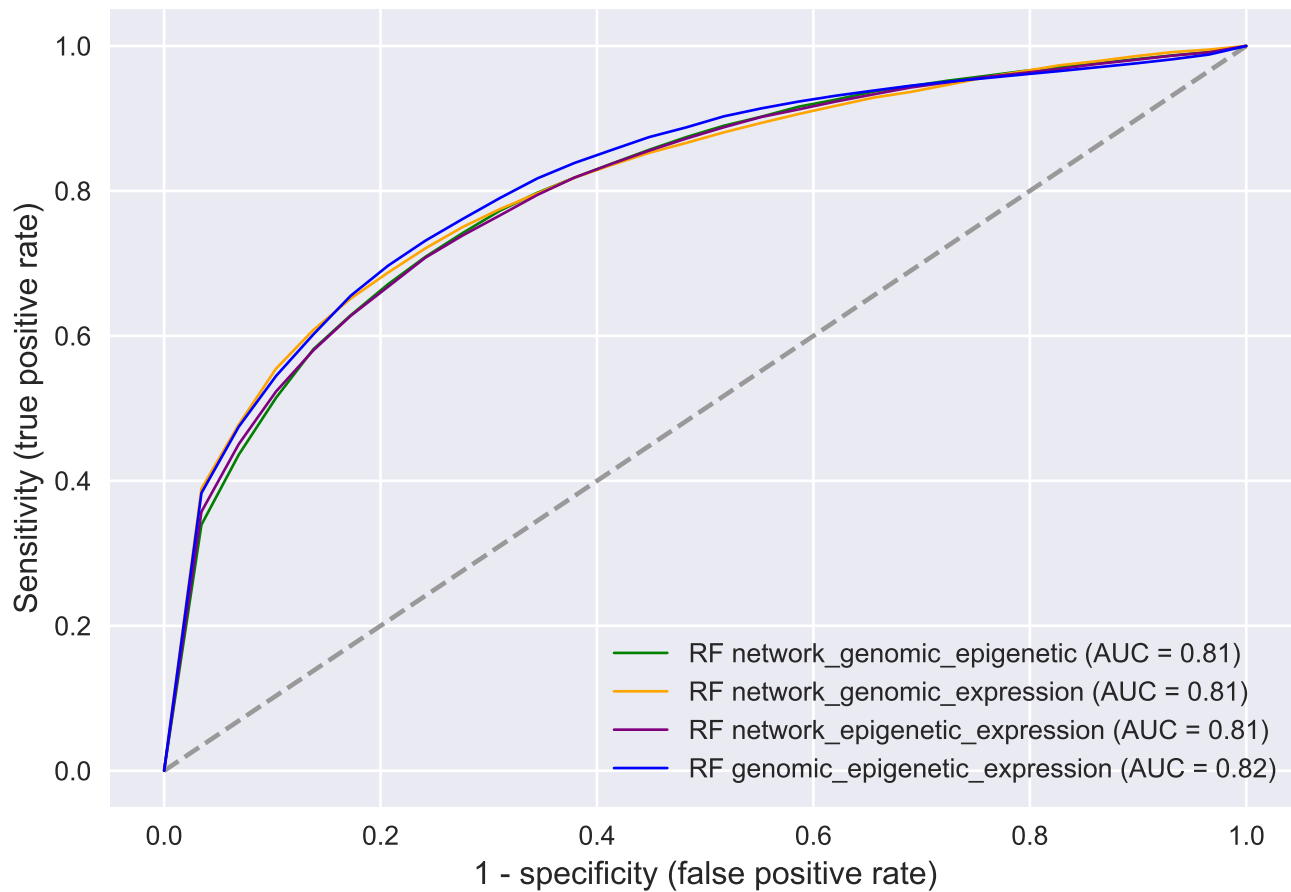

Supplement: Supplementary file 6 — ROC curve of combined feature class. (PDF 19 kb) [file 12920_2018_436_MOESM6_ESM.pdf]

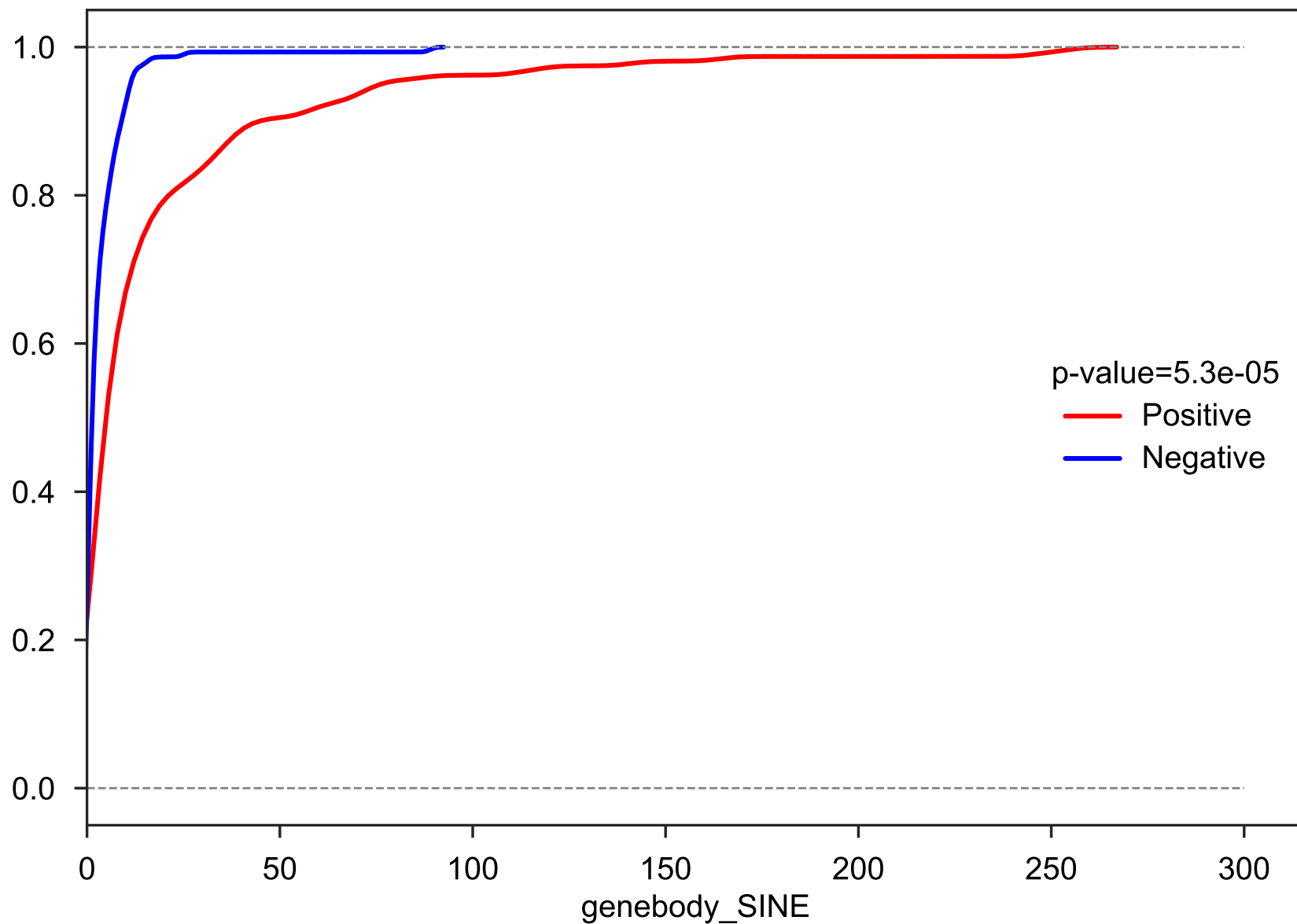

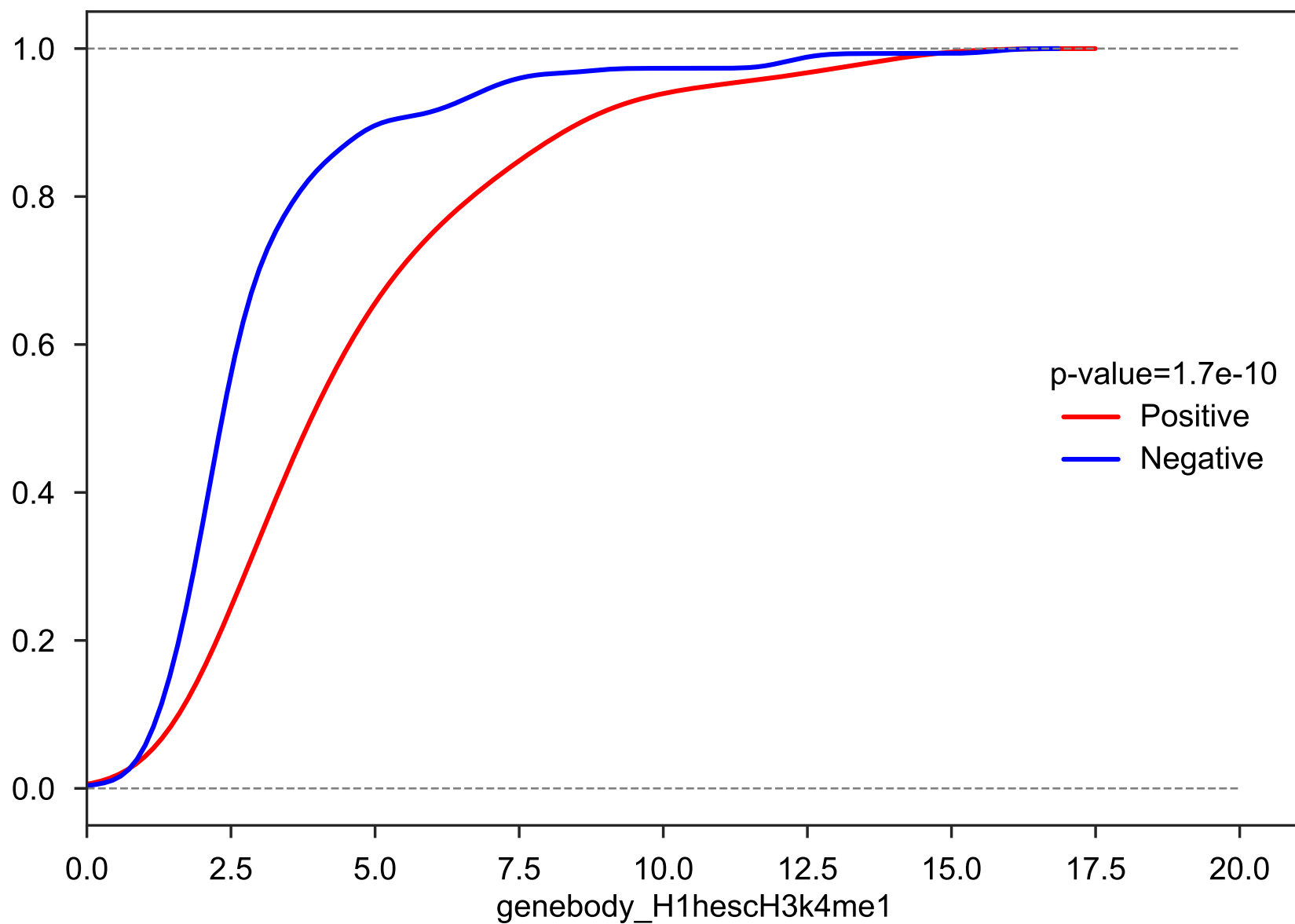

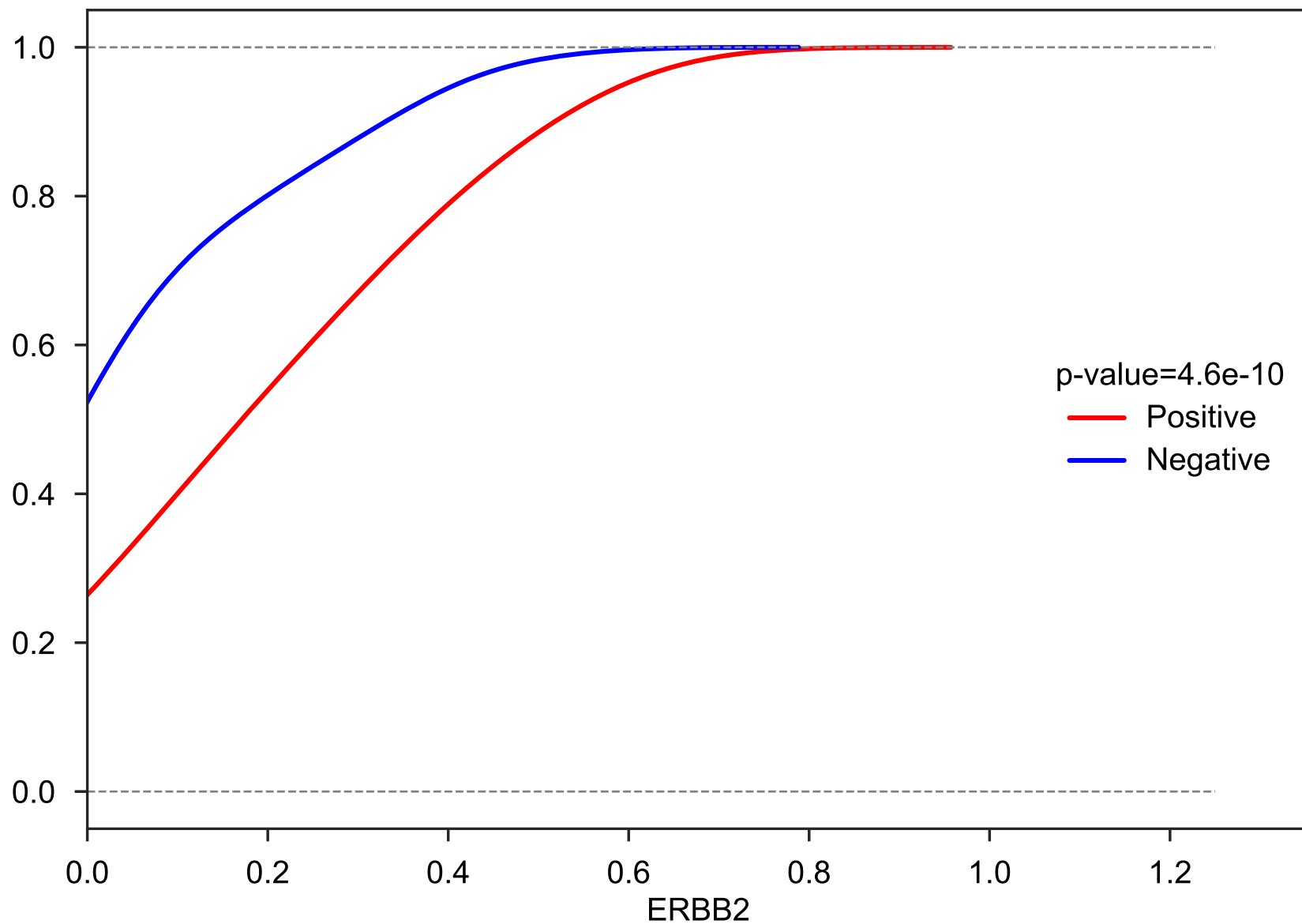

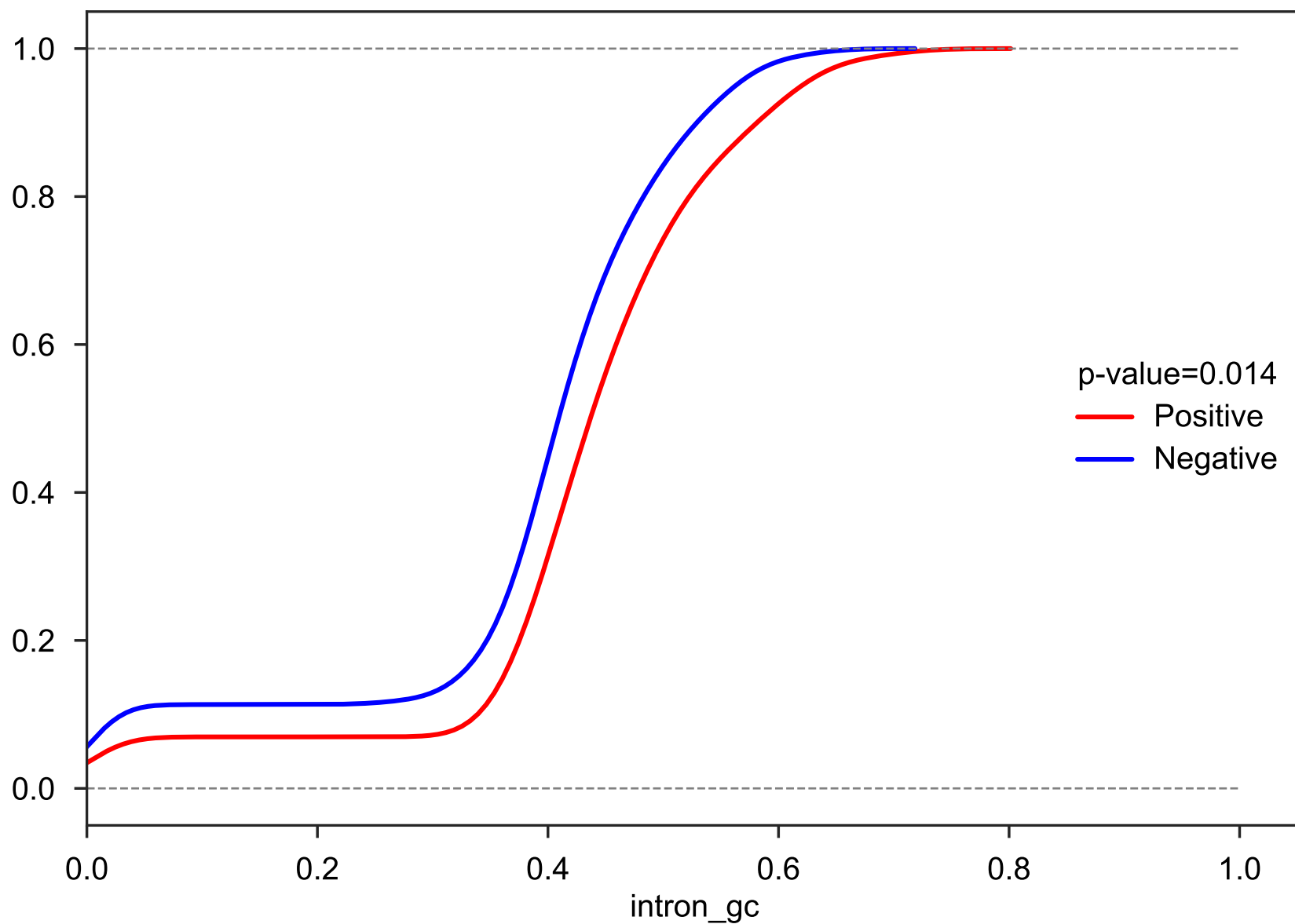

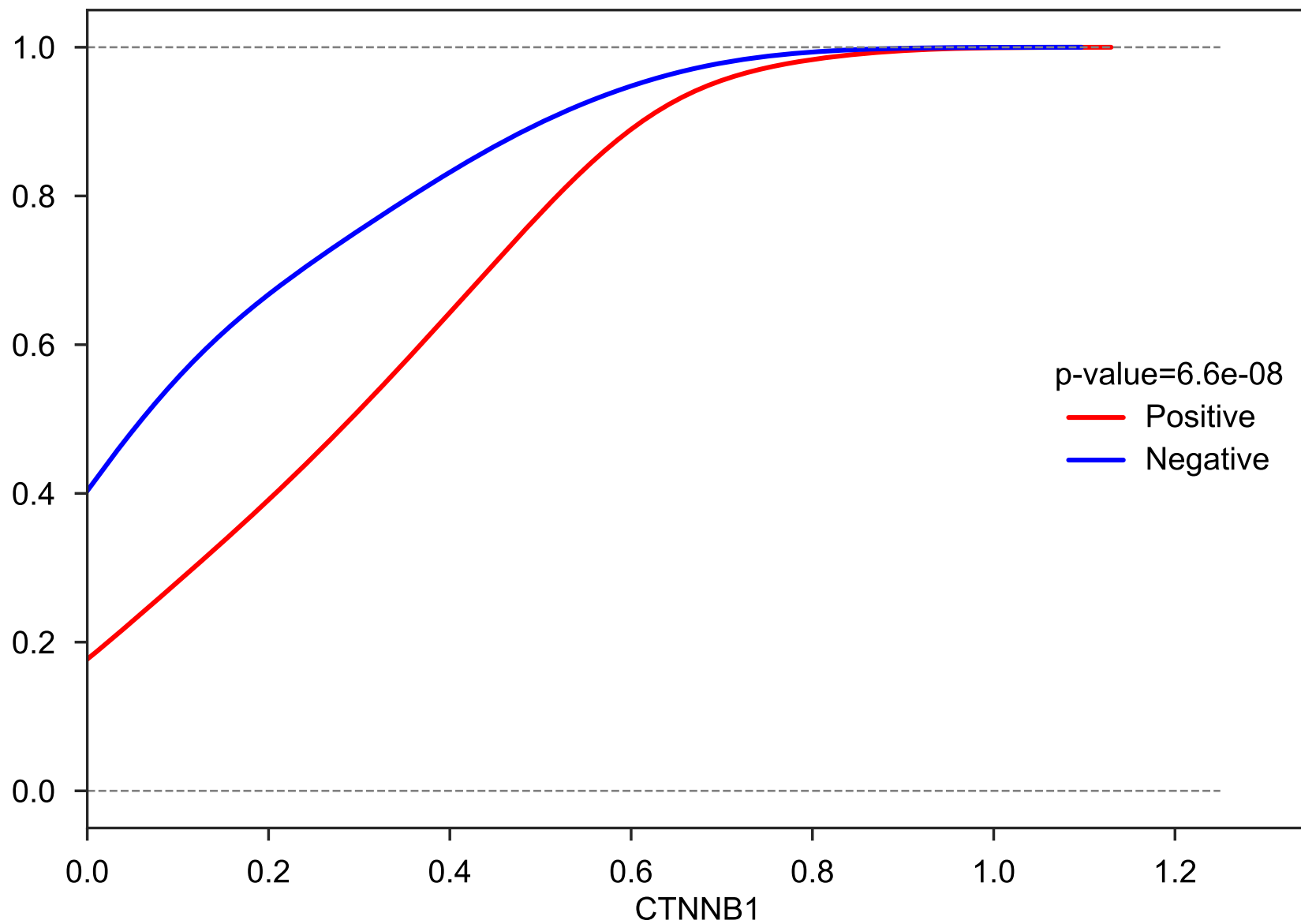

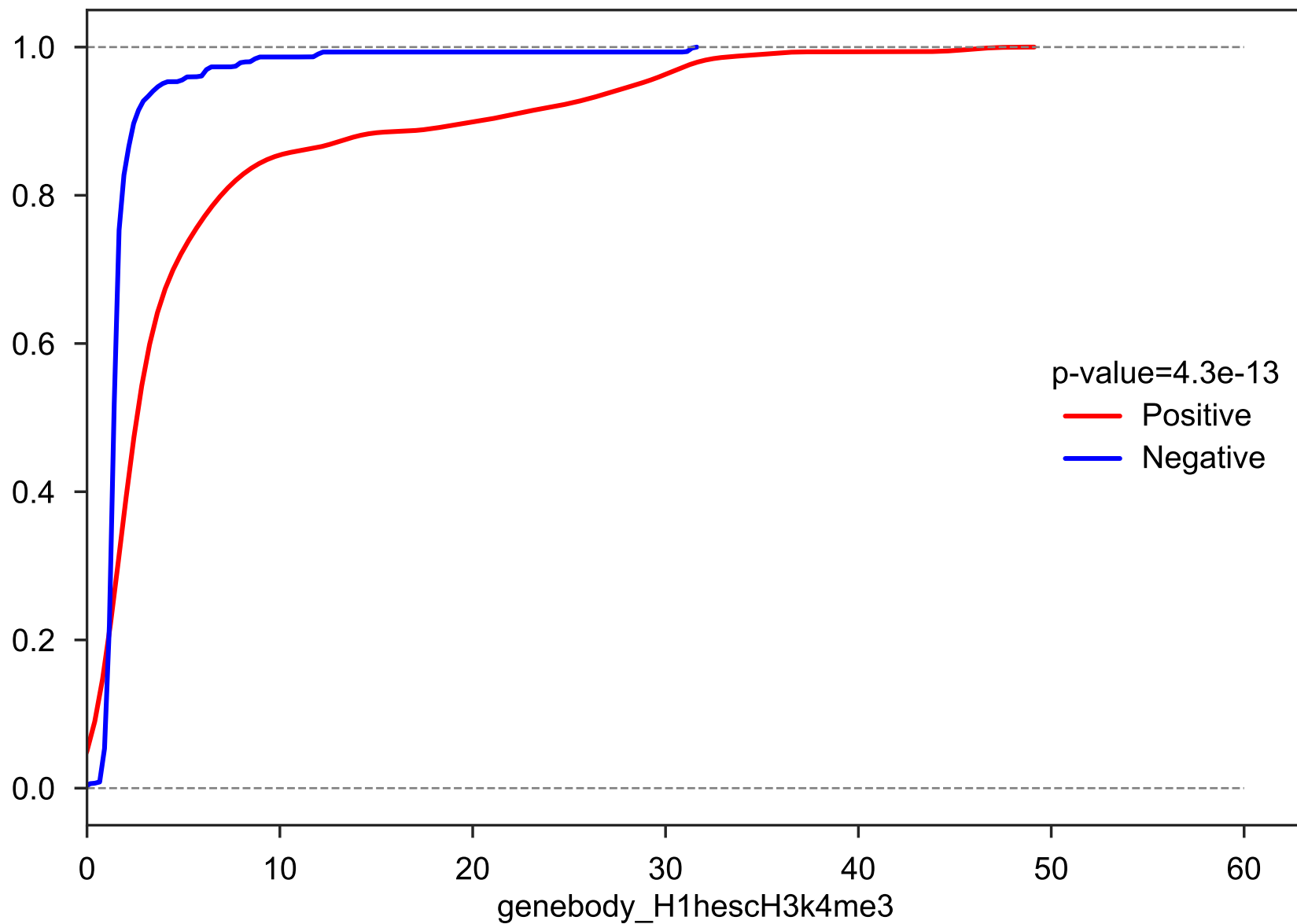

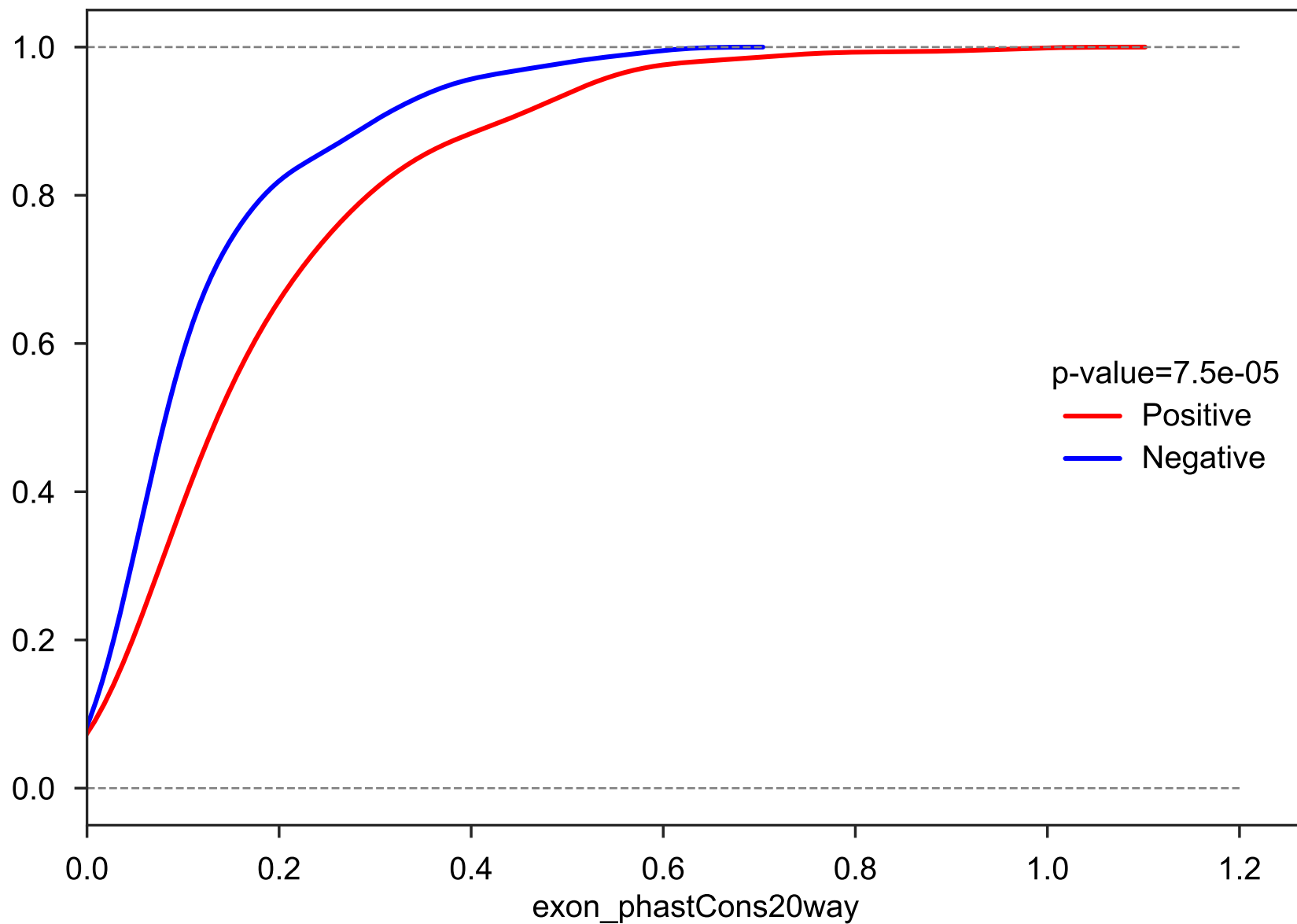

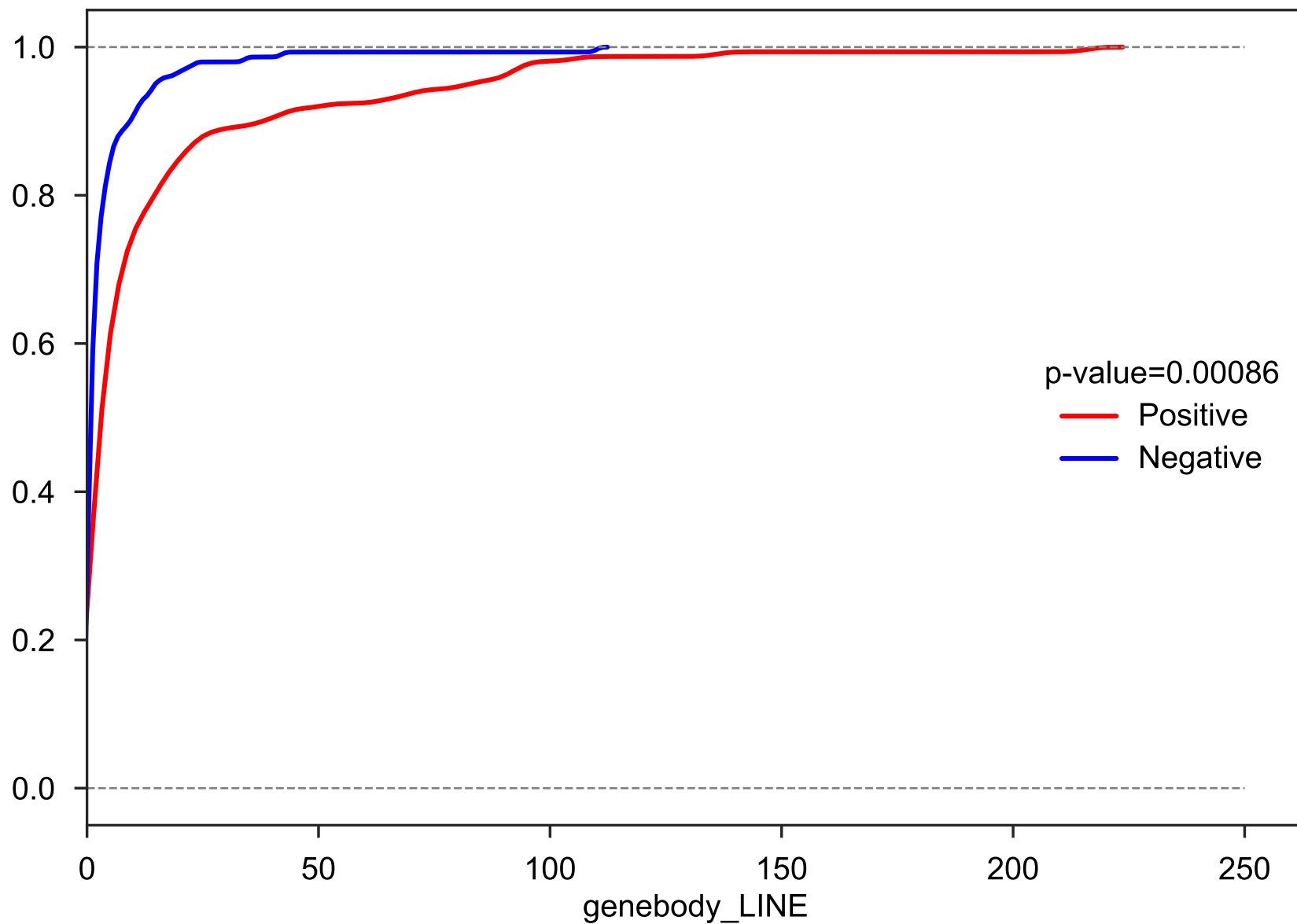

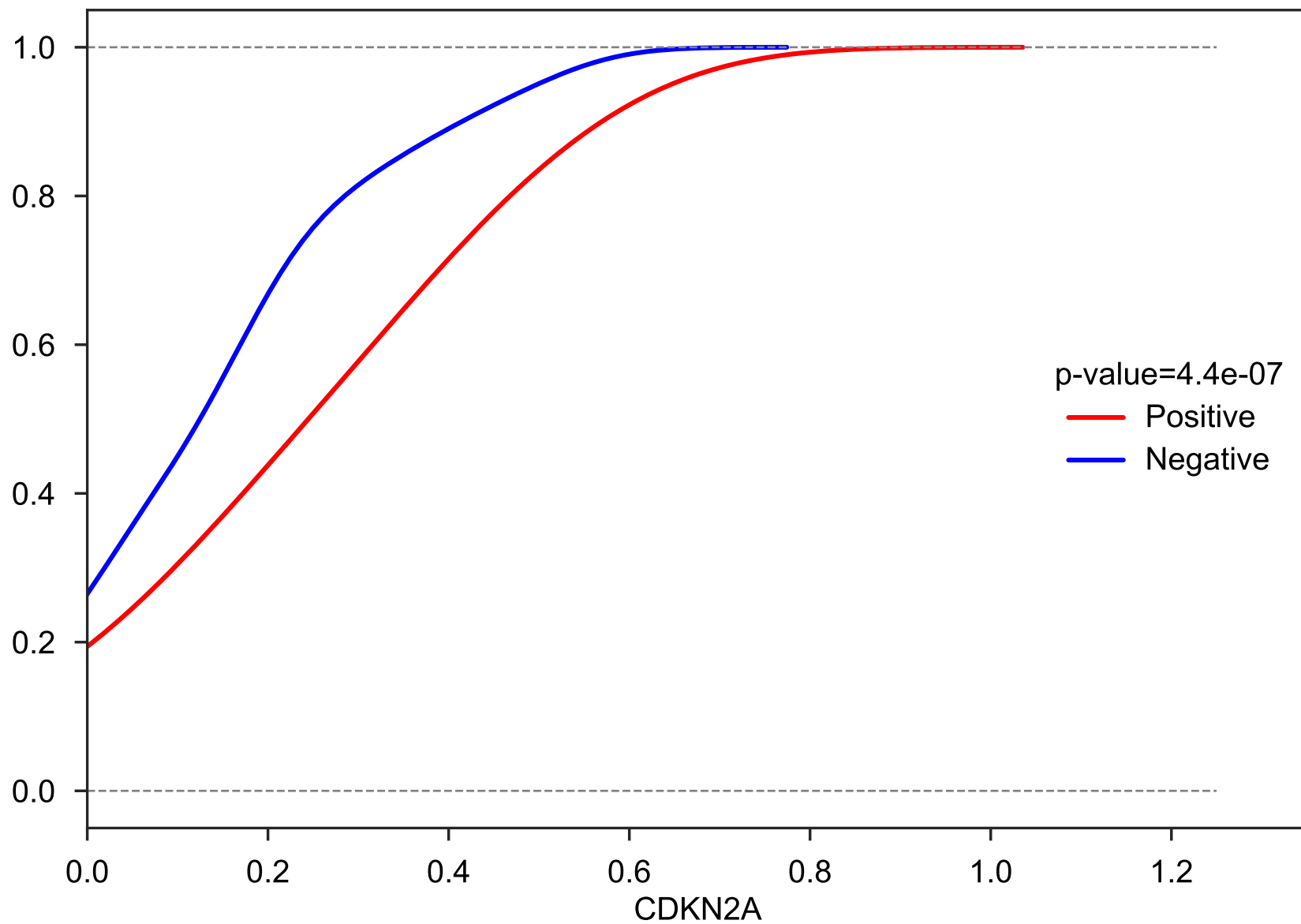

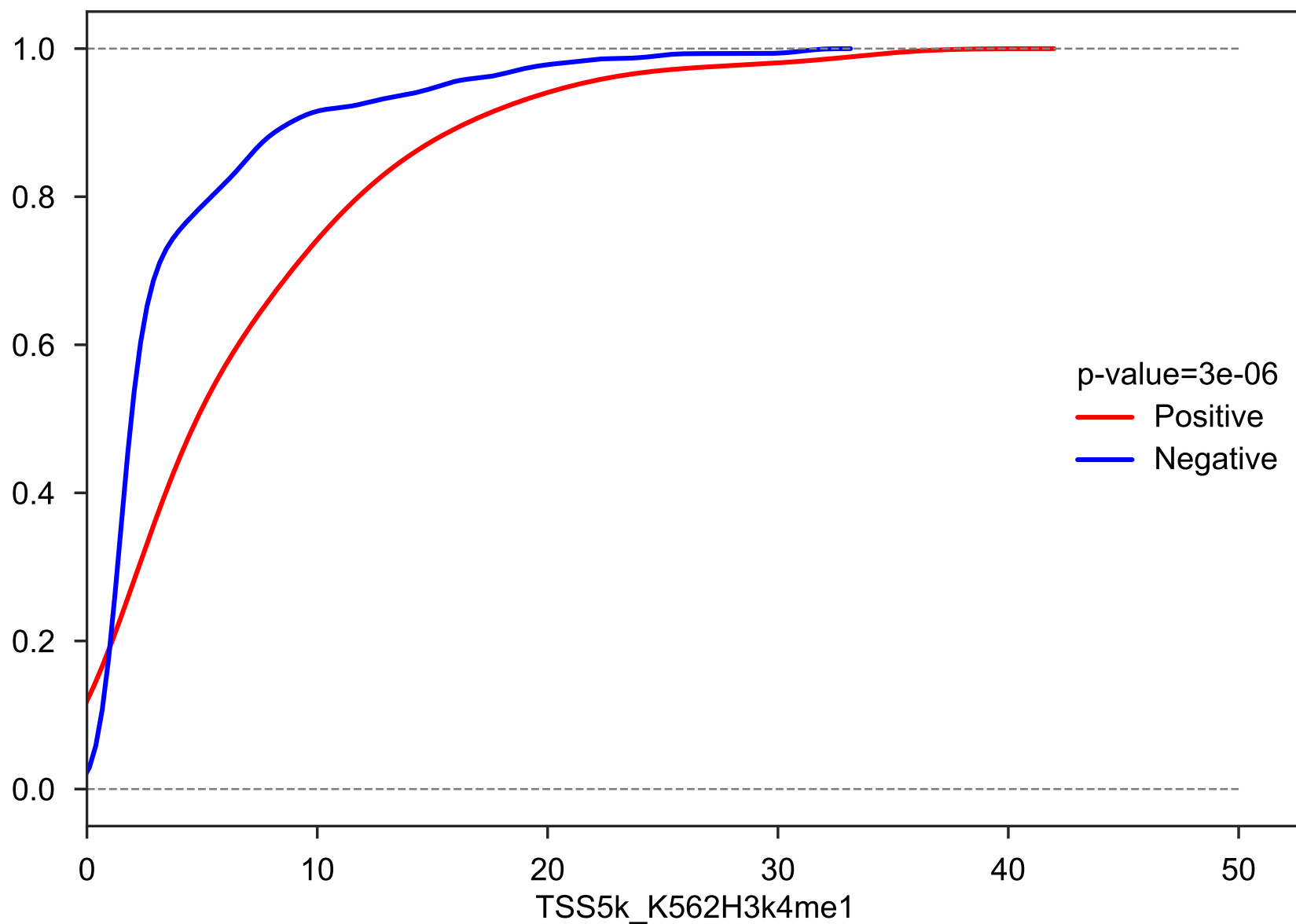

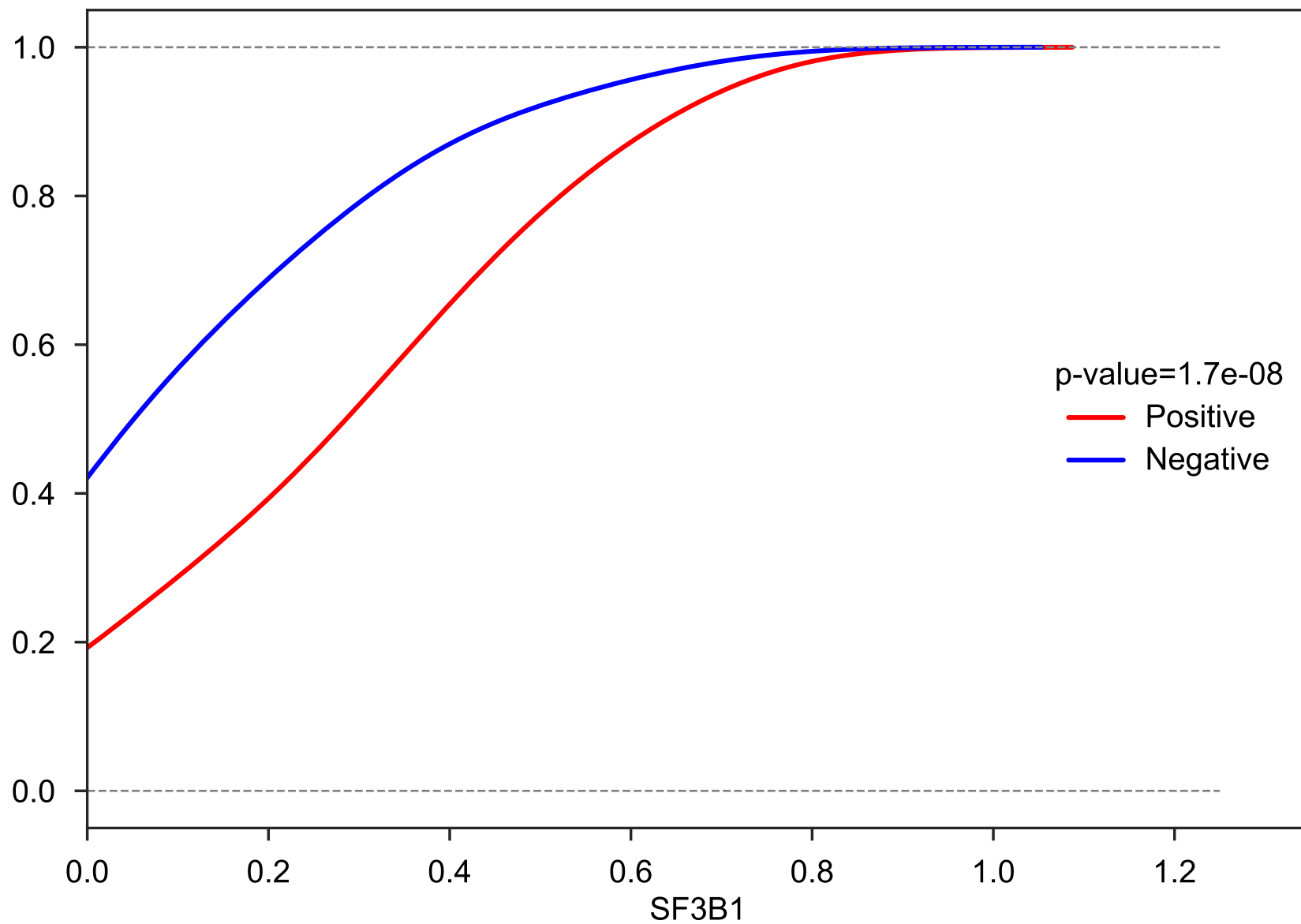

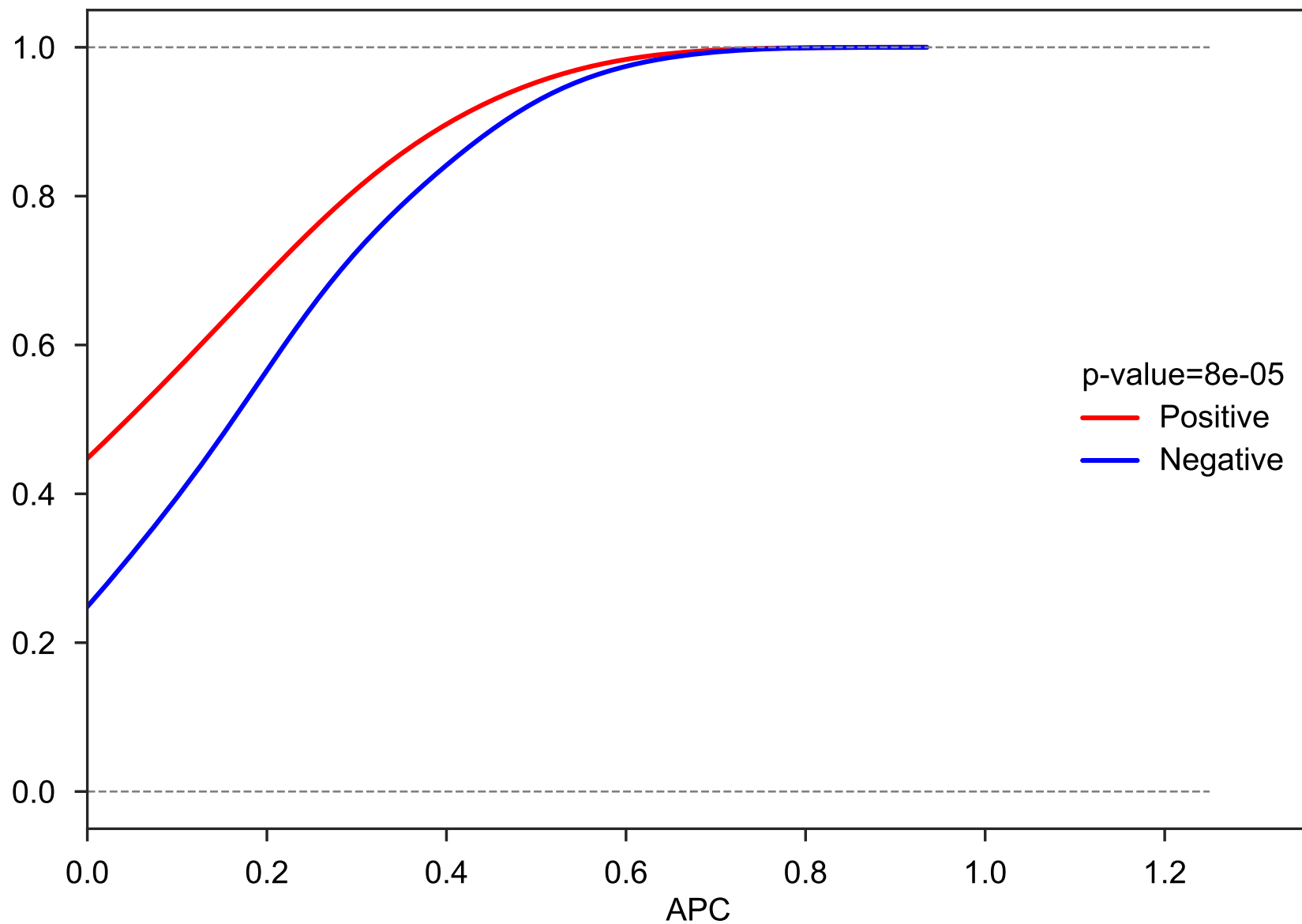

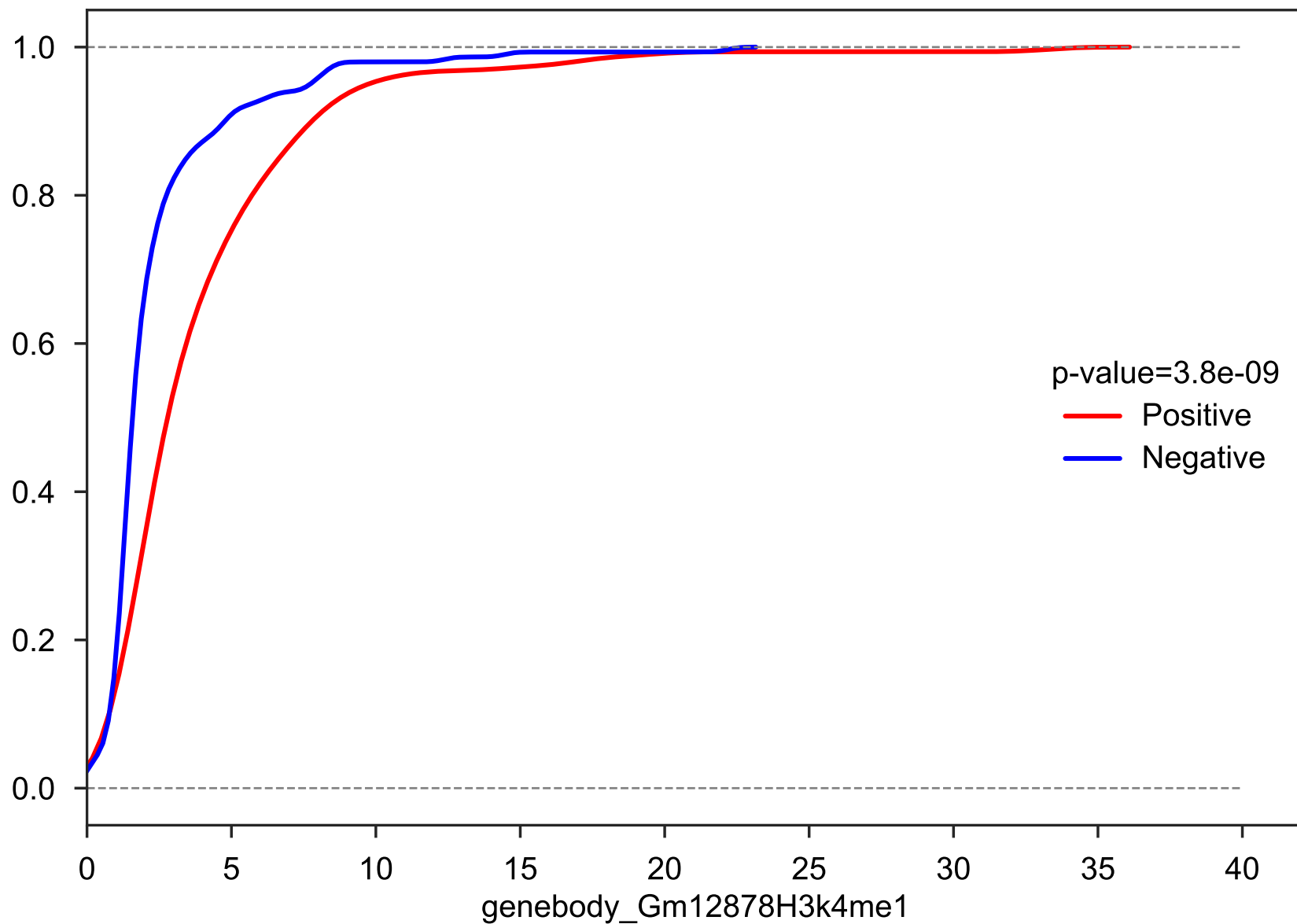

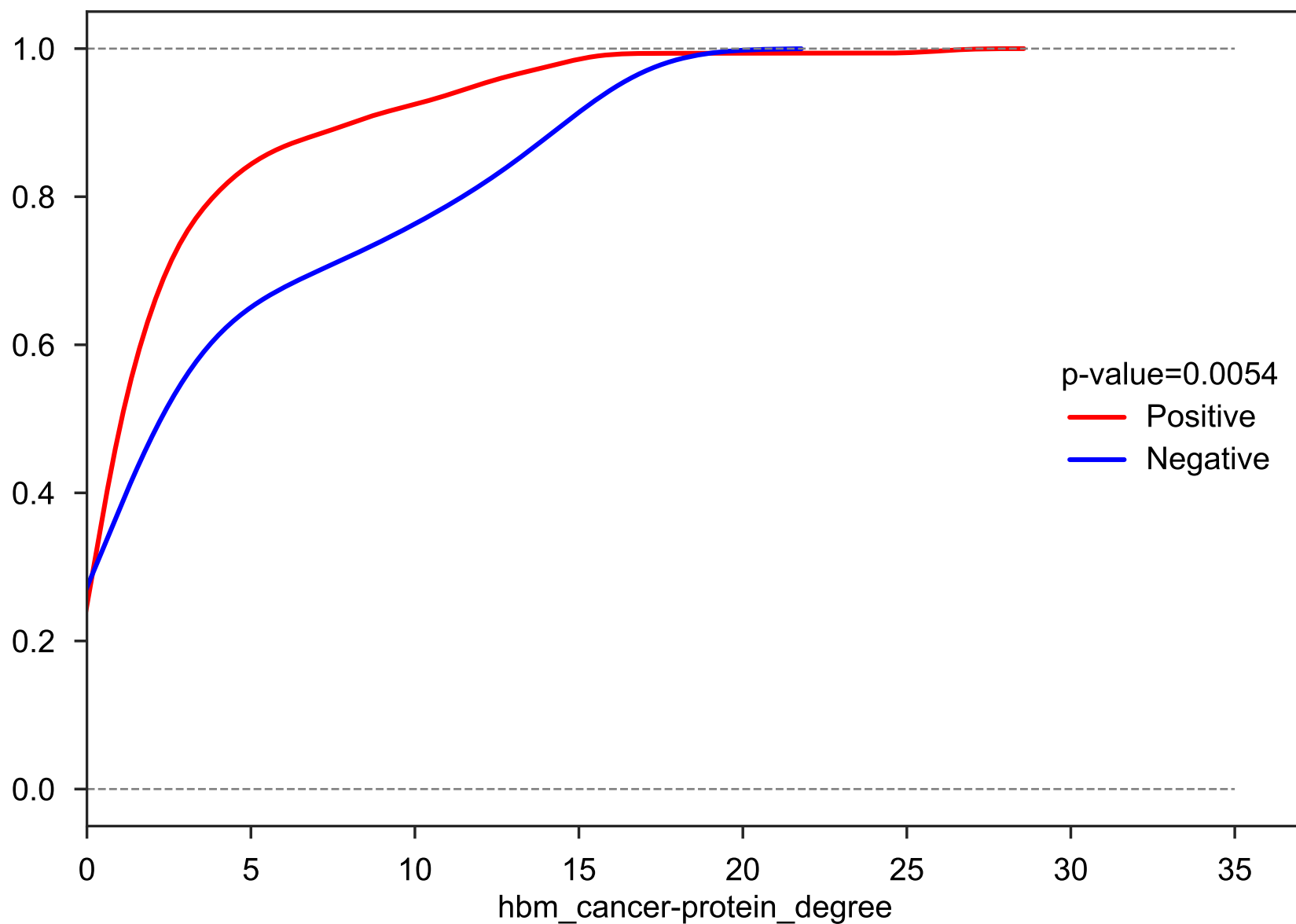

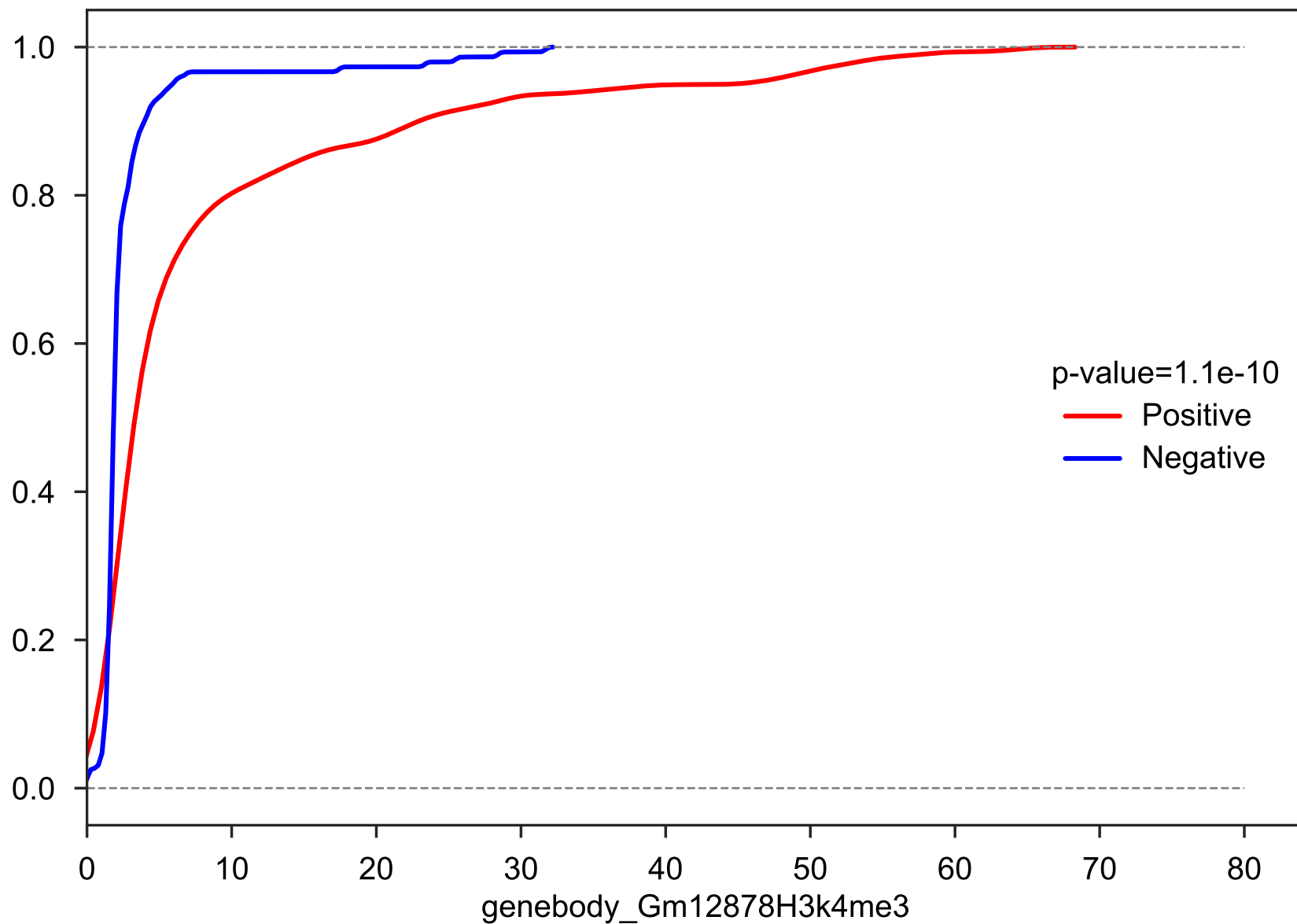

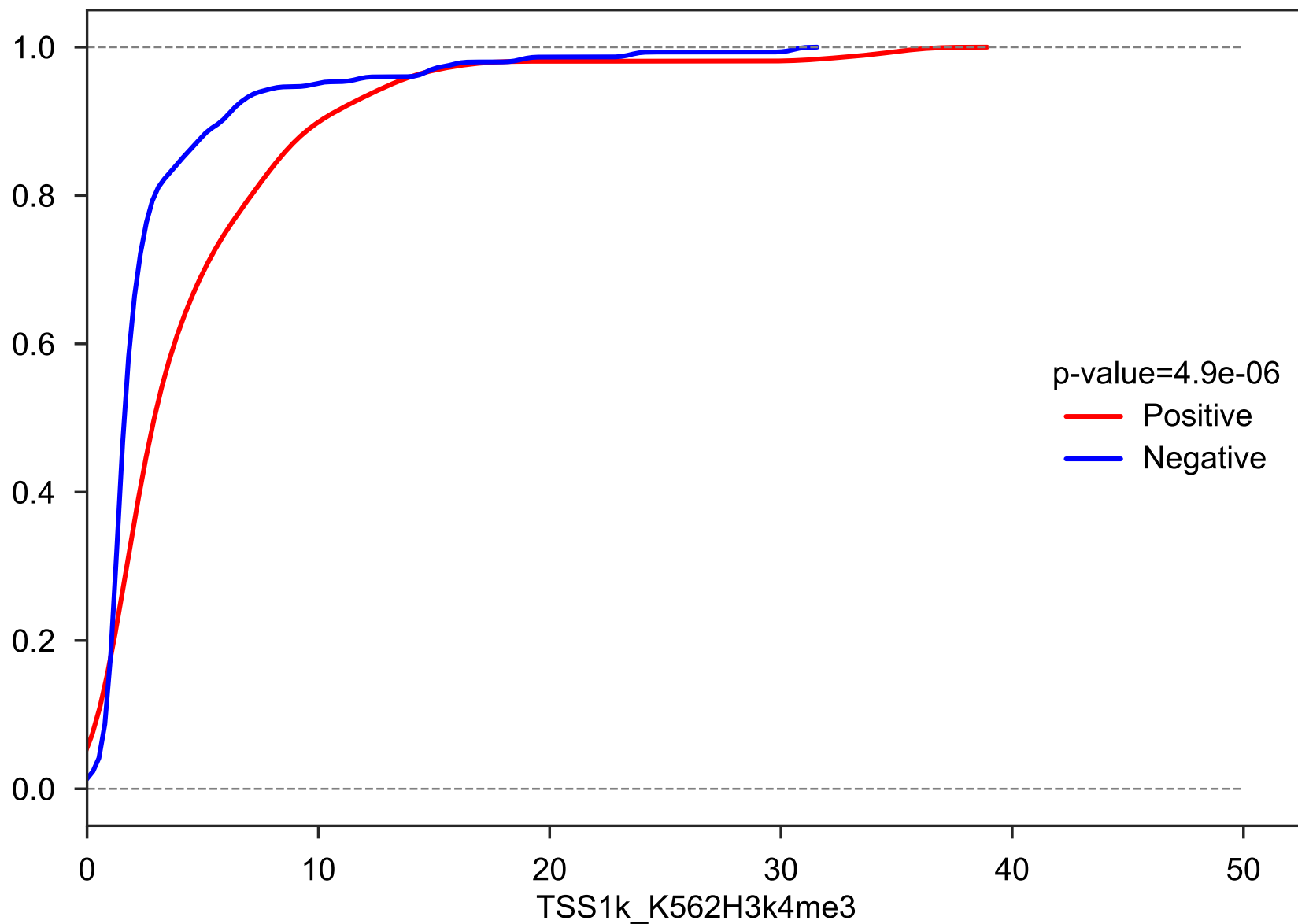

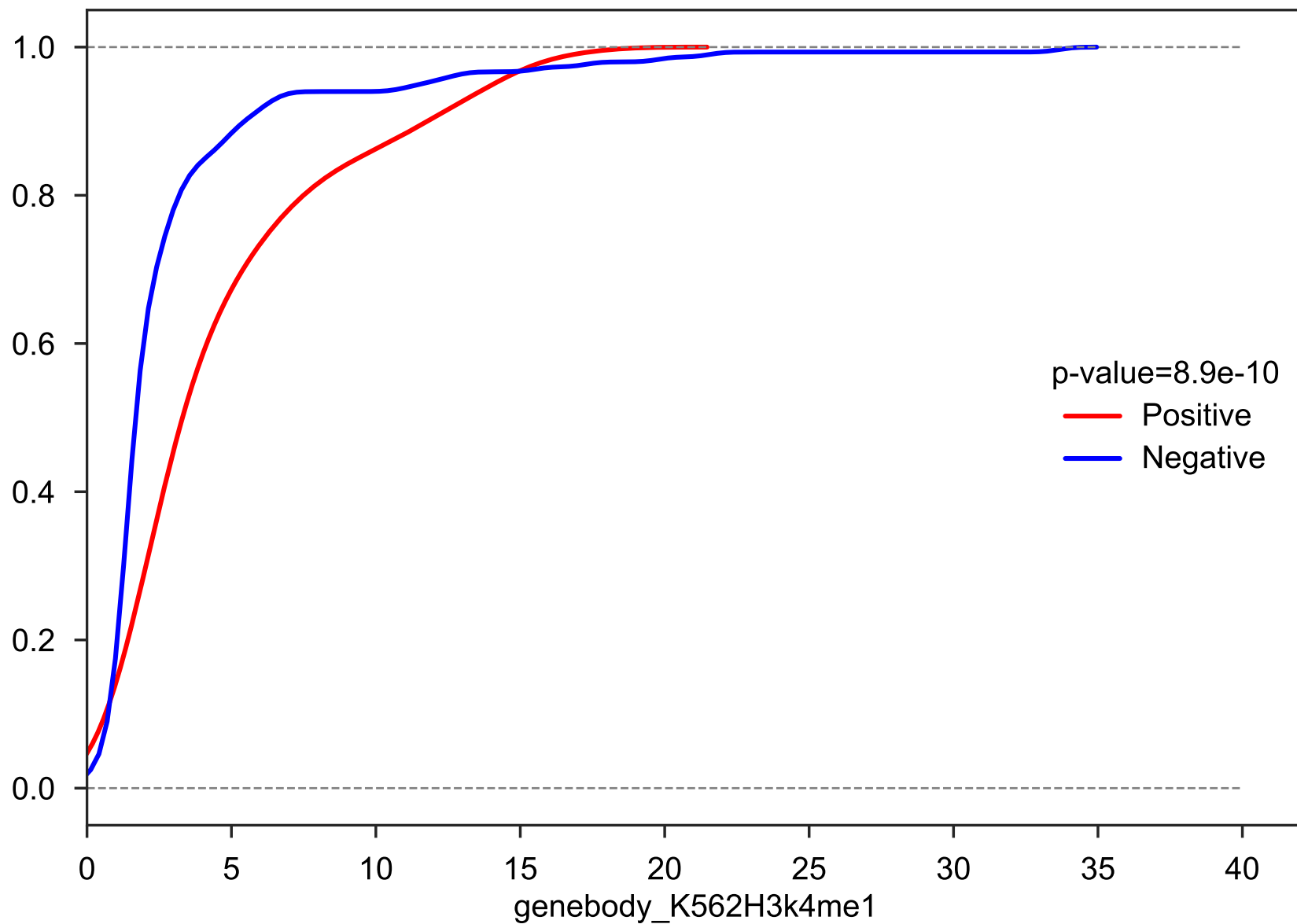

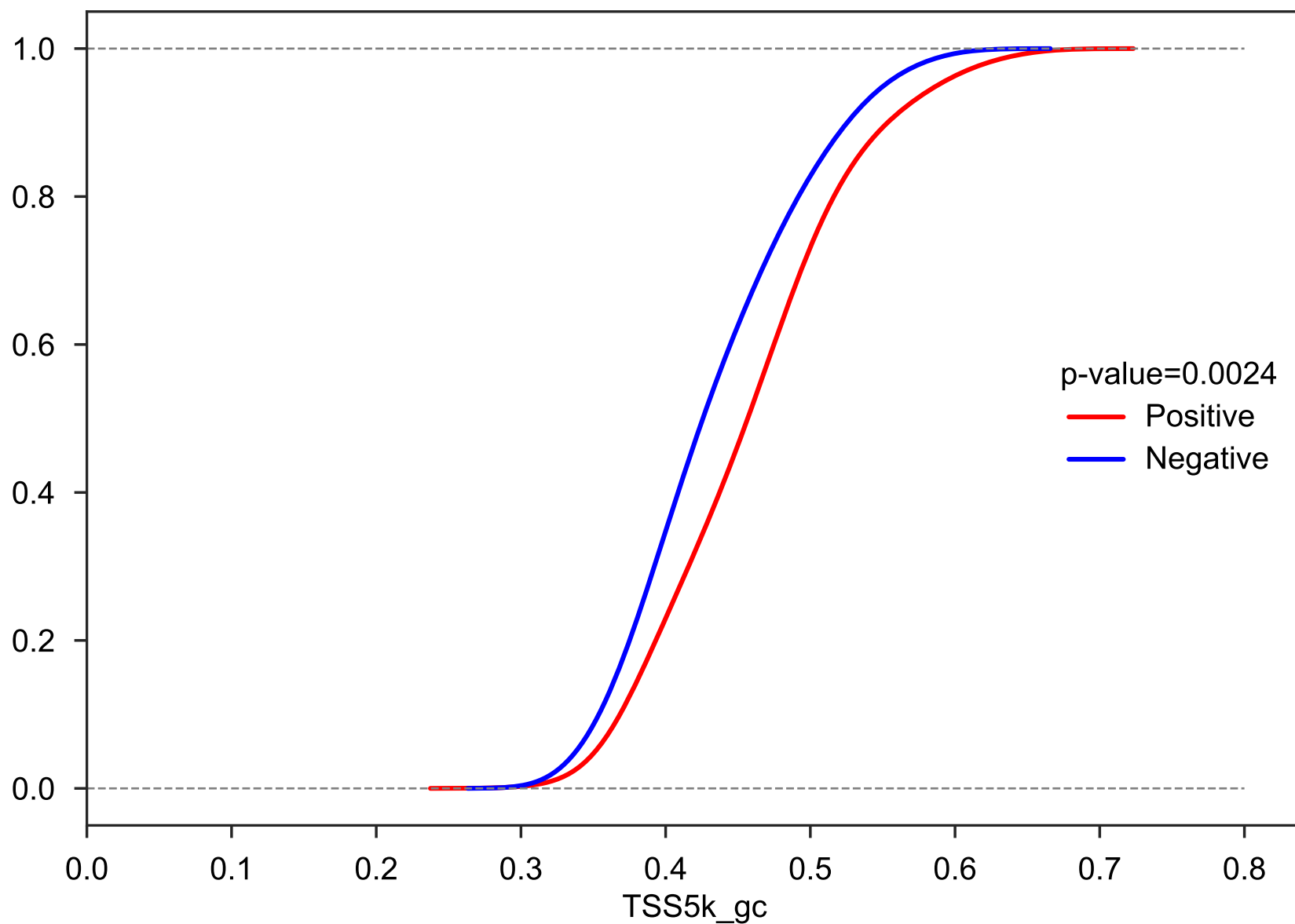

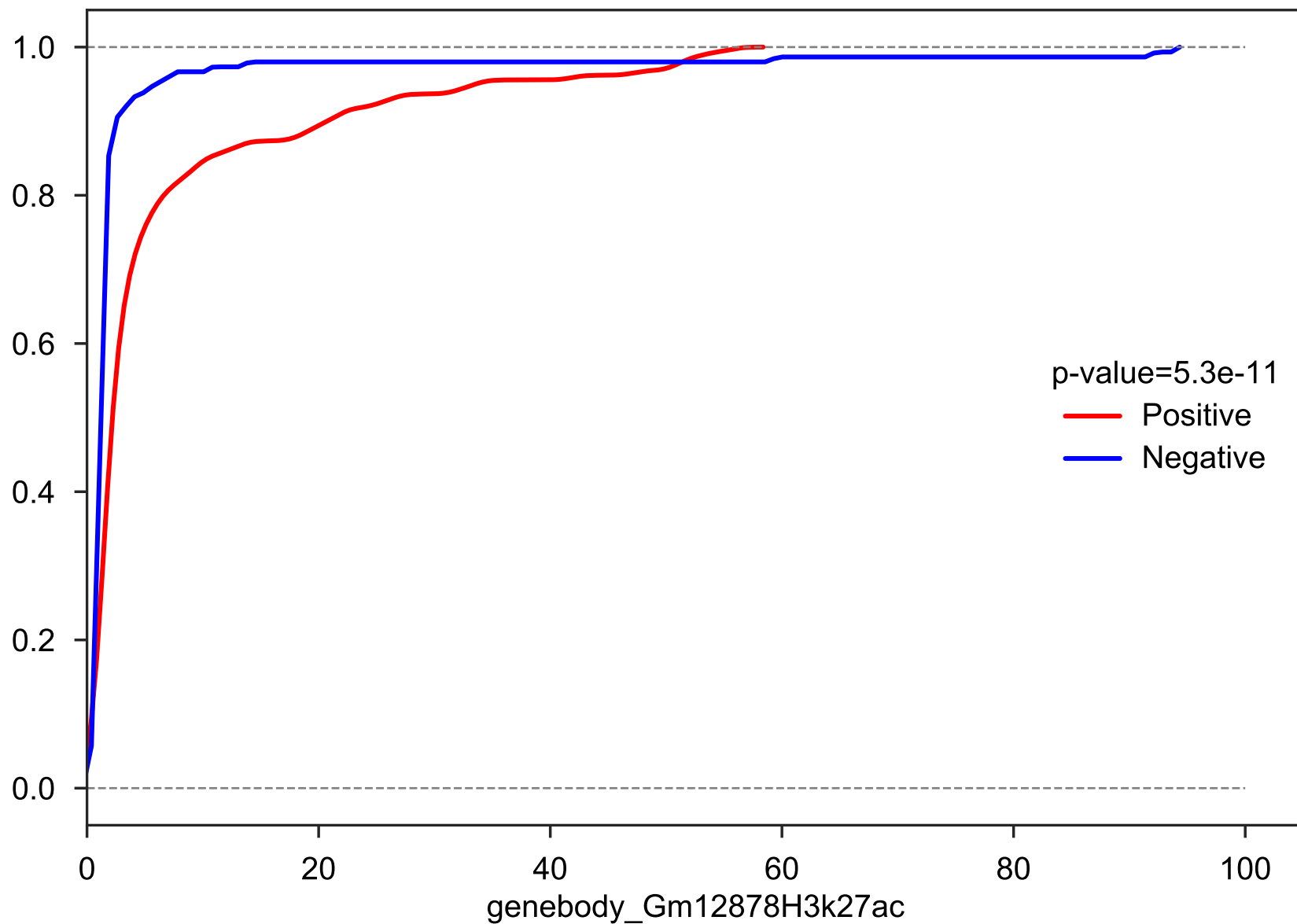

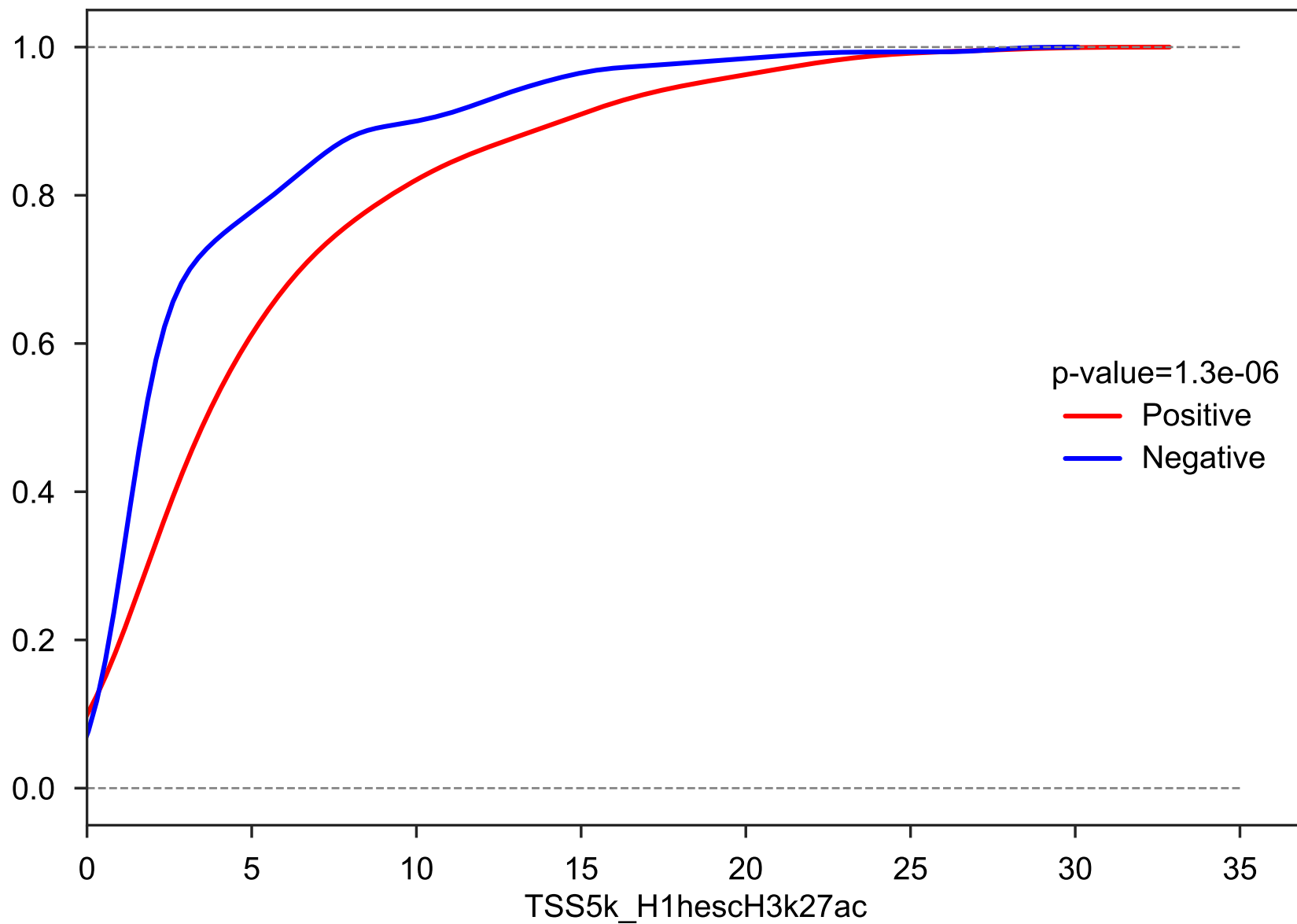

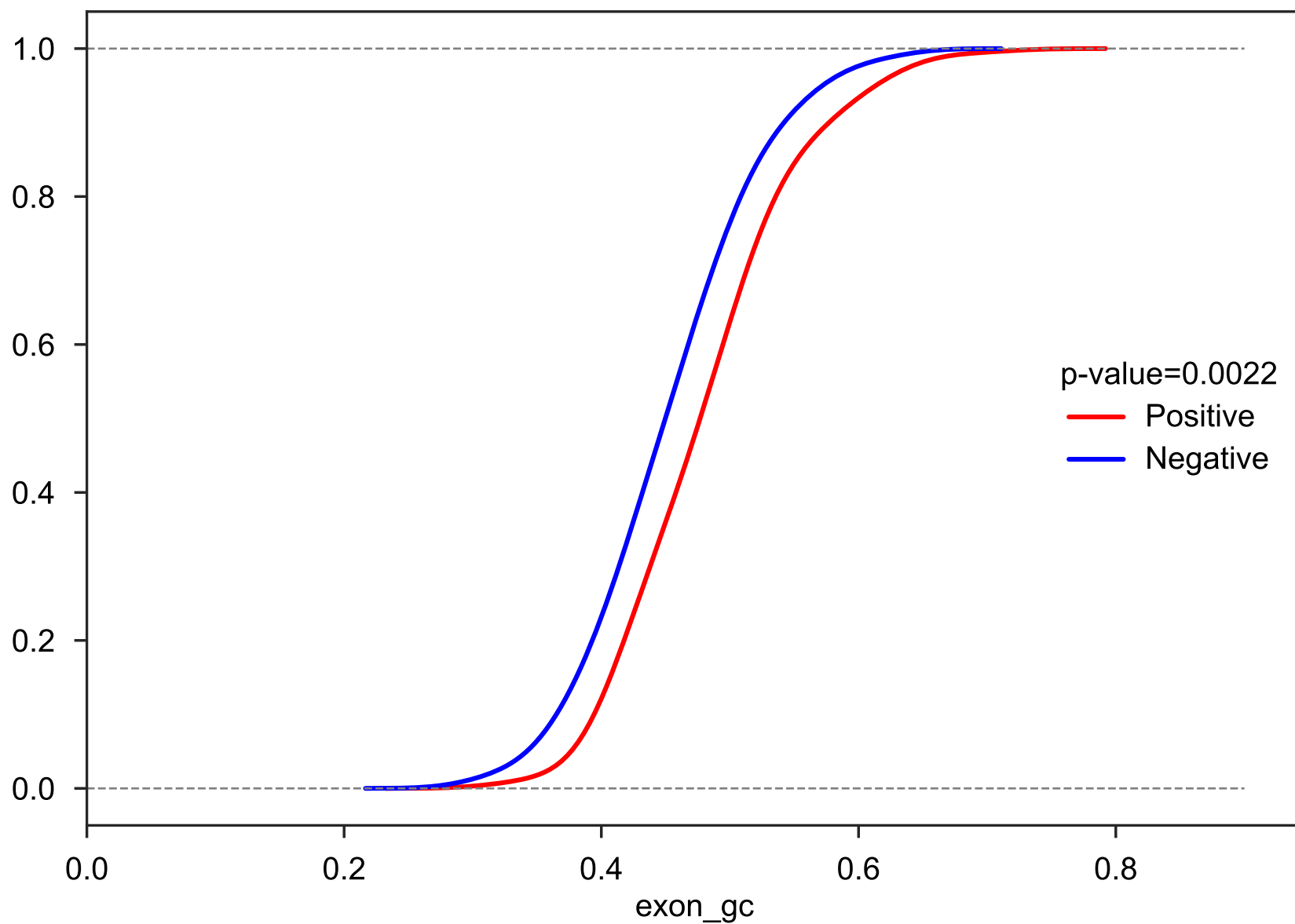

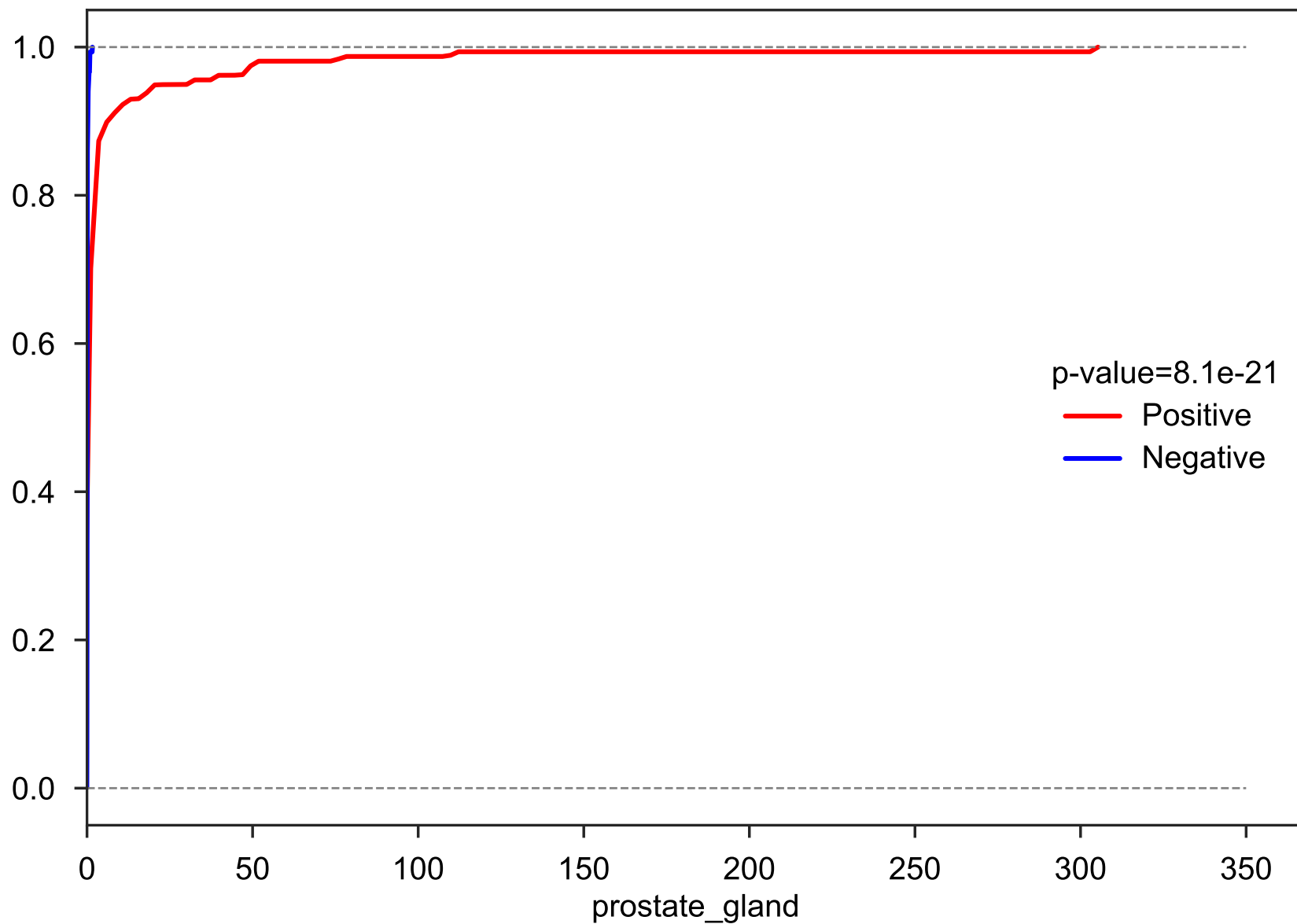

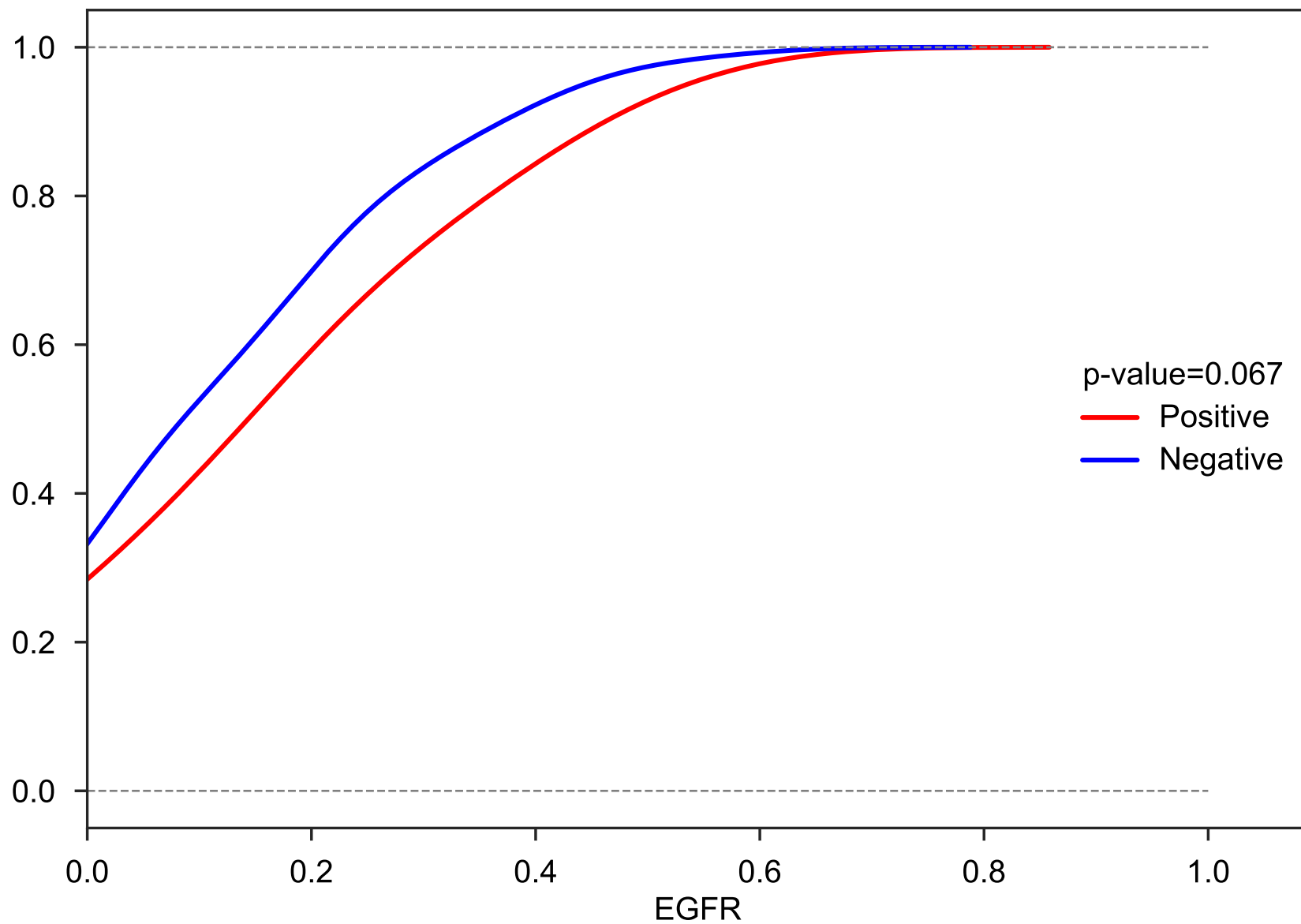

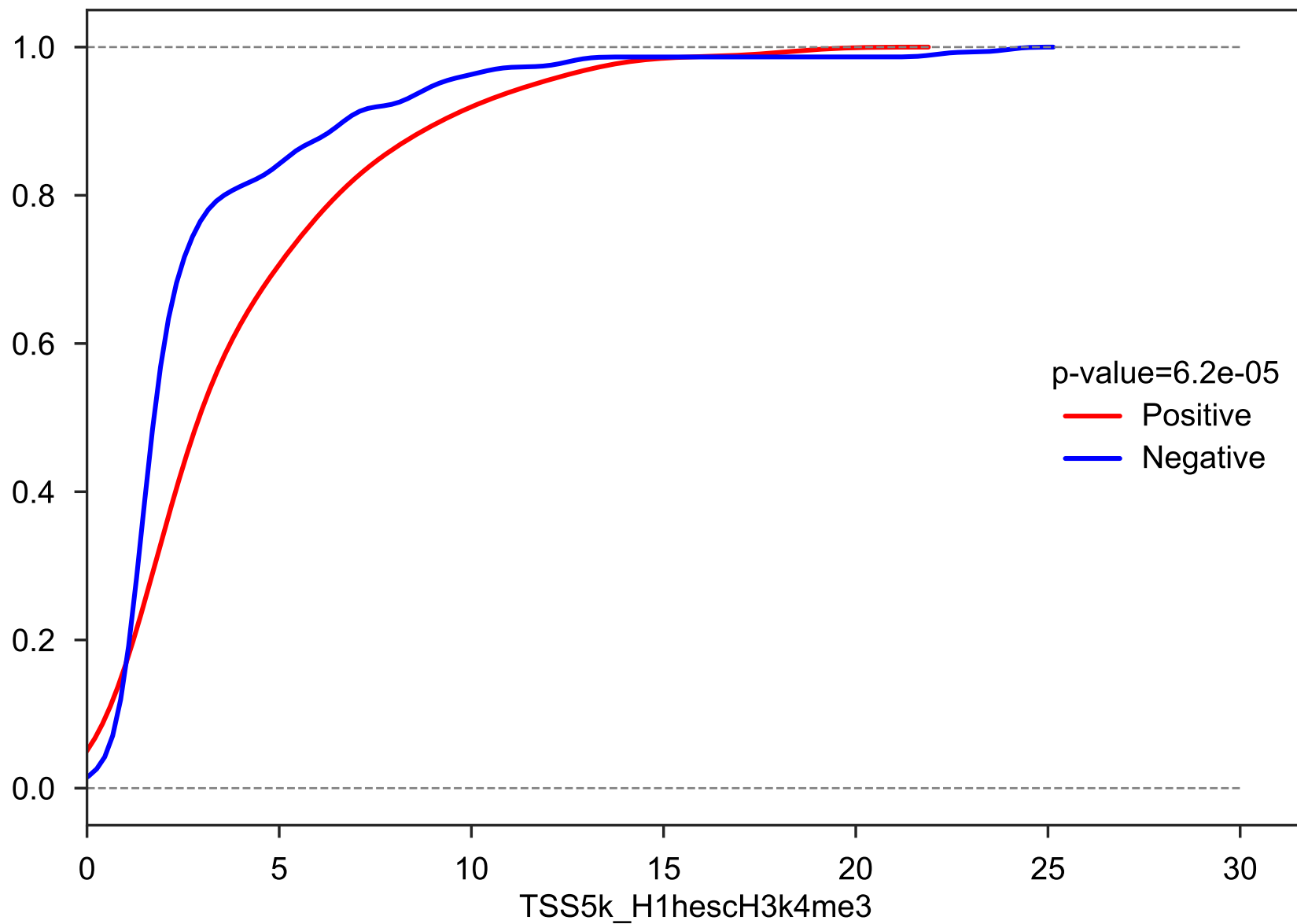

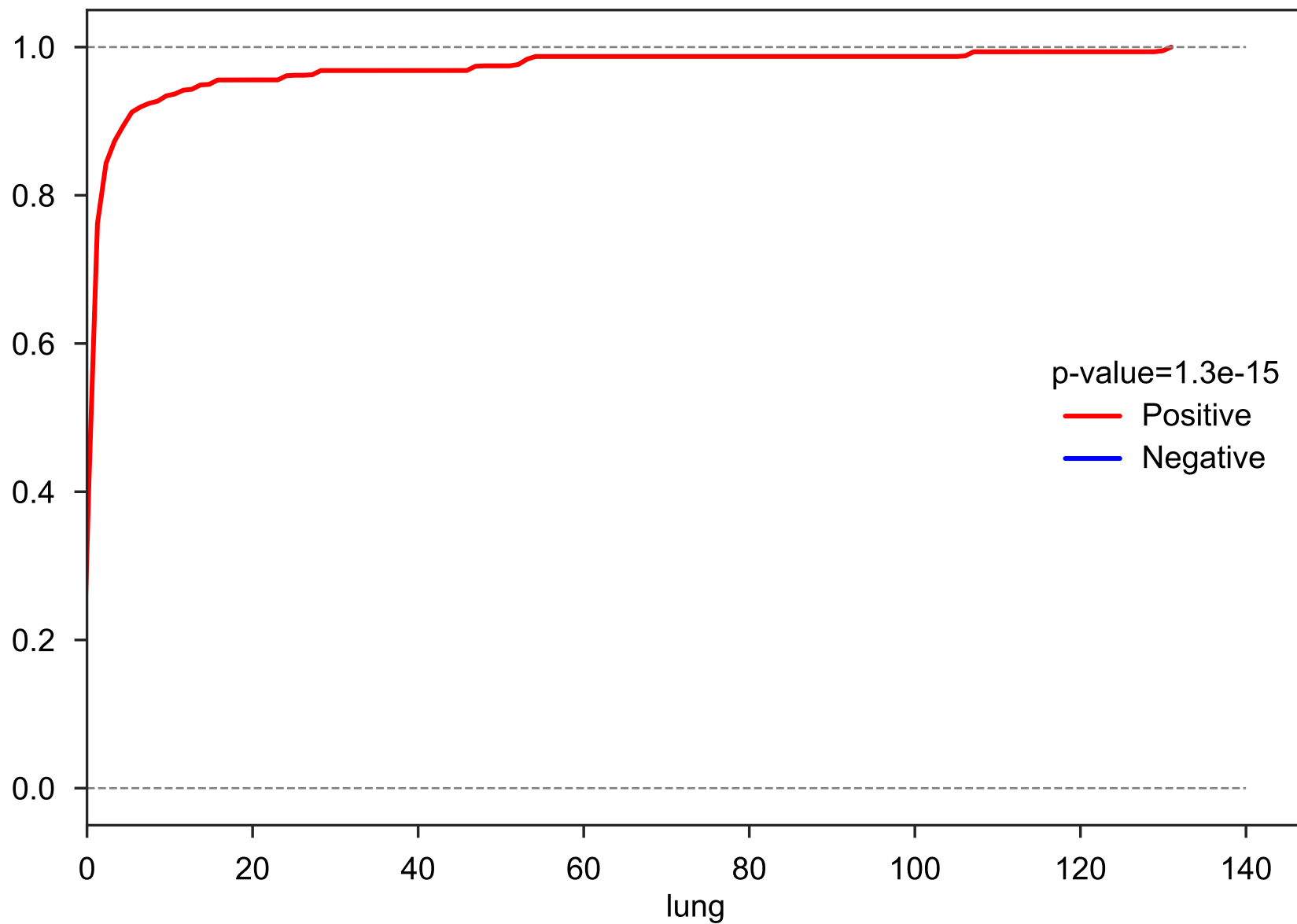

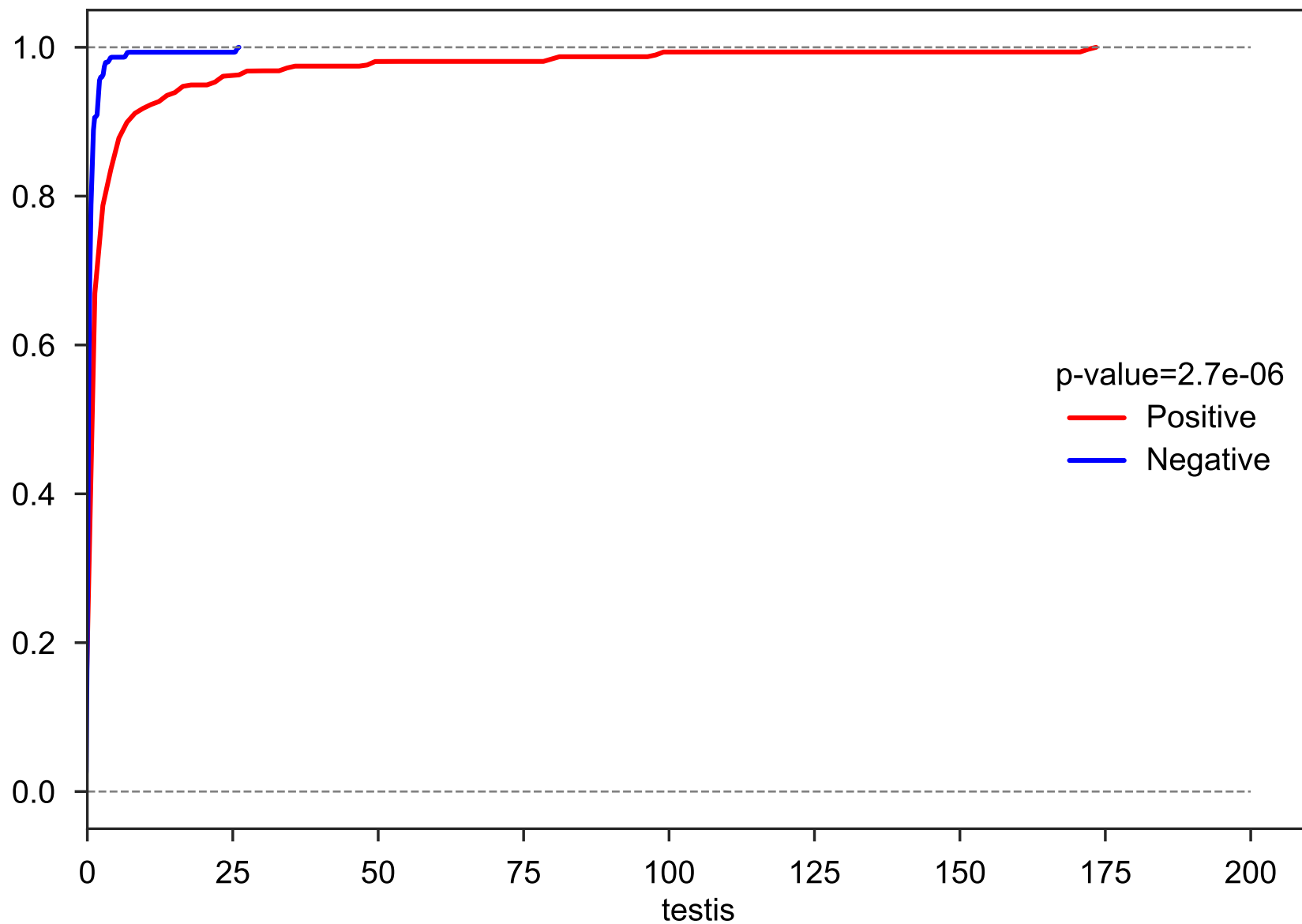

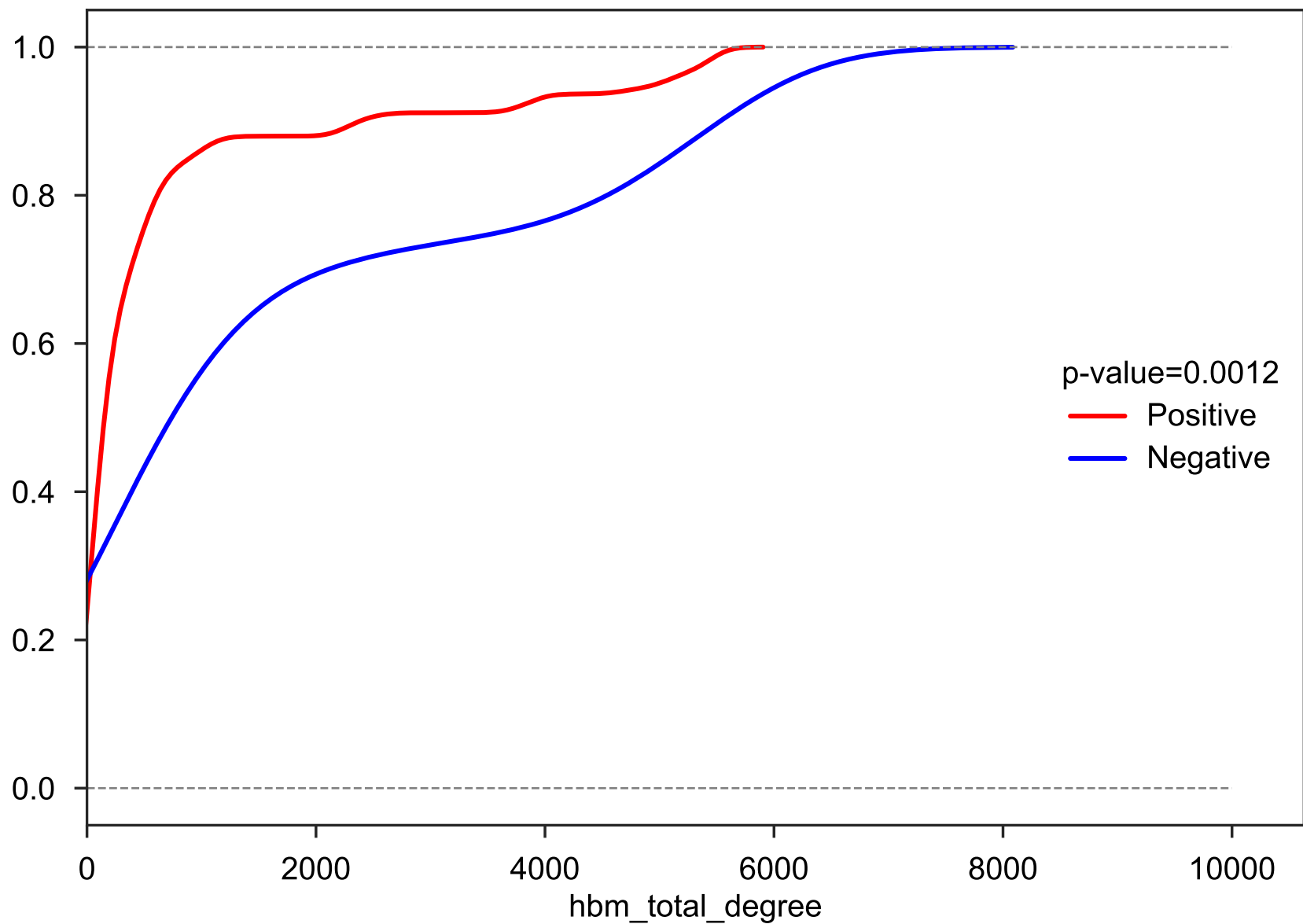

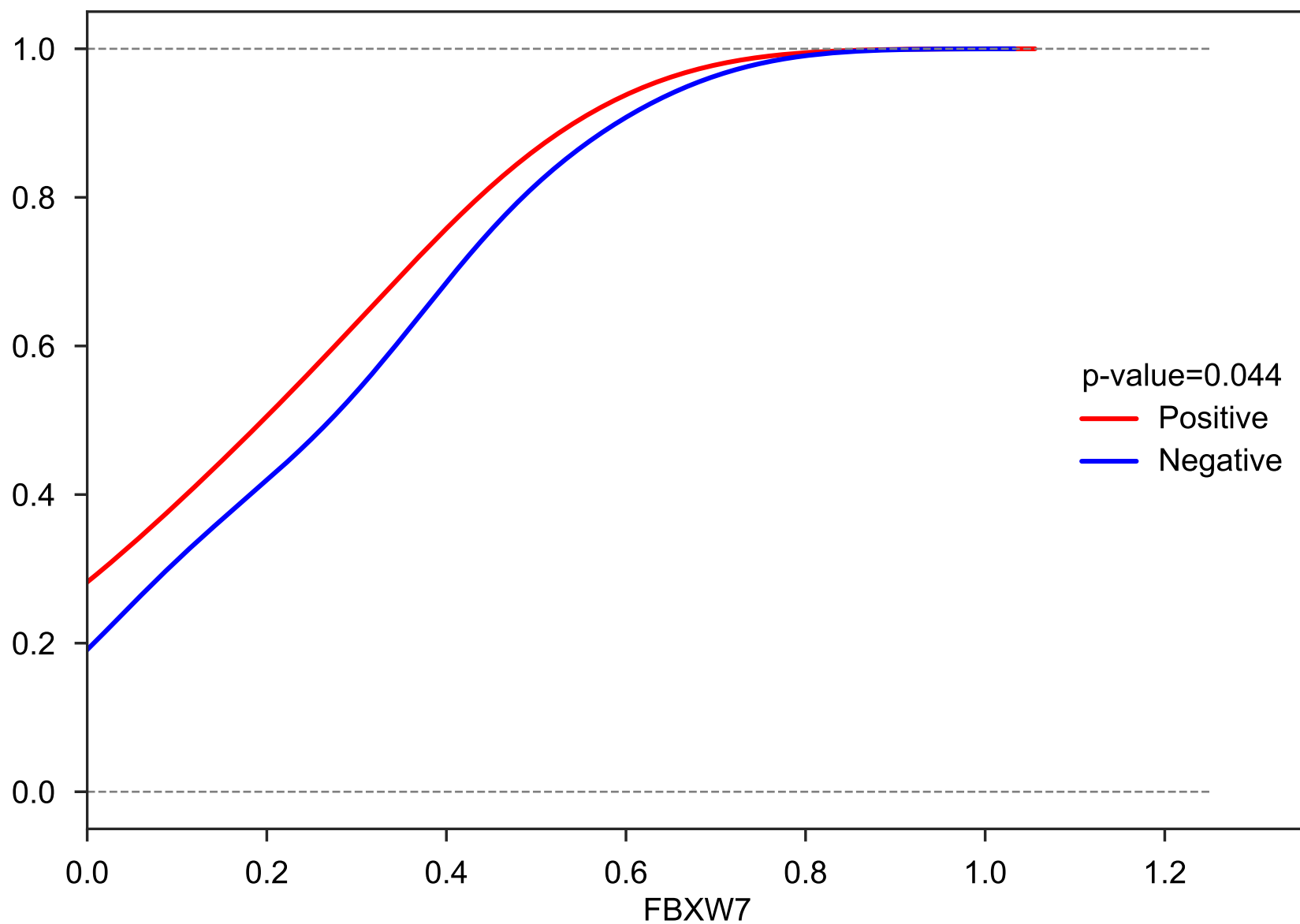

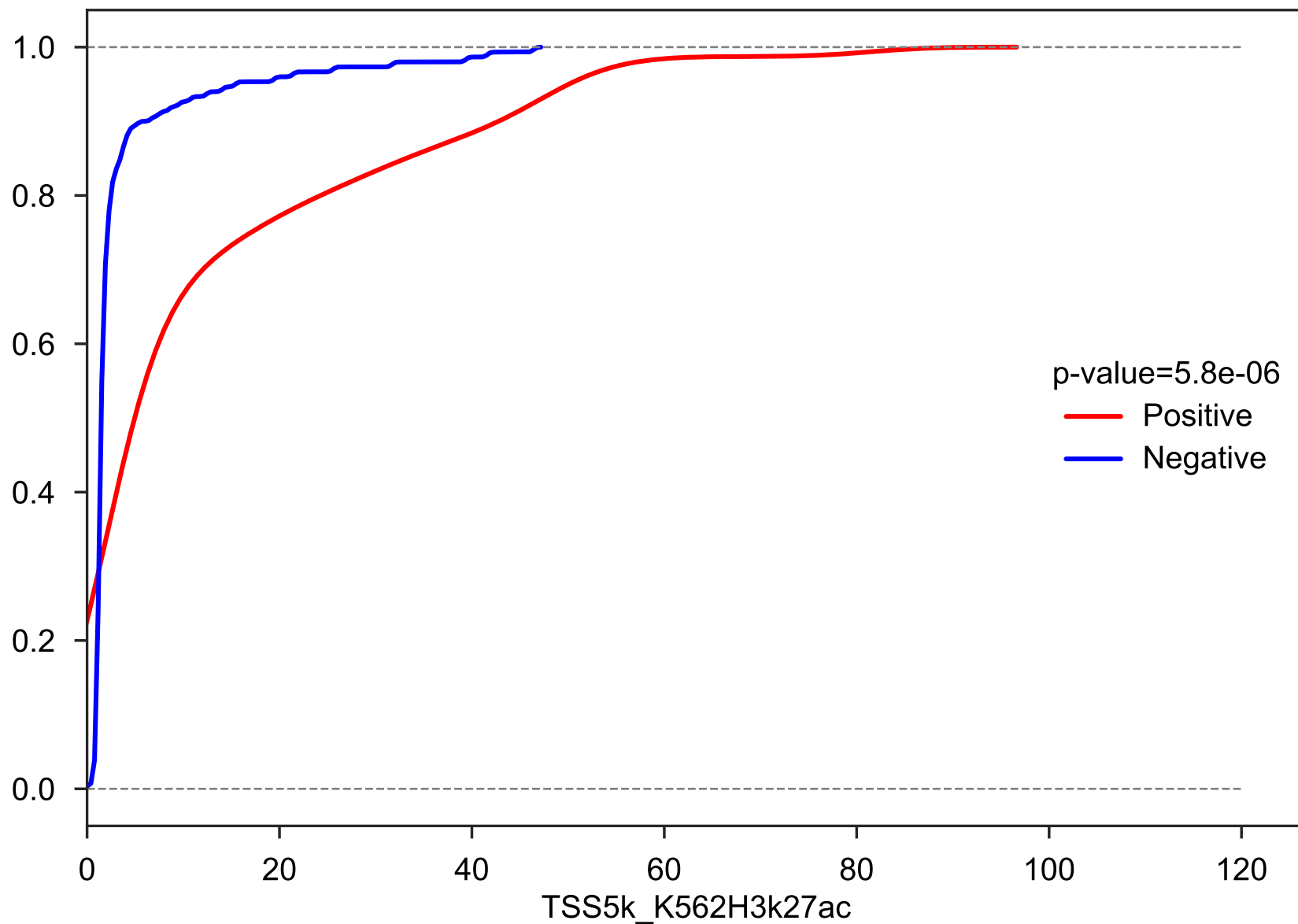

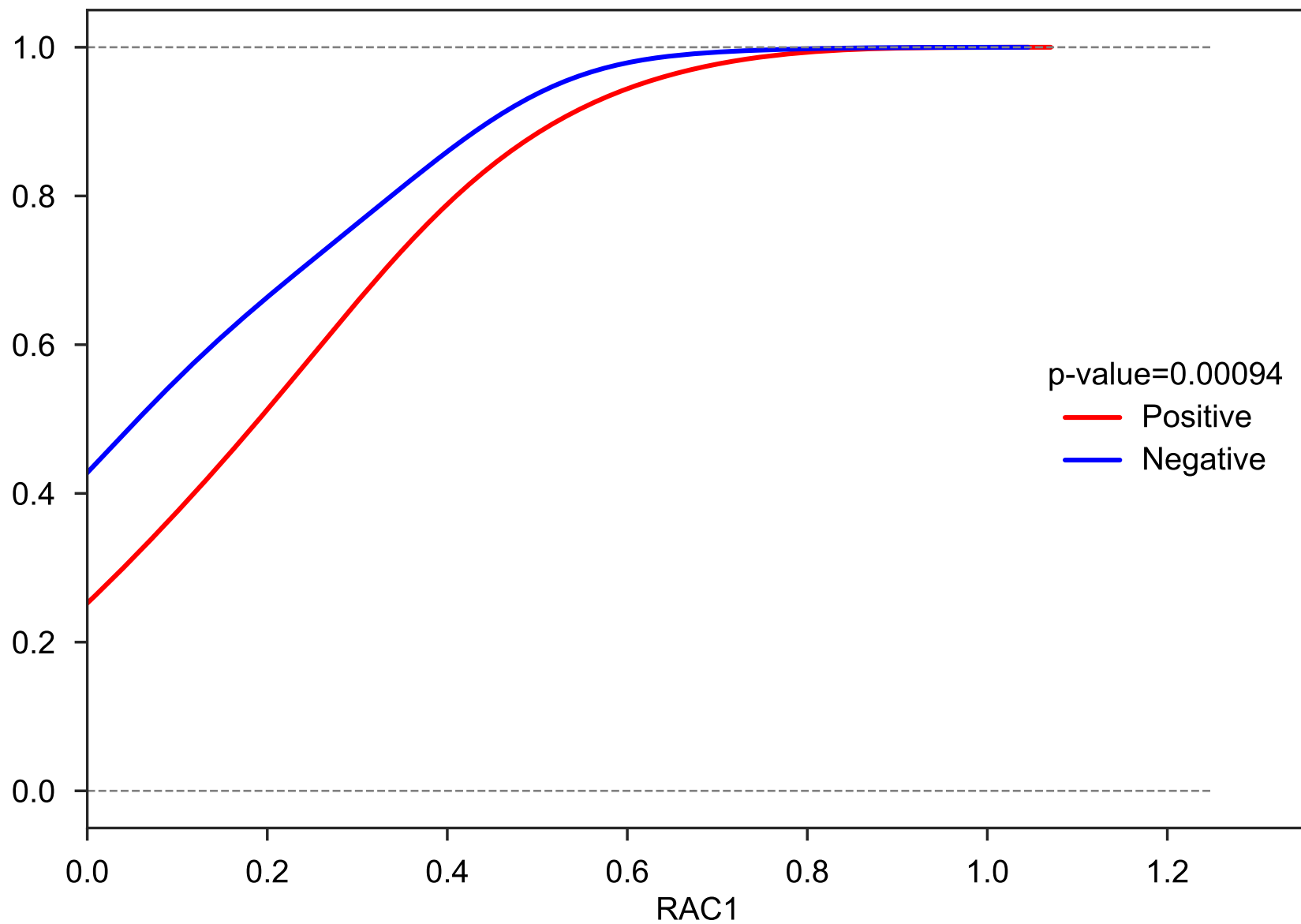

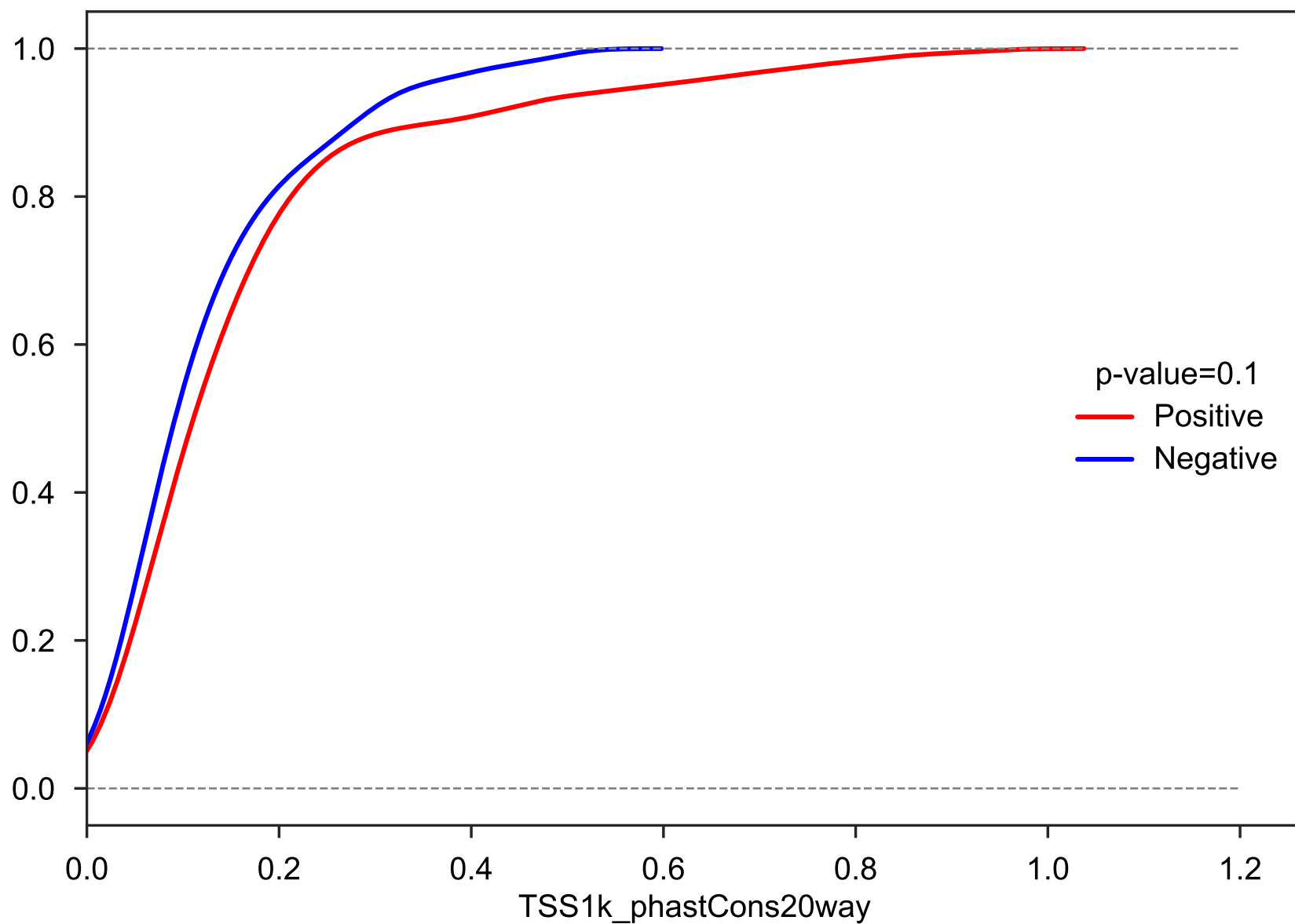

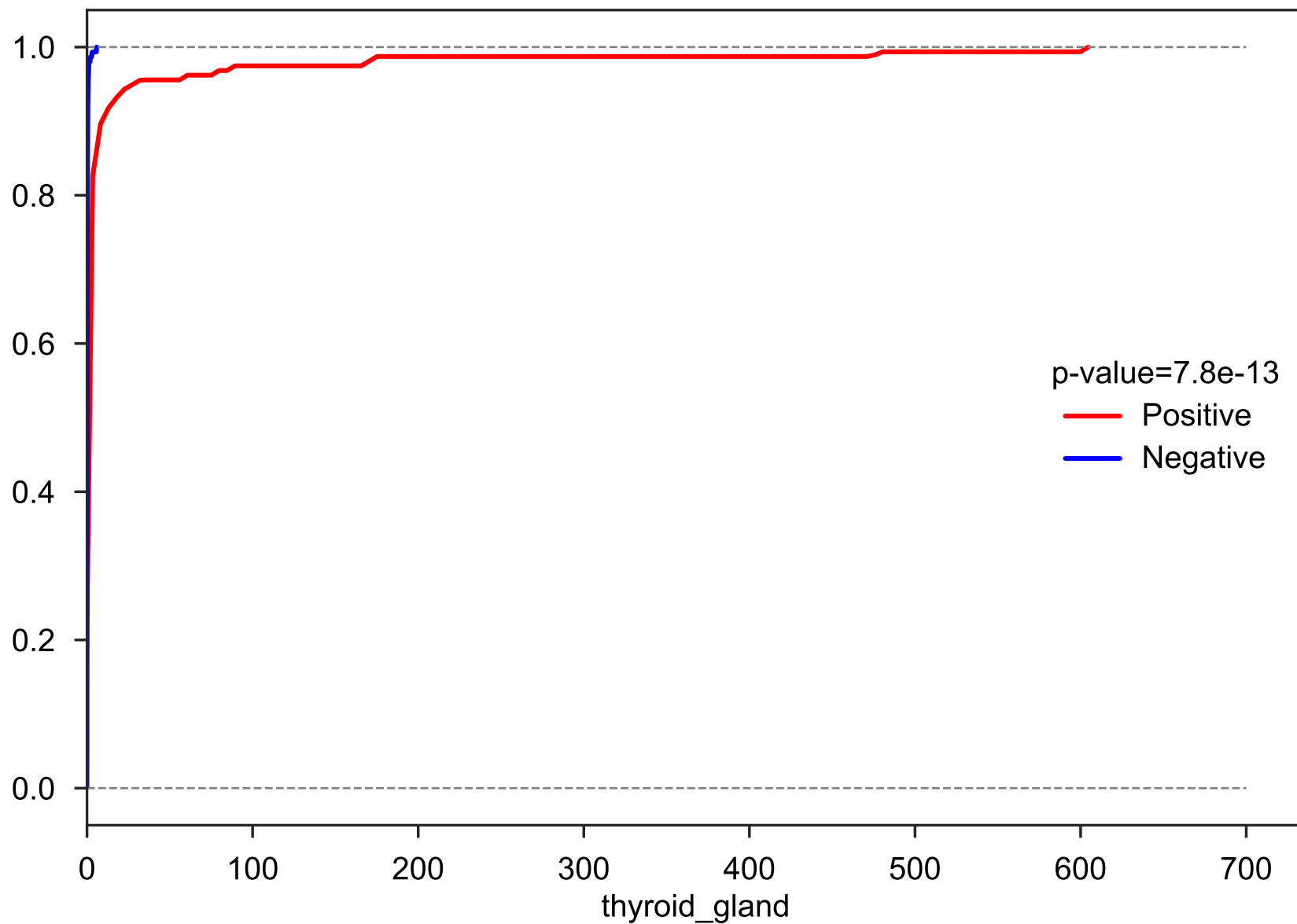

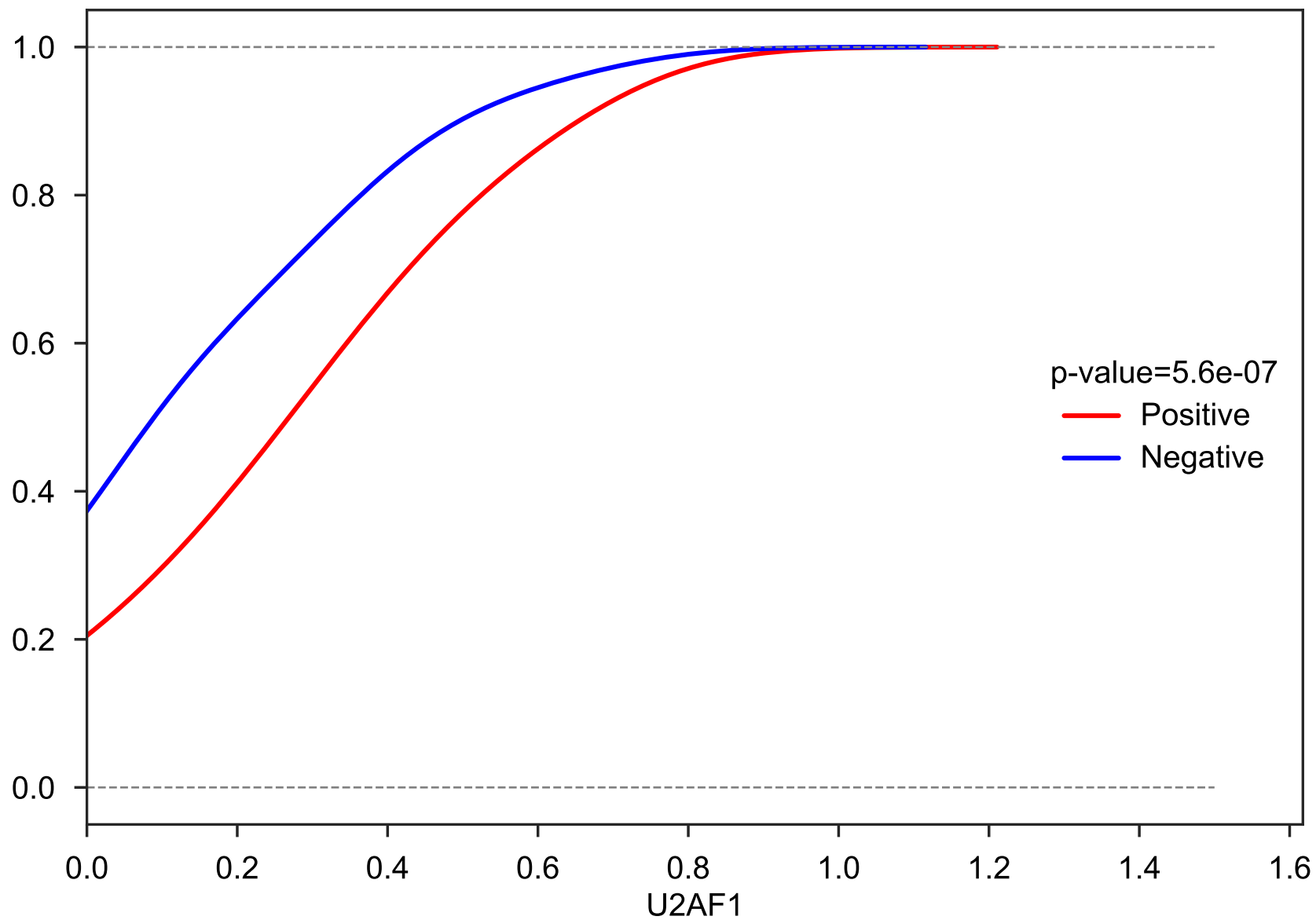

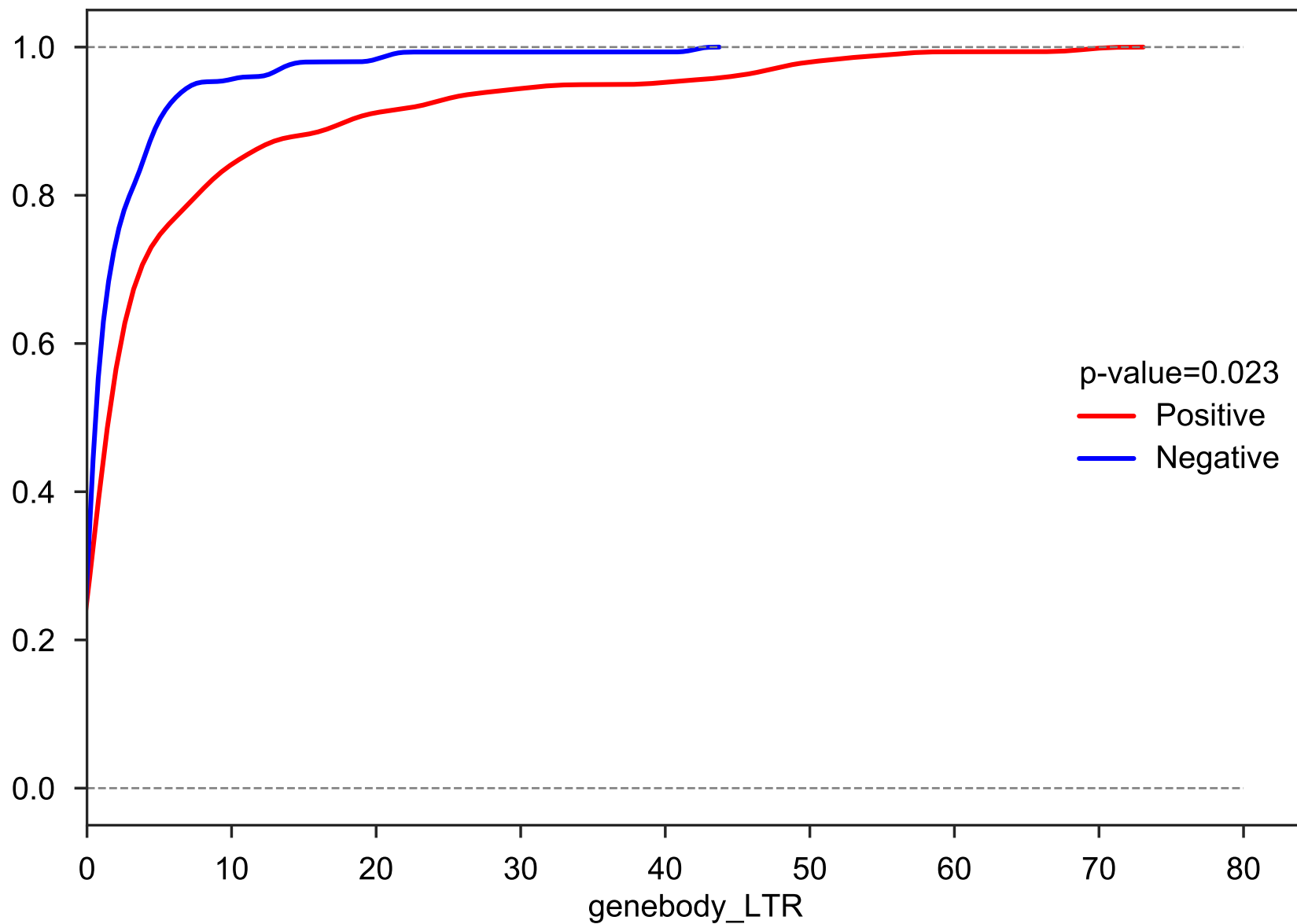

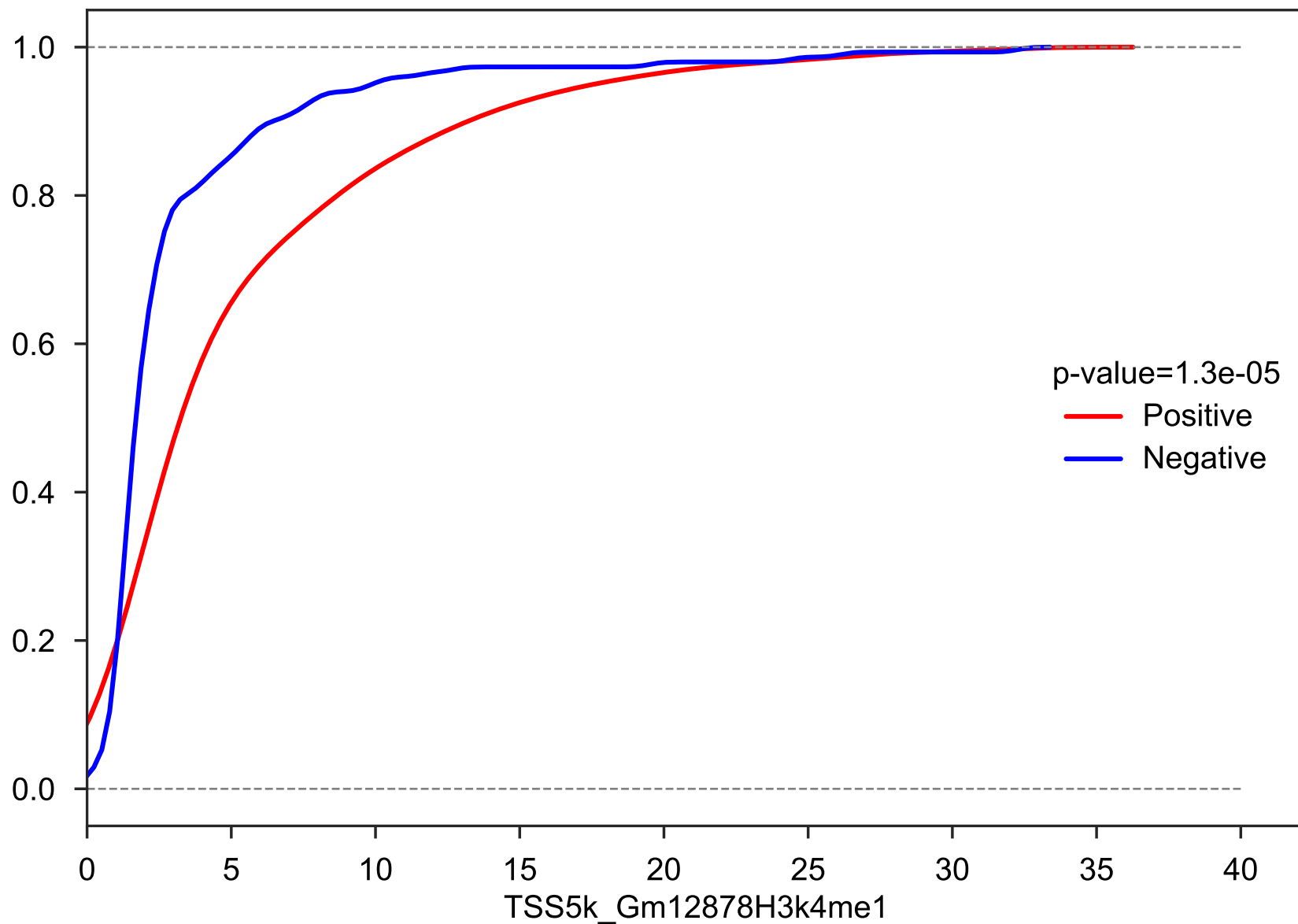

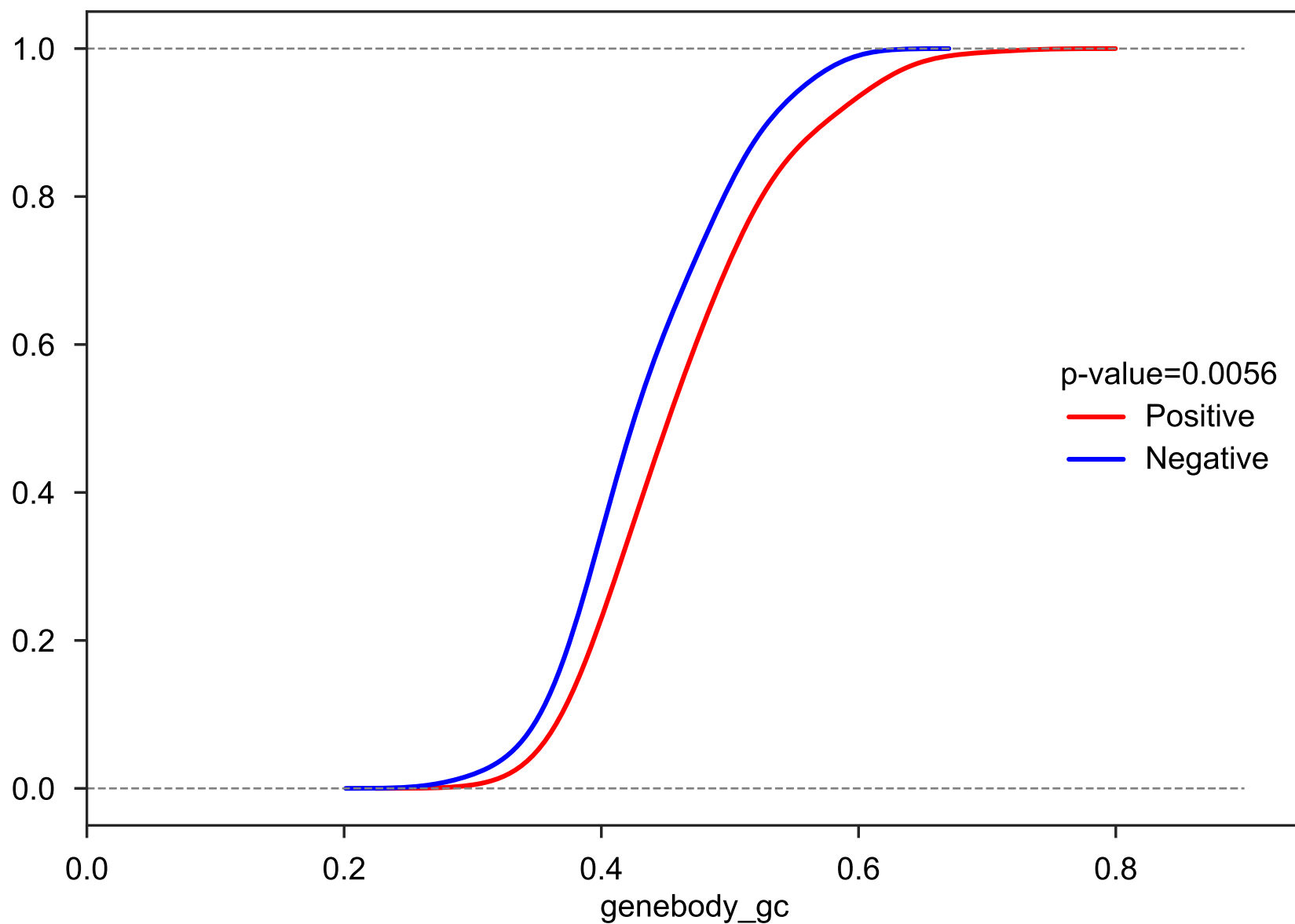

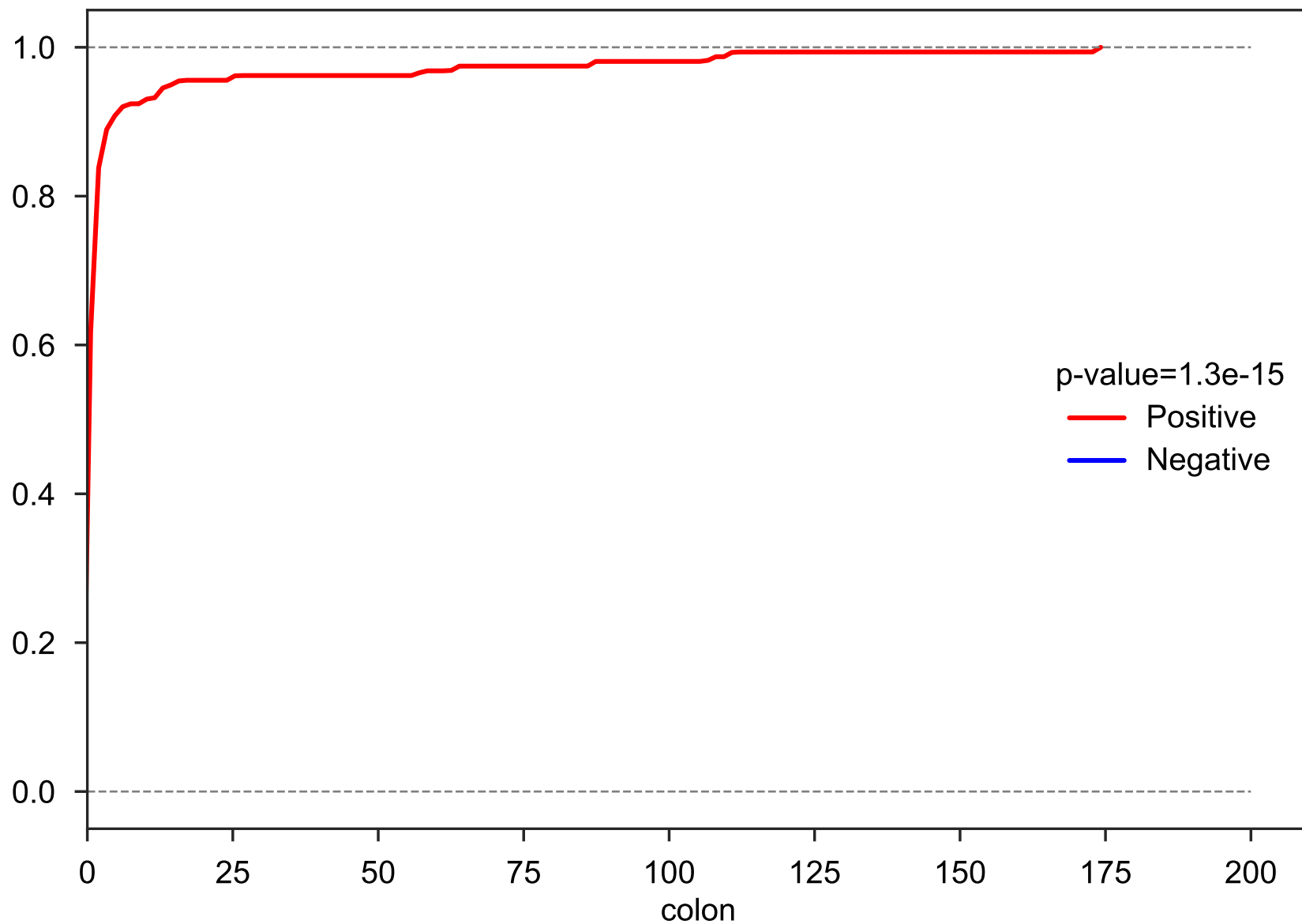

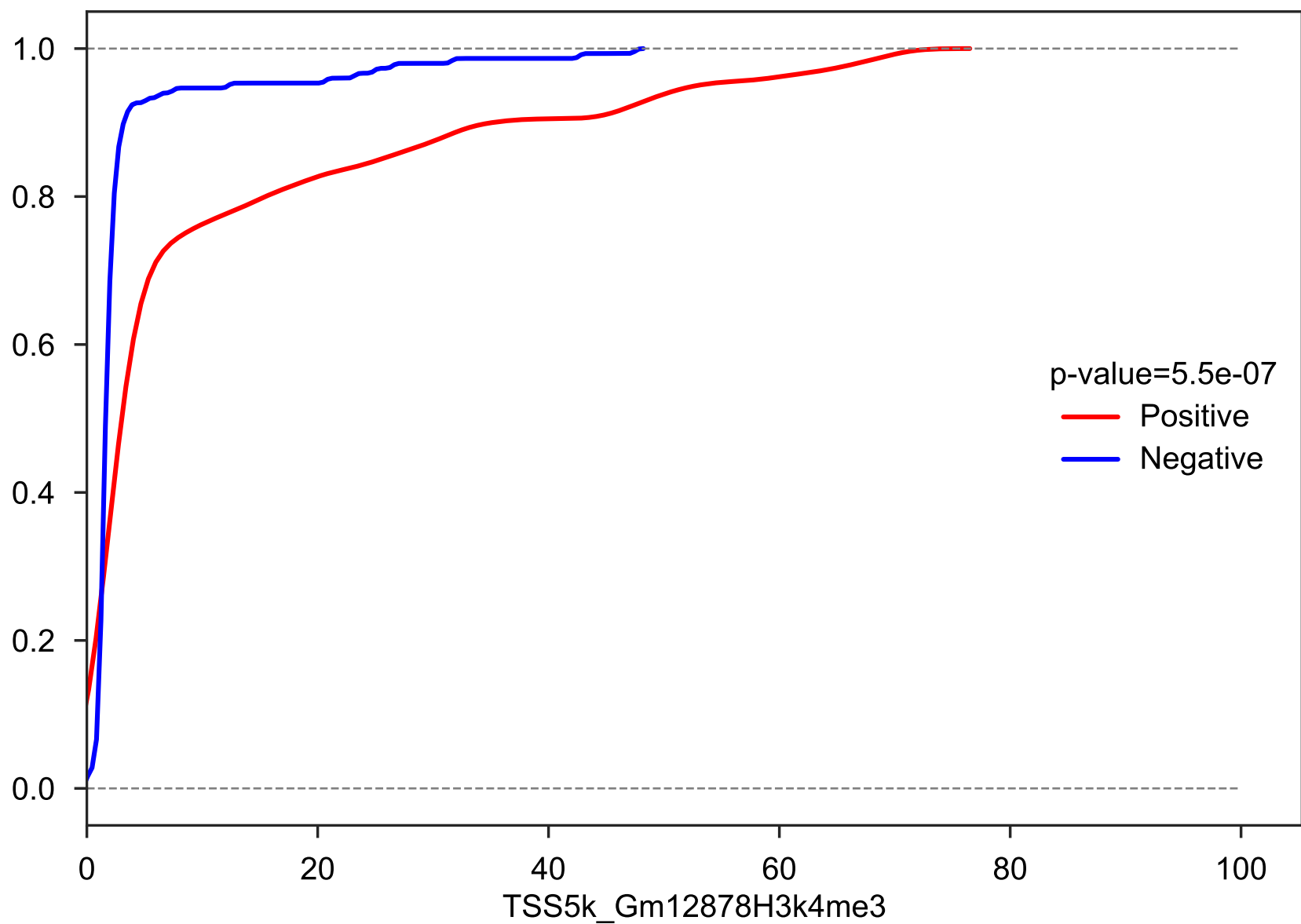

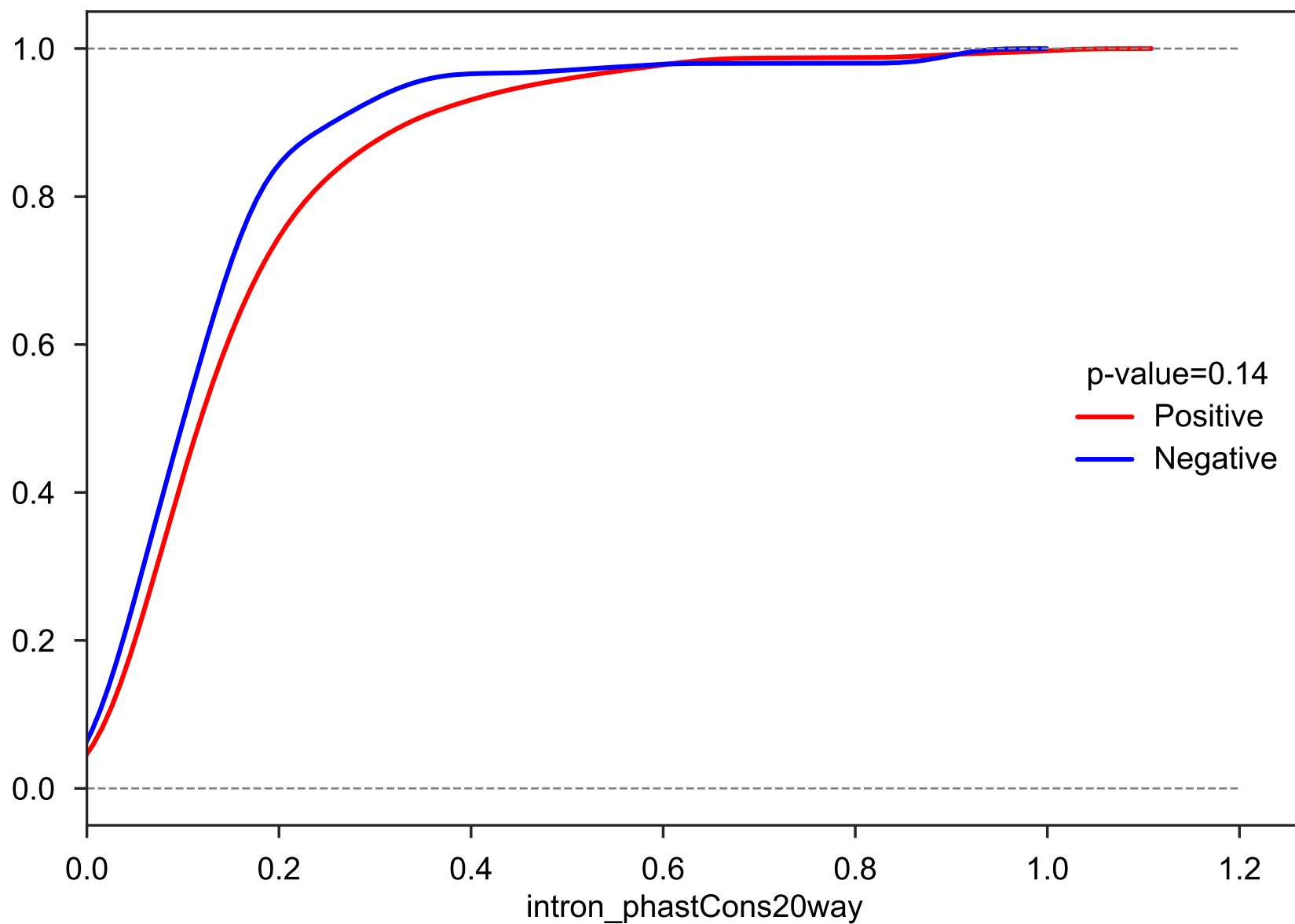

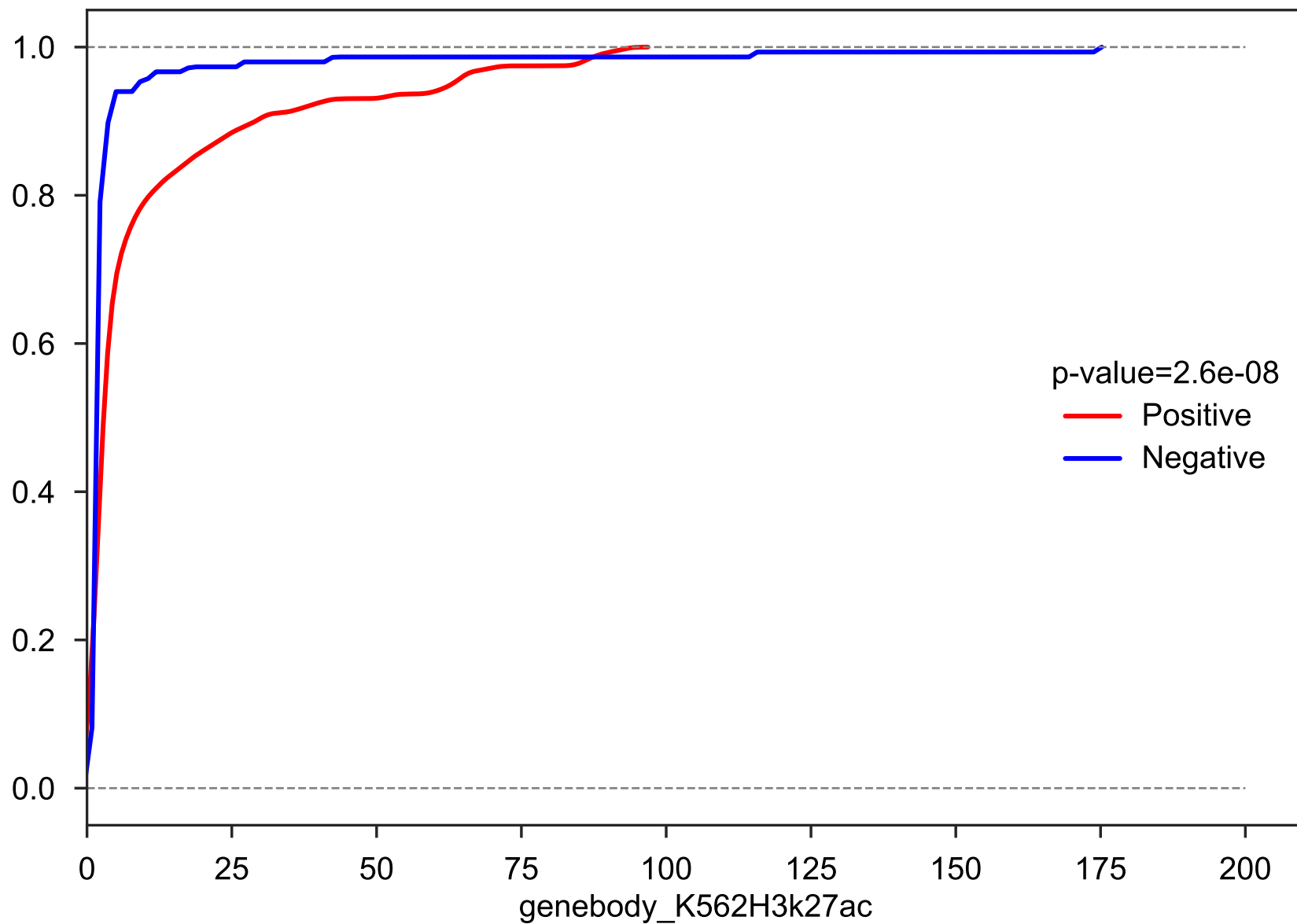

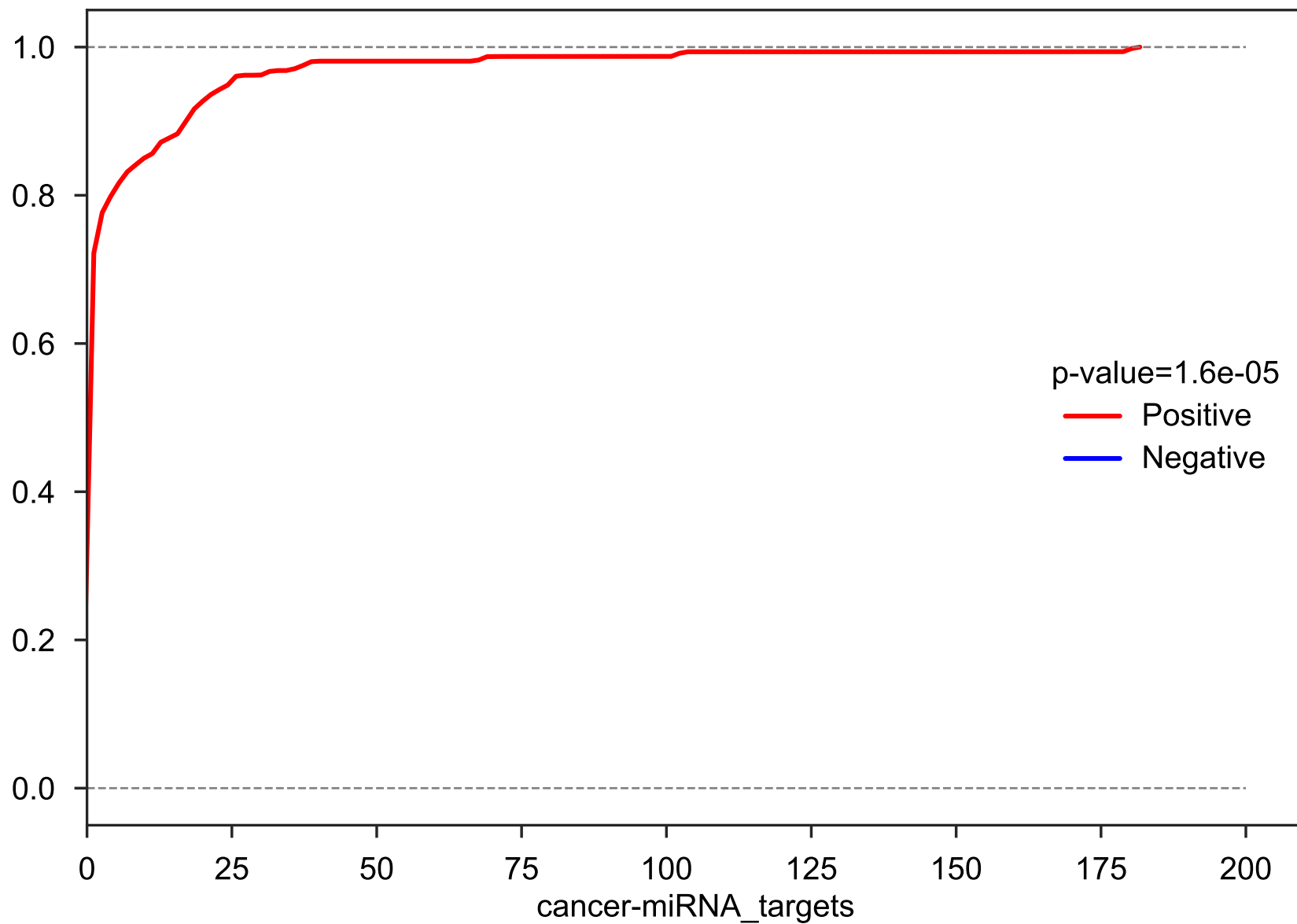

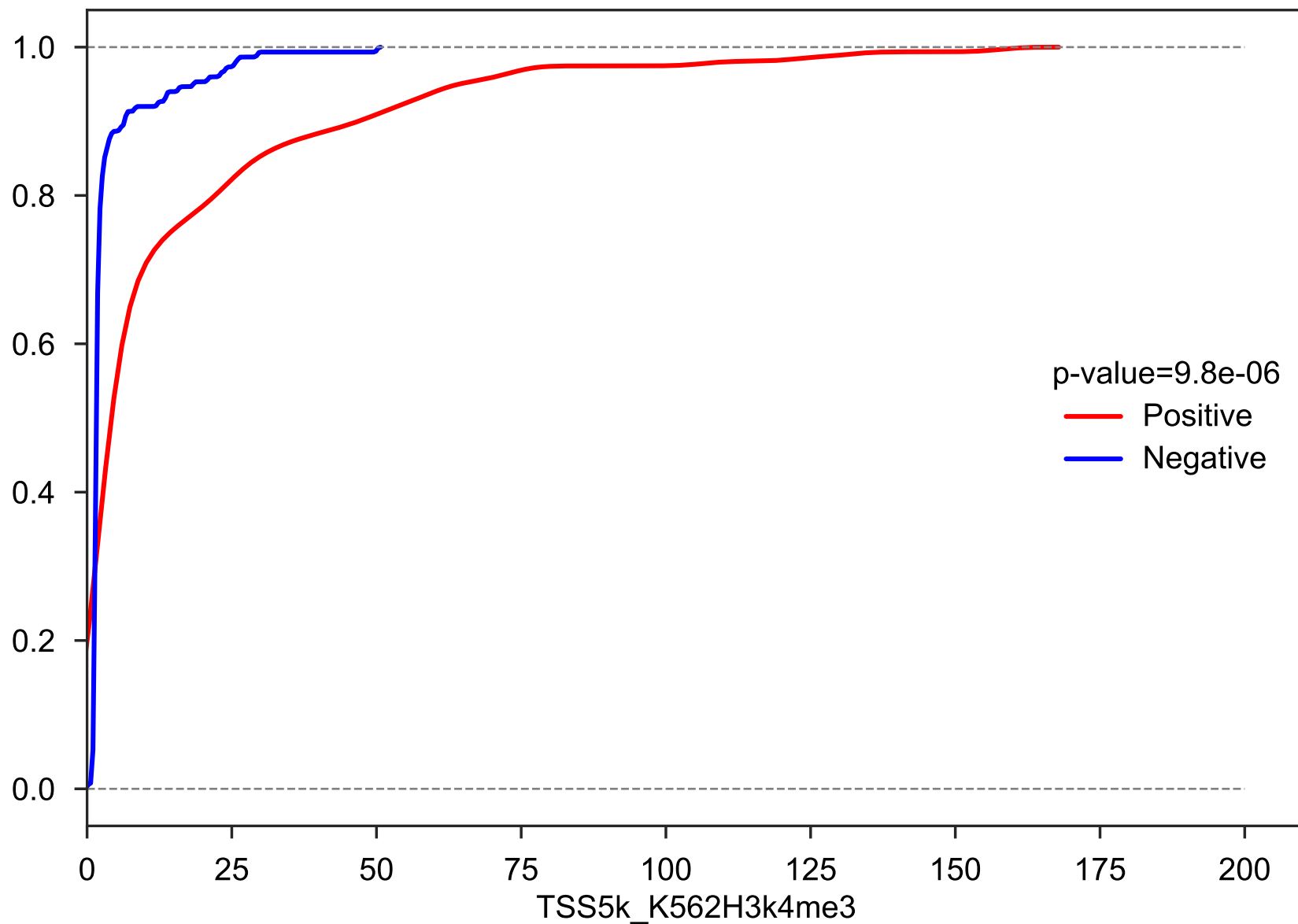

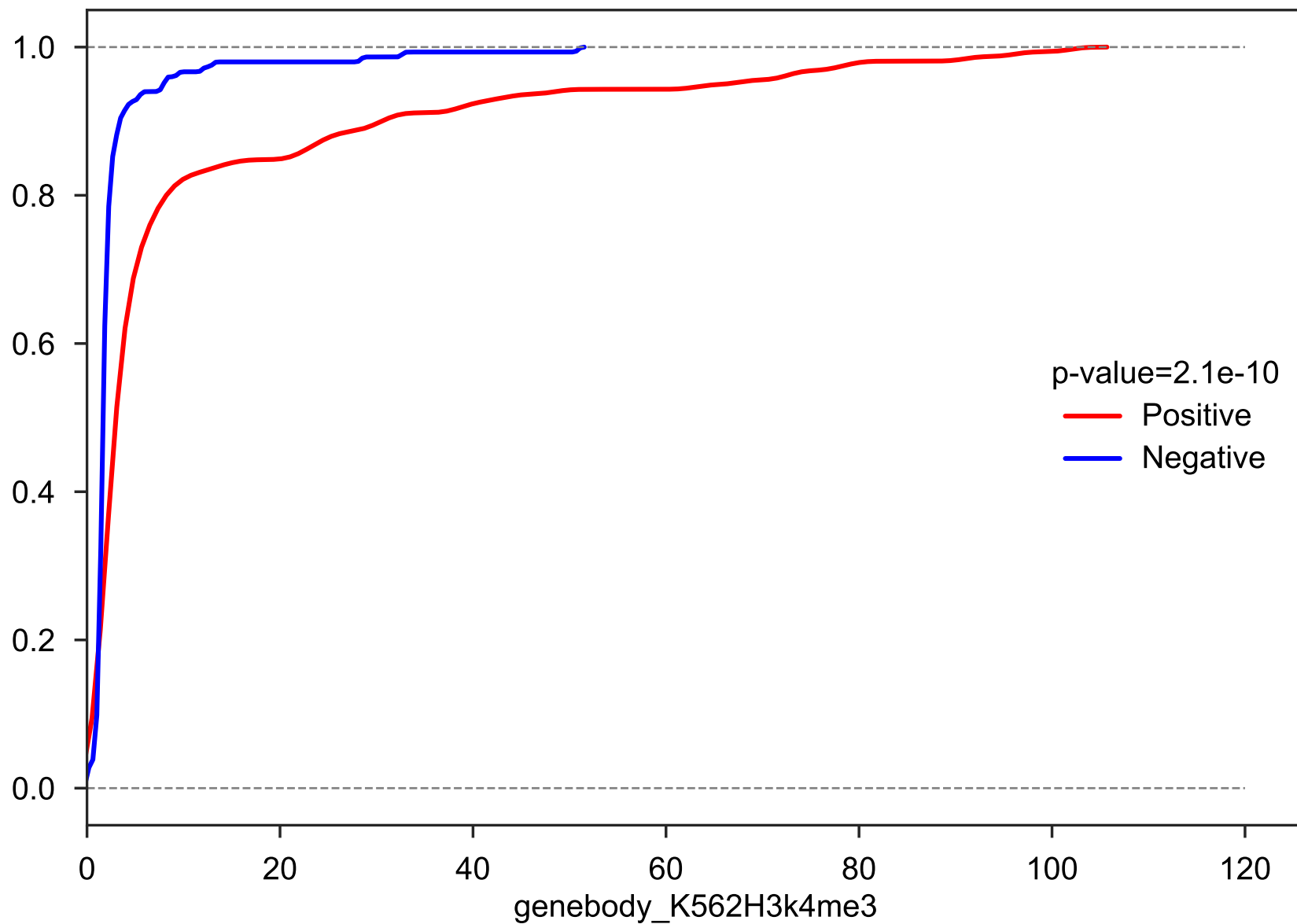

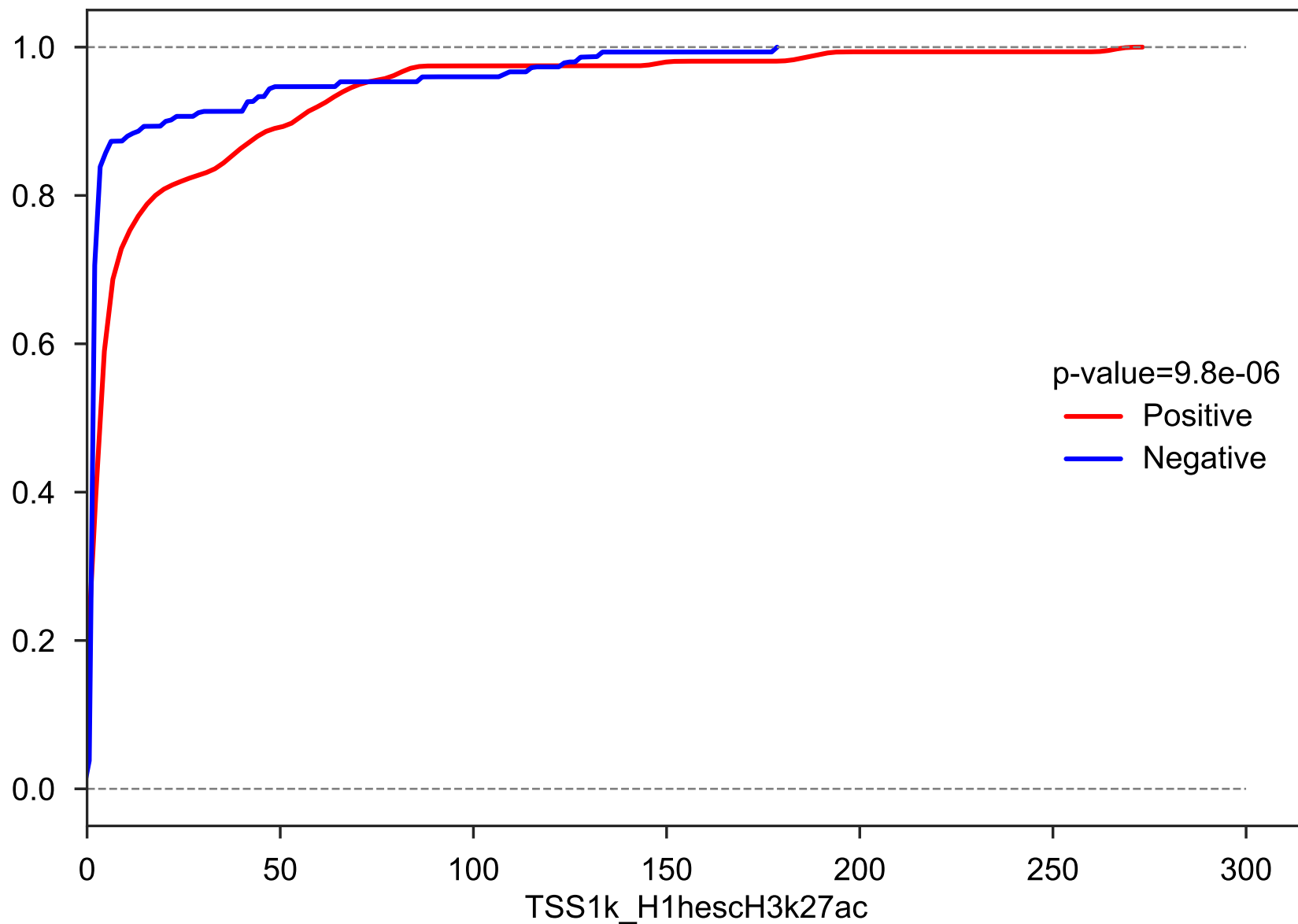

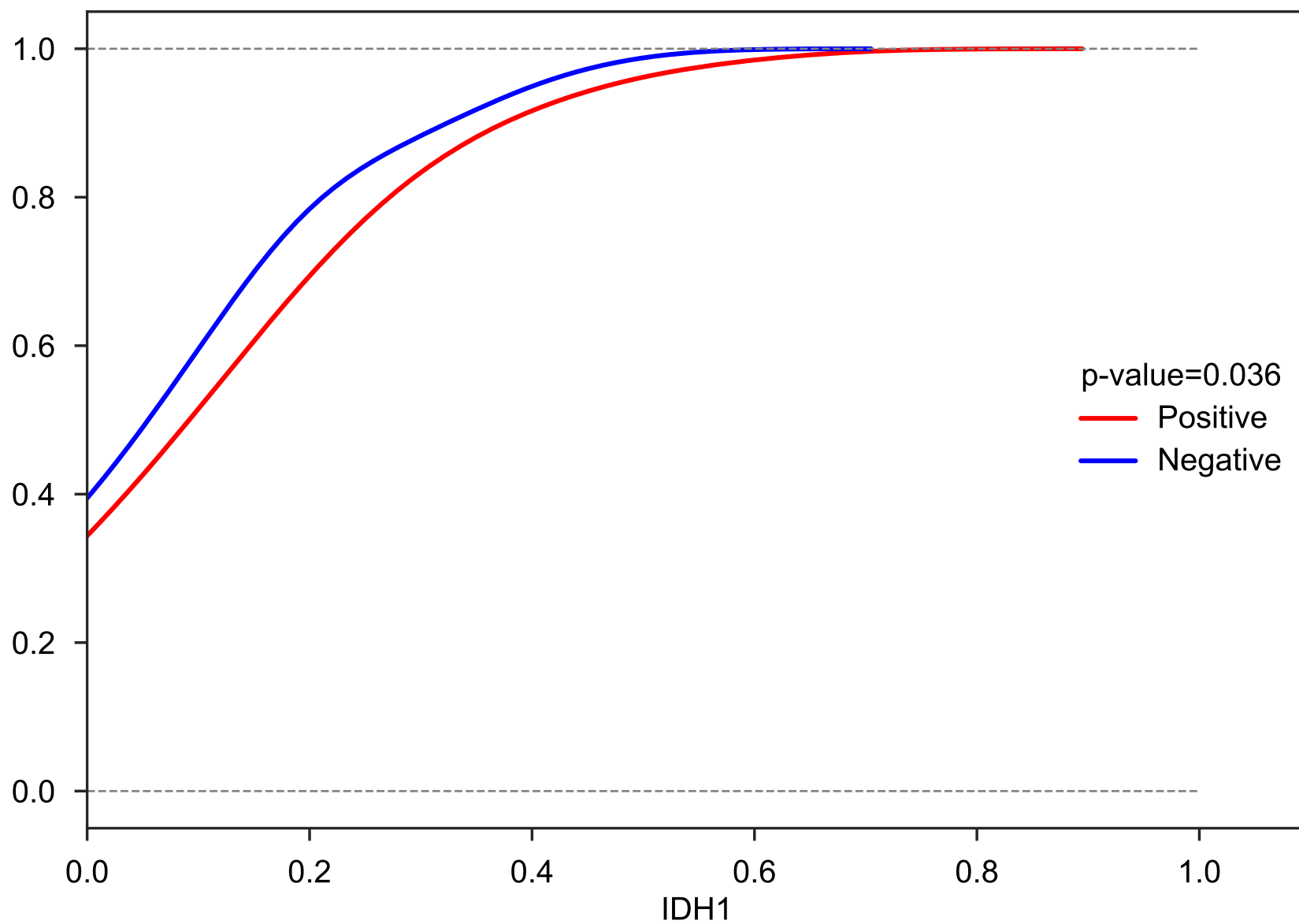

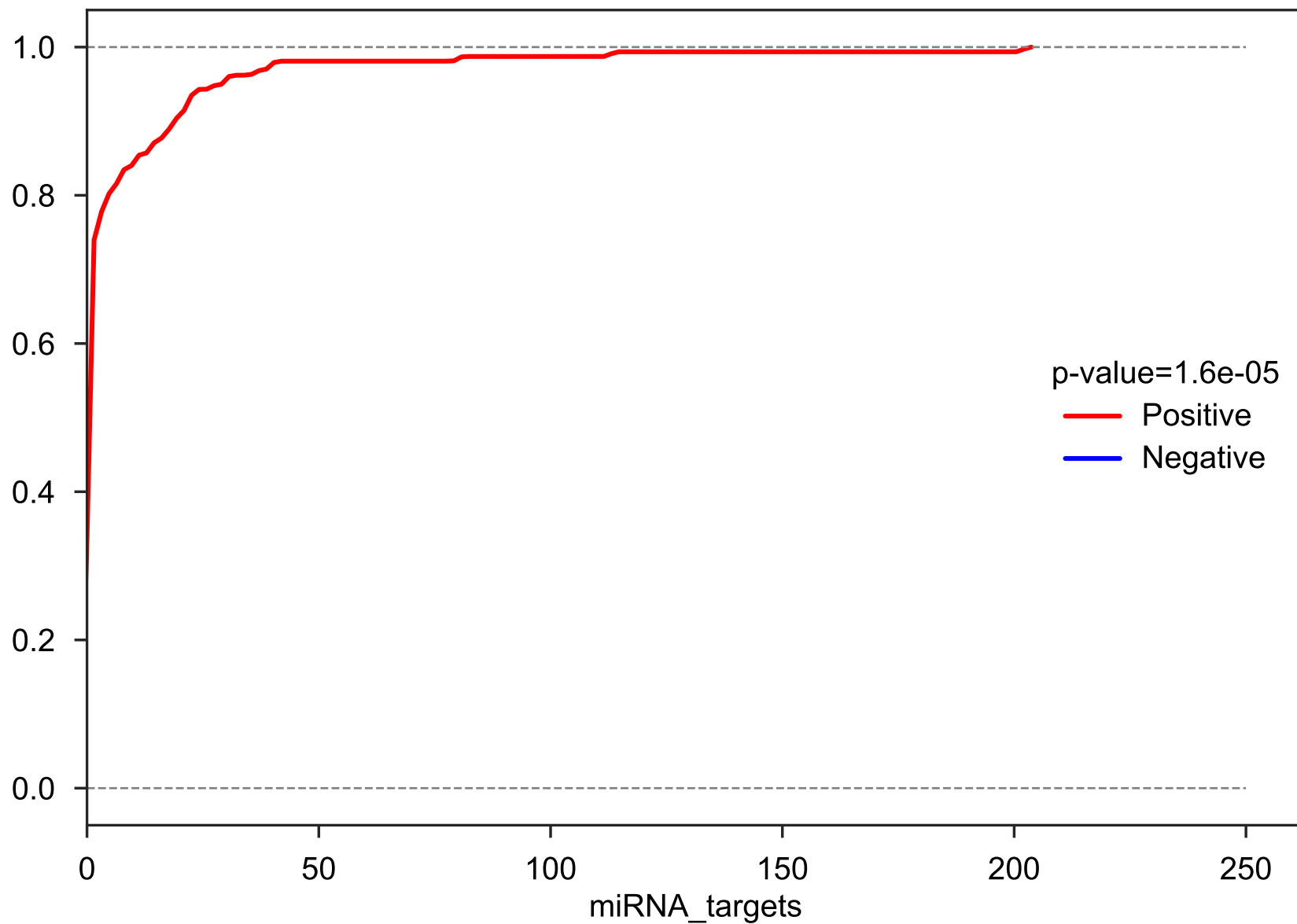

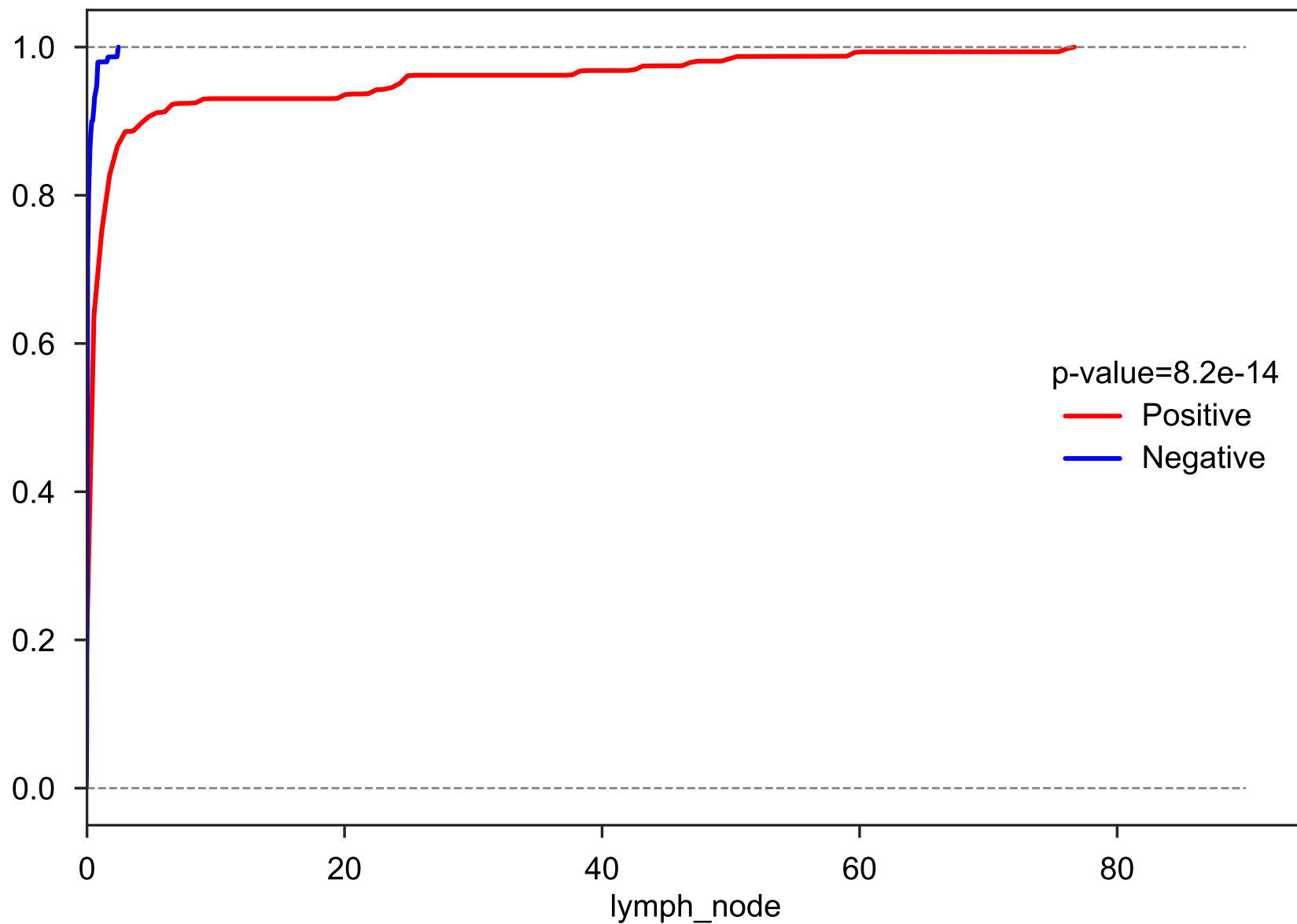

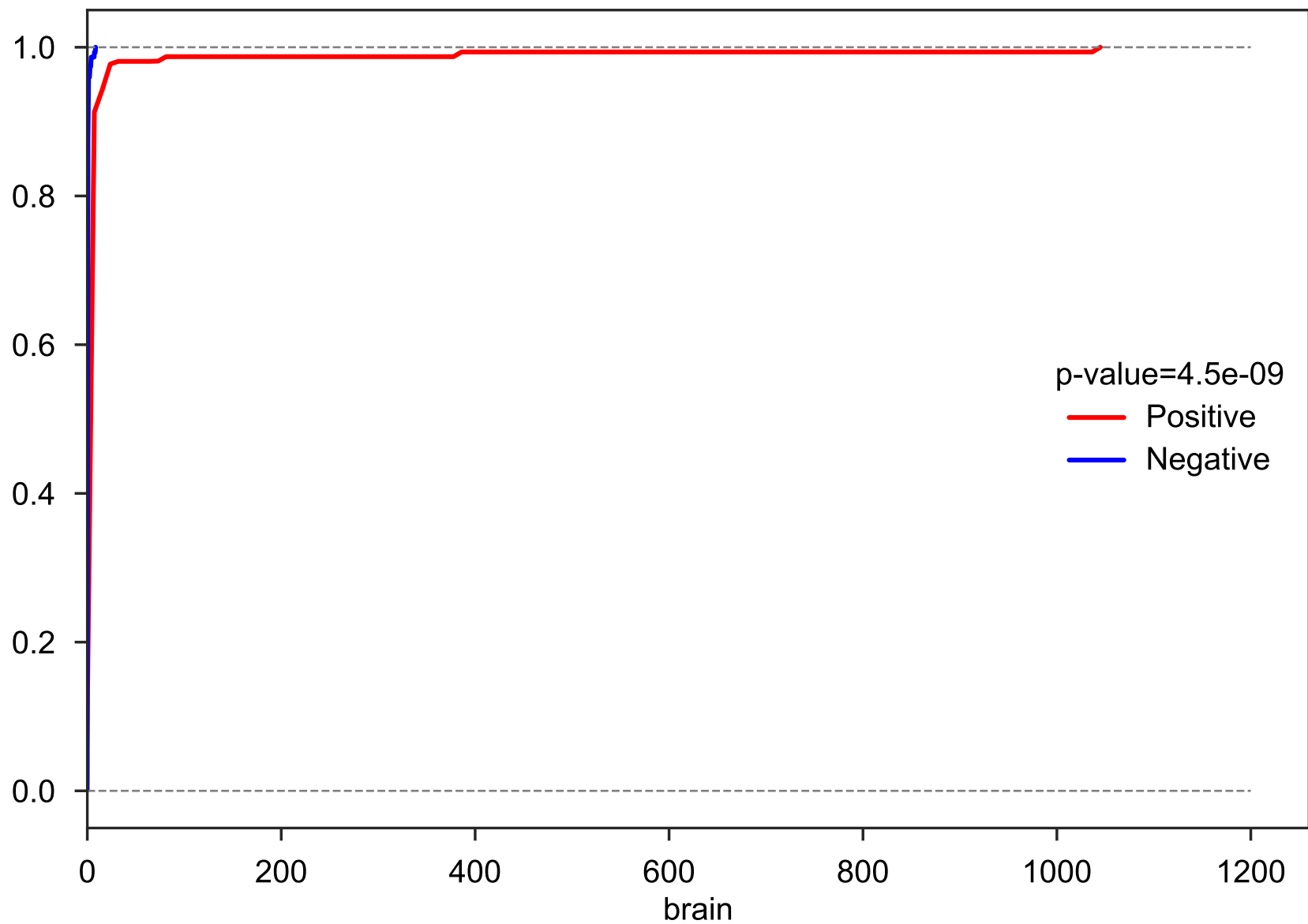

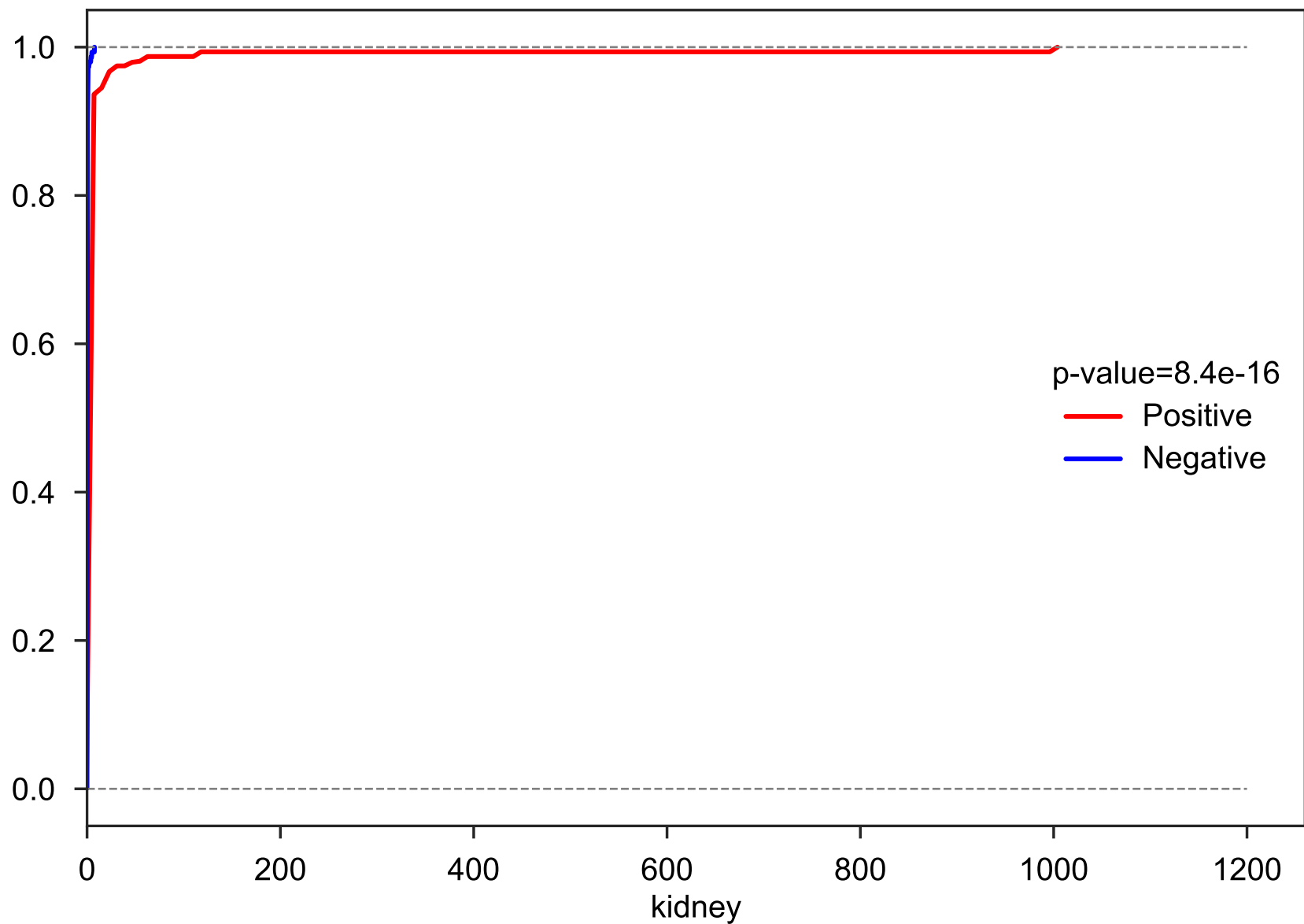

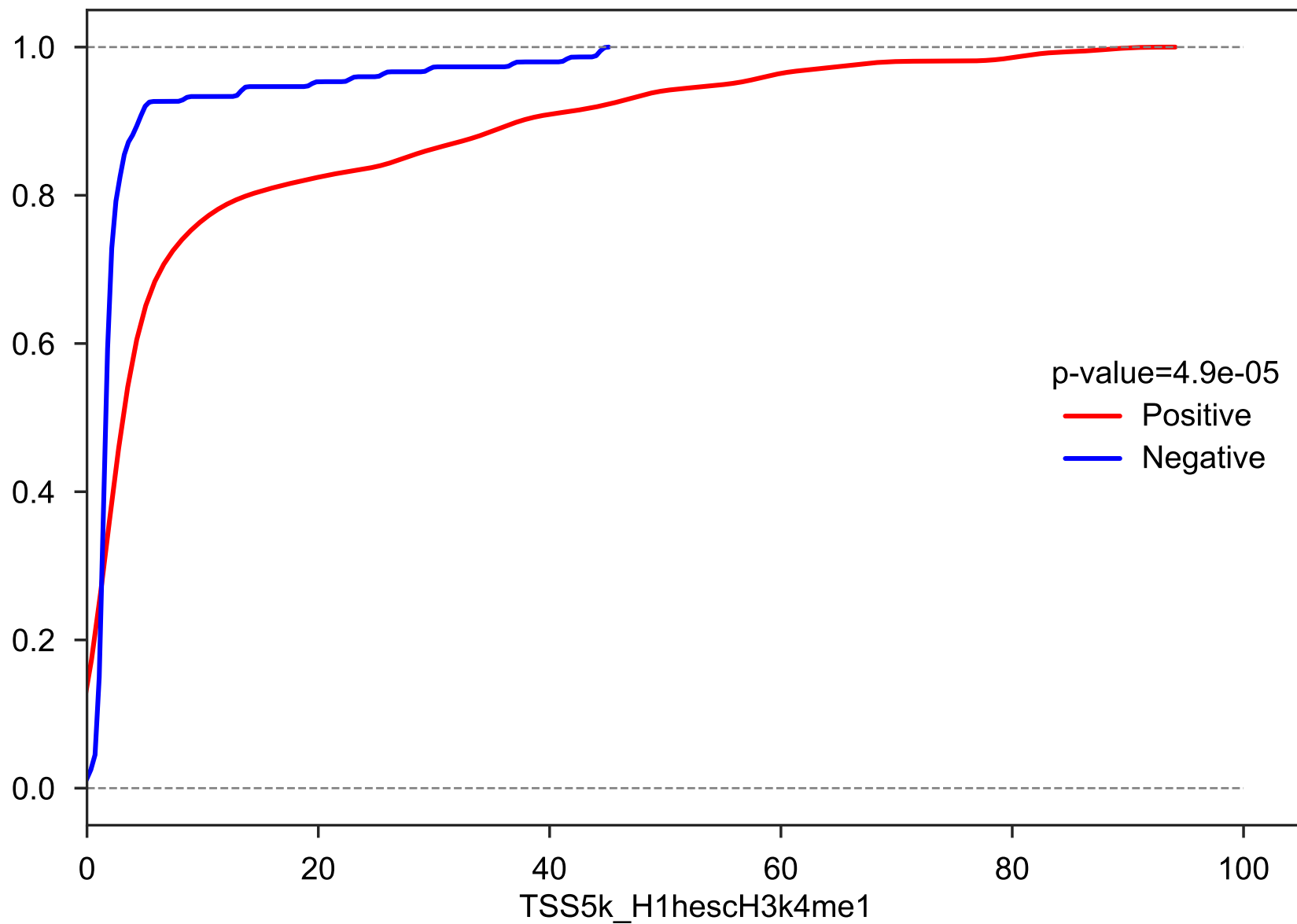

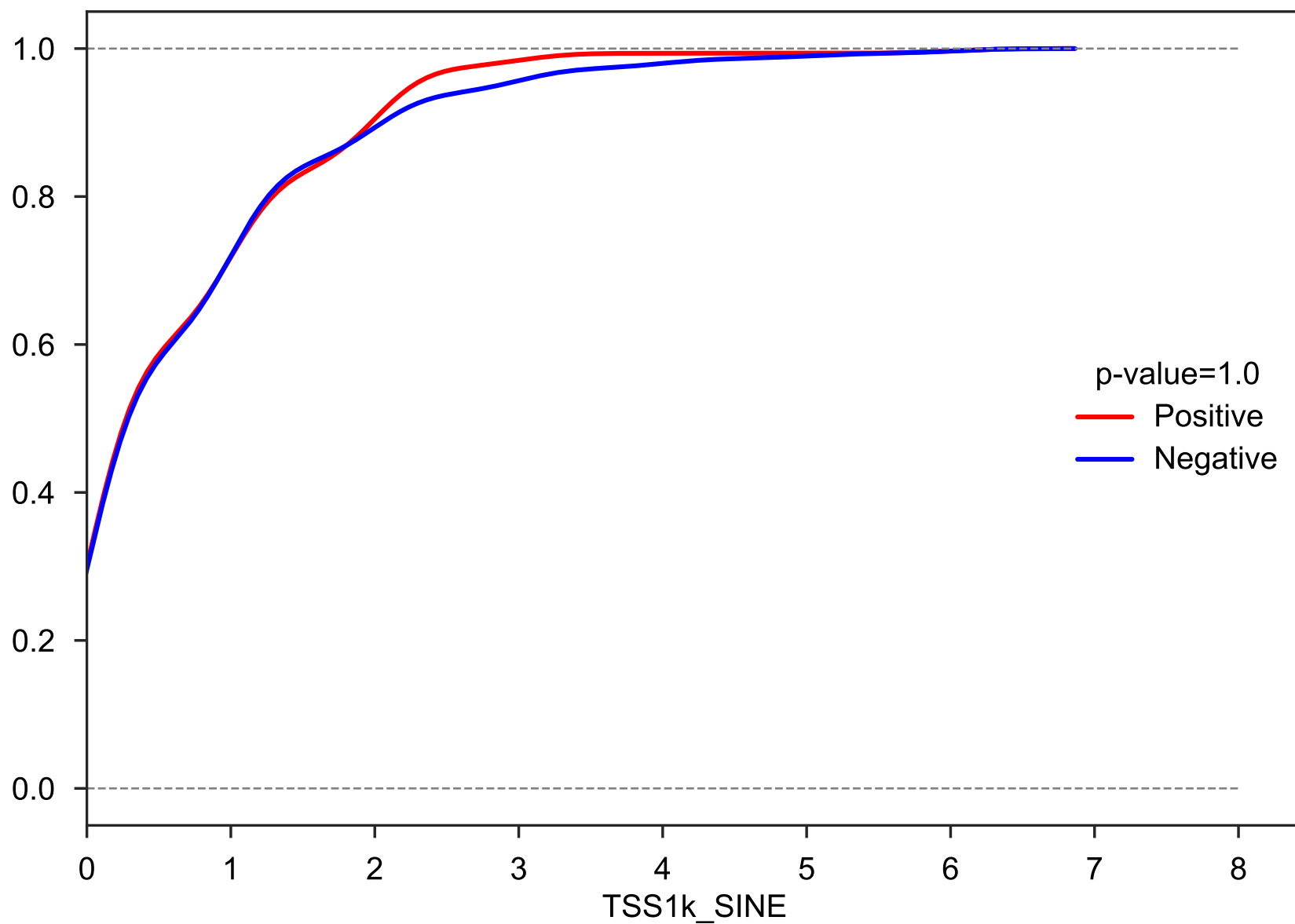

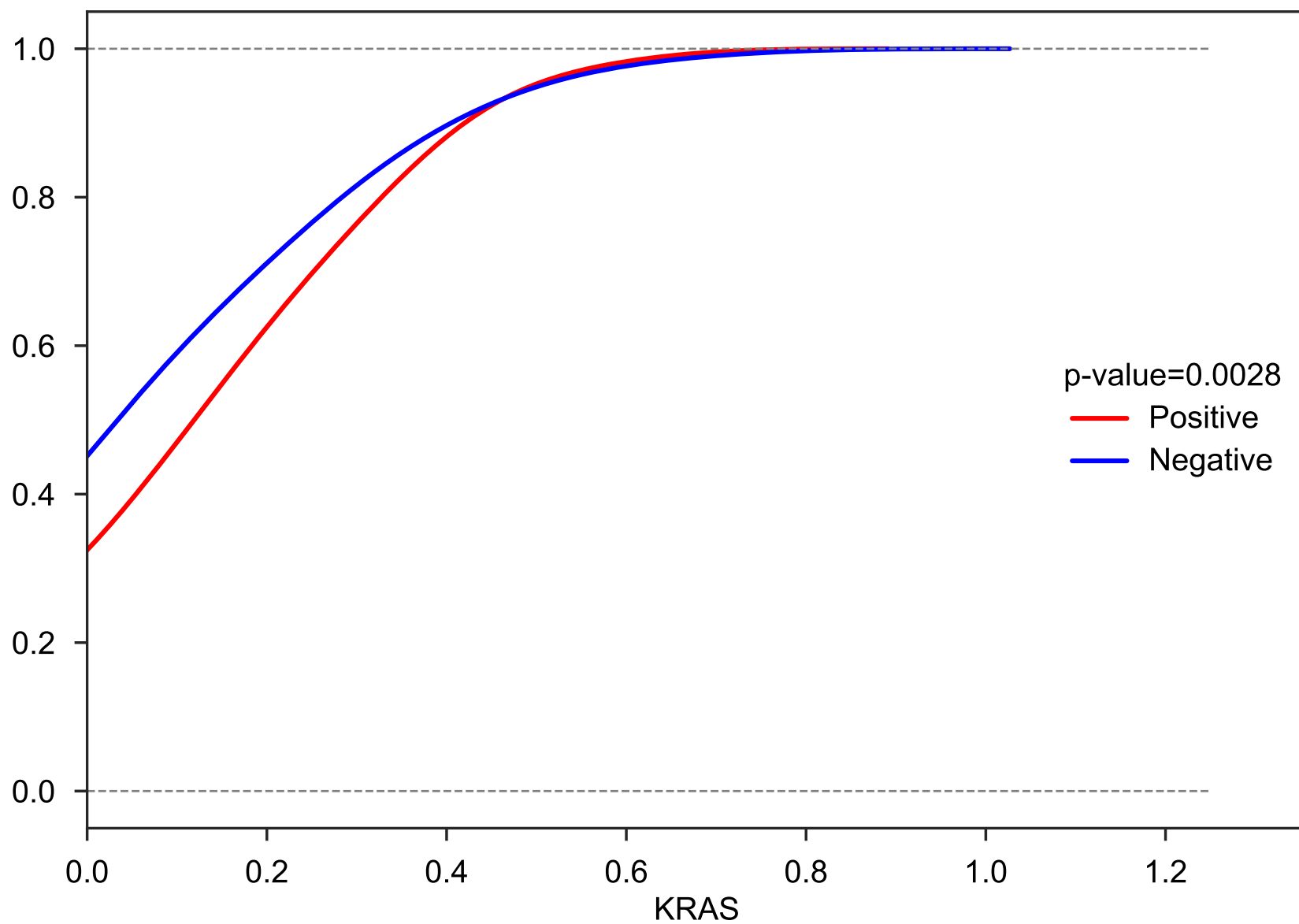

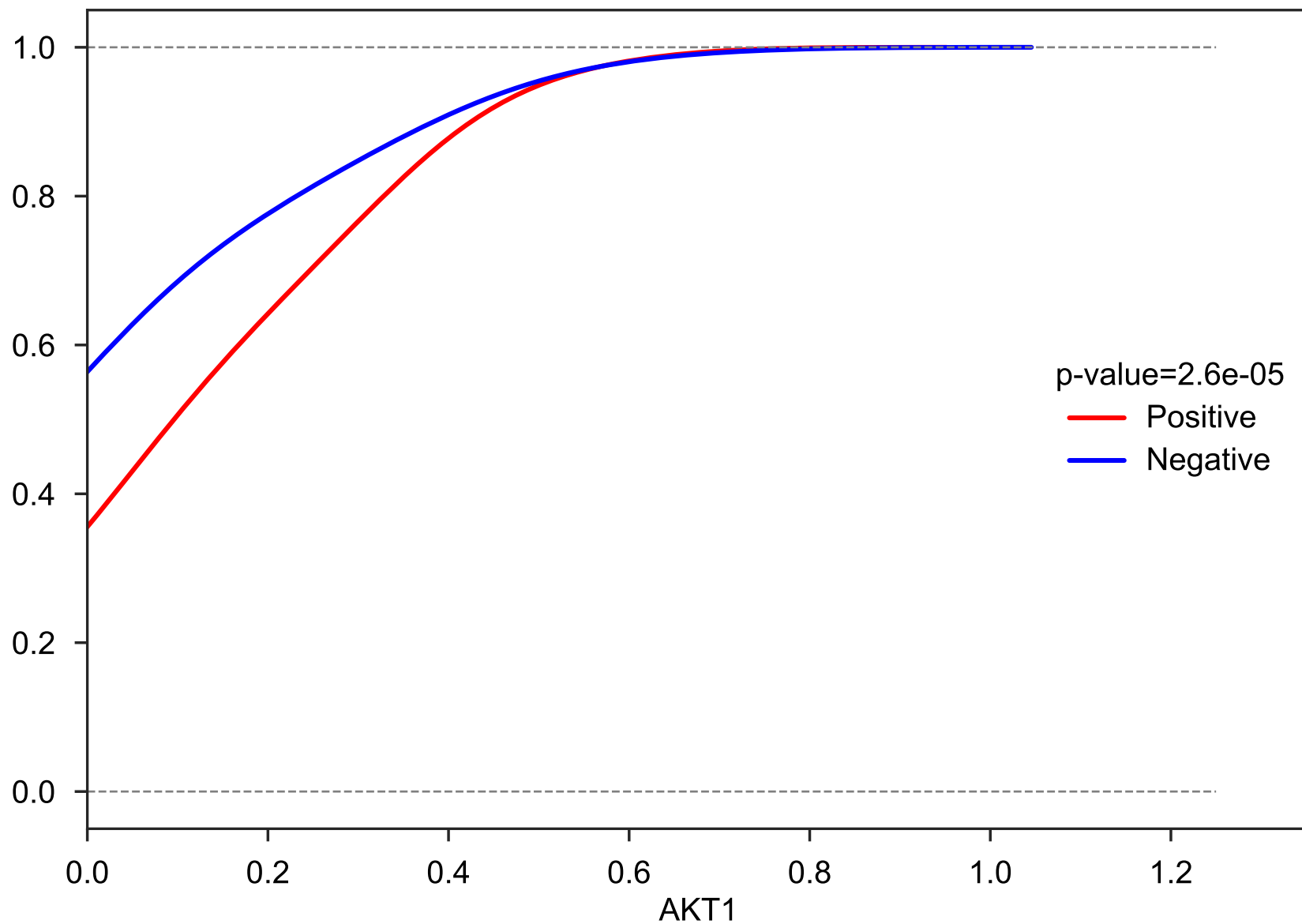

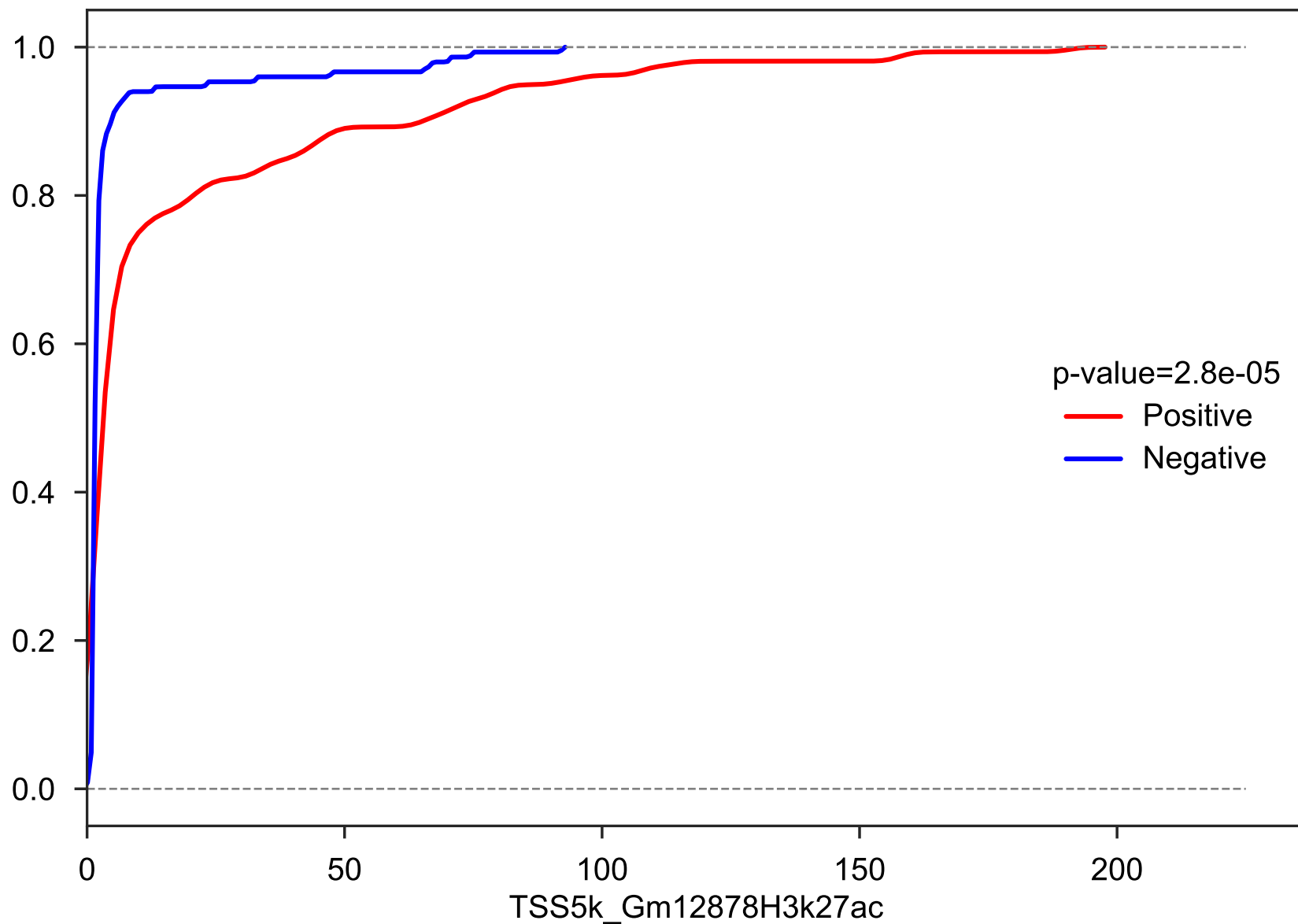

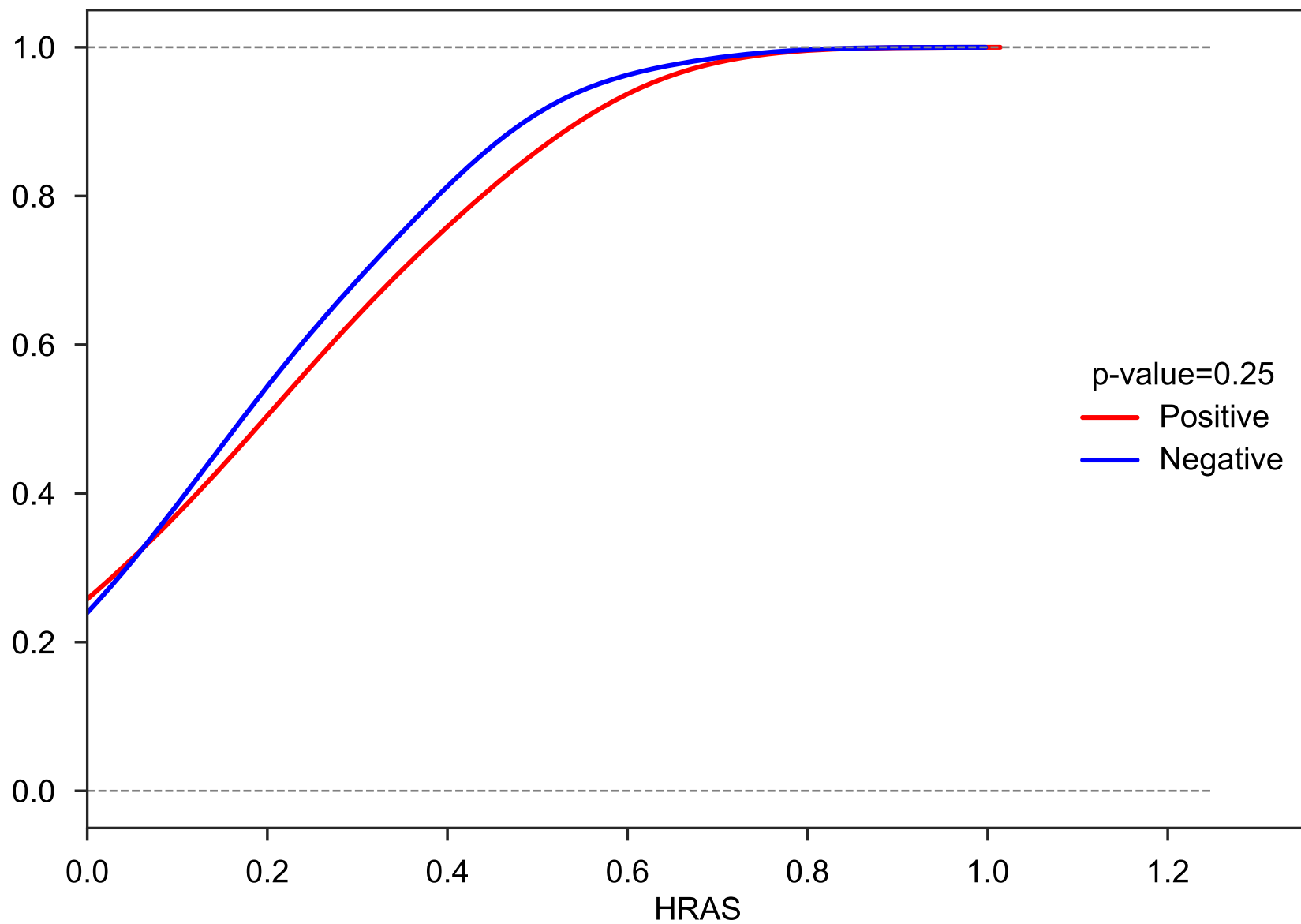

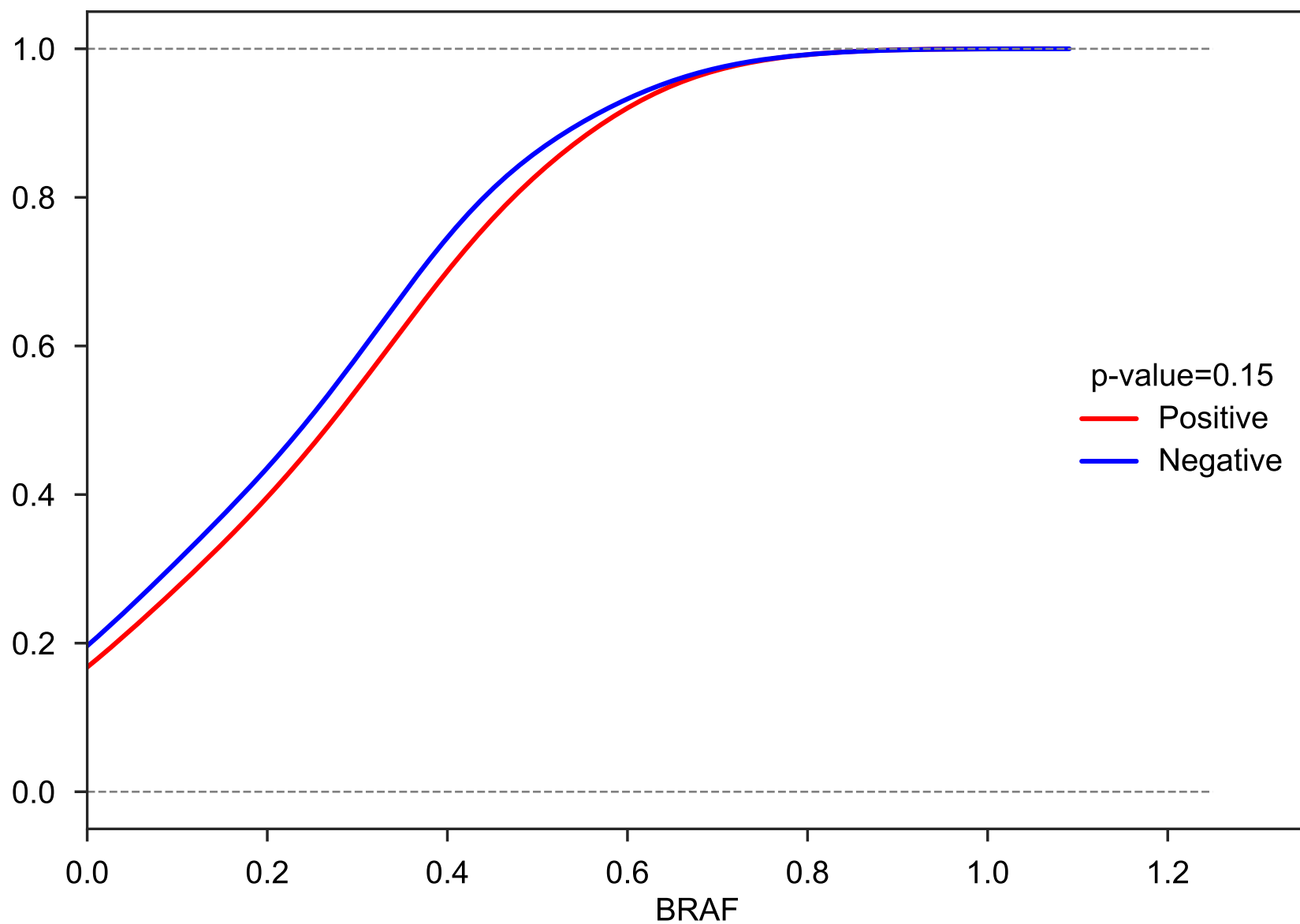

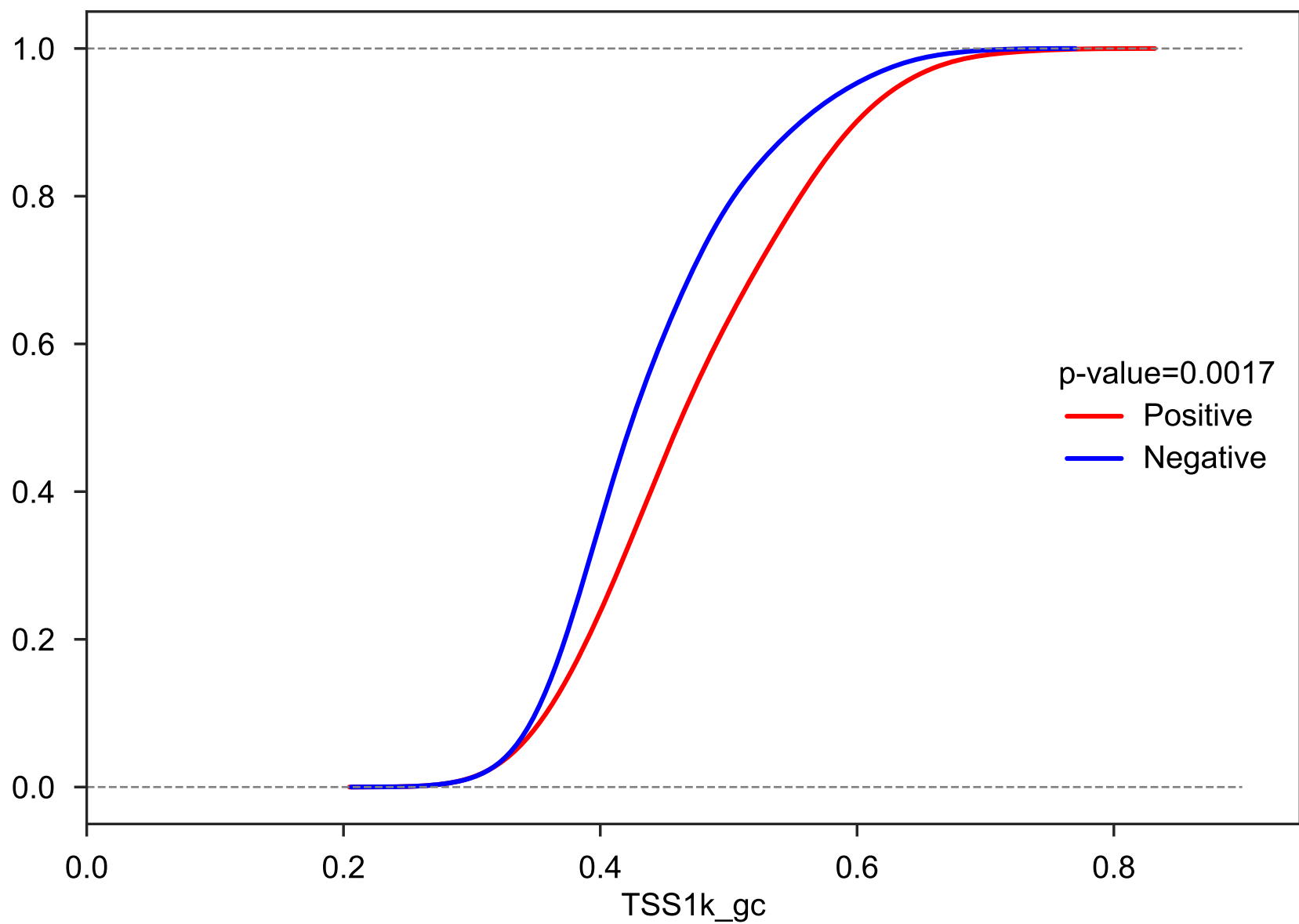

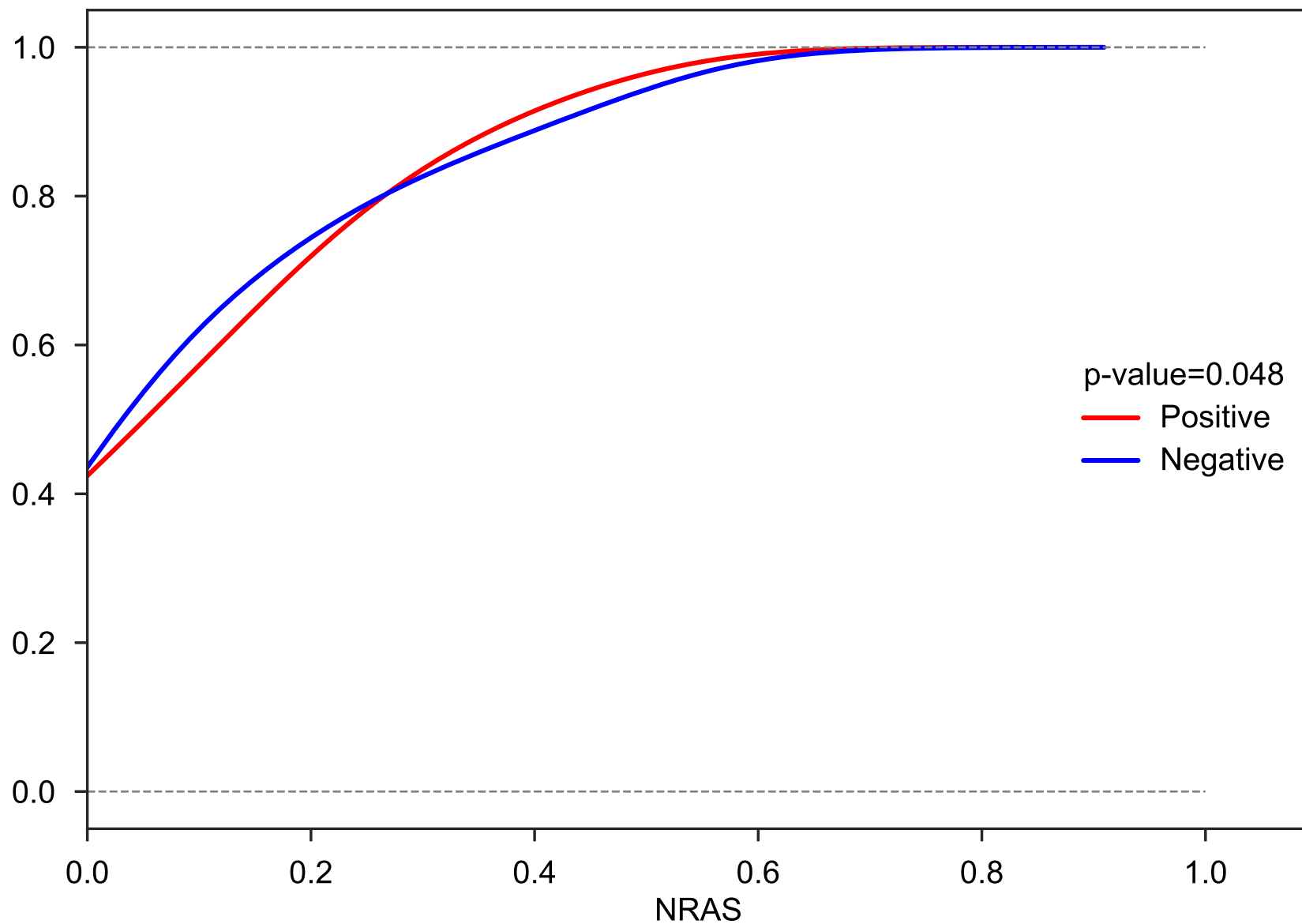

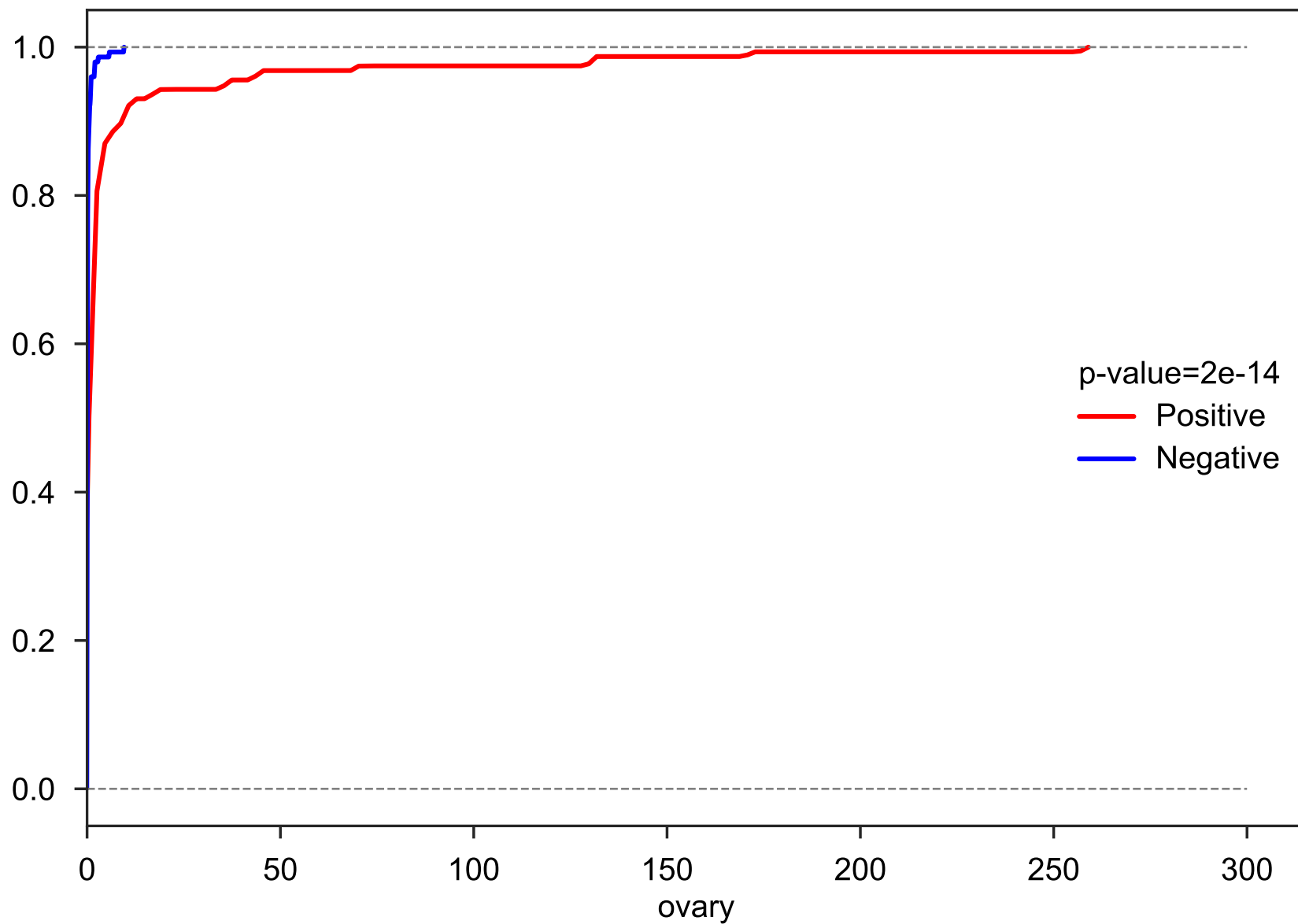

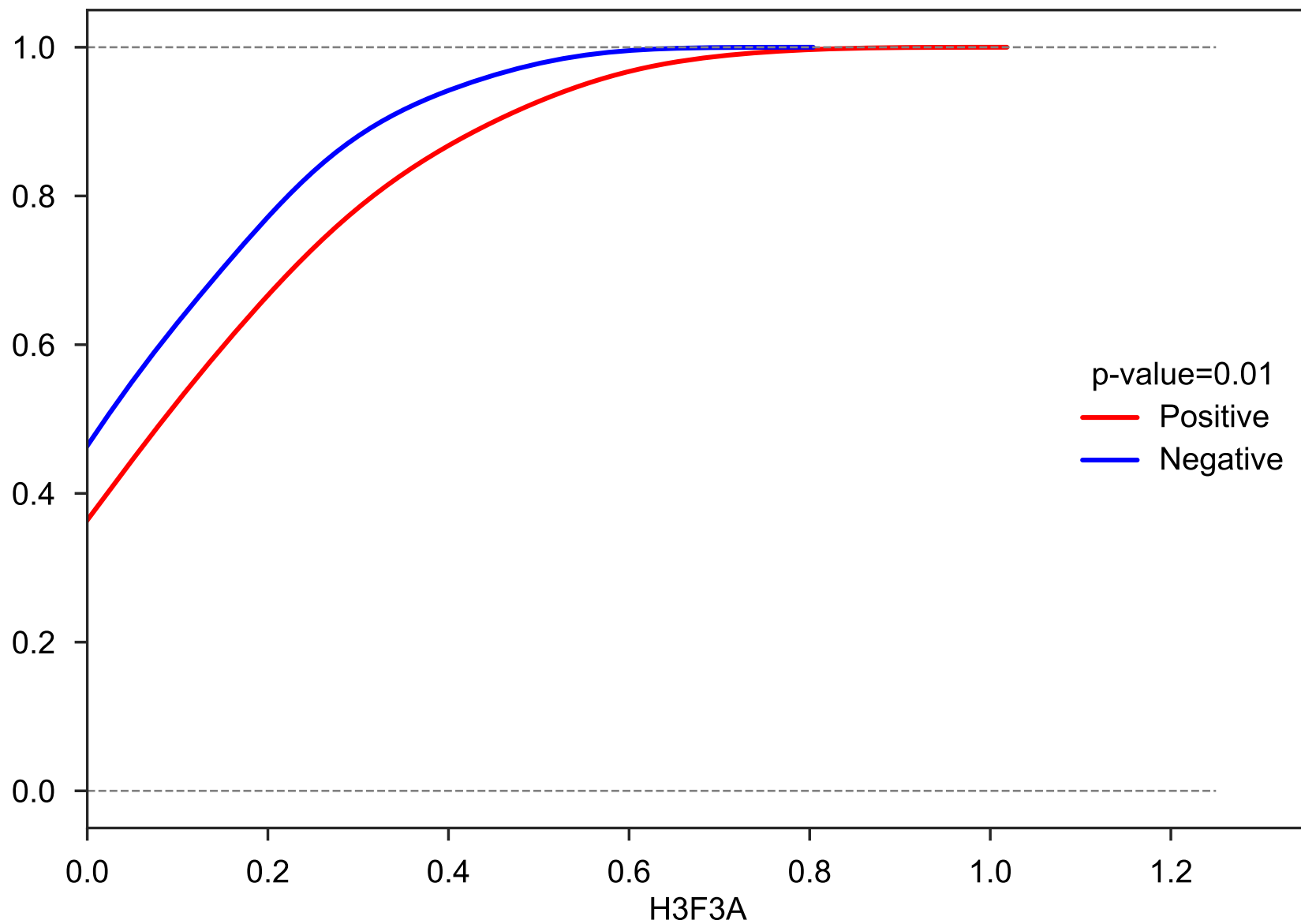

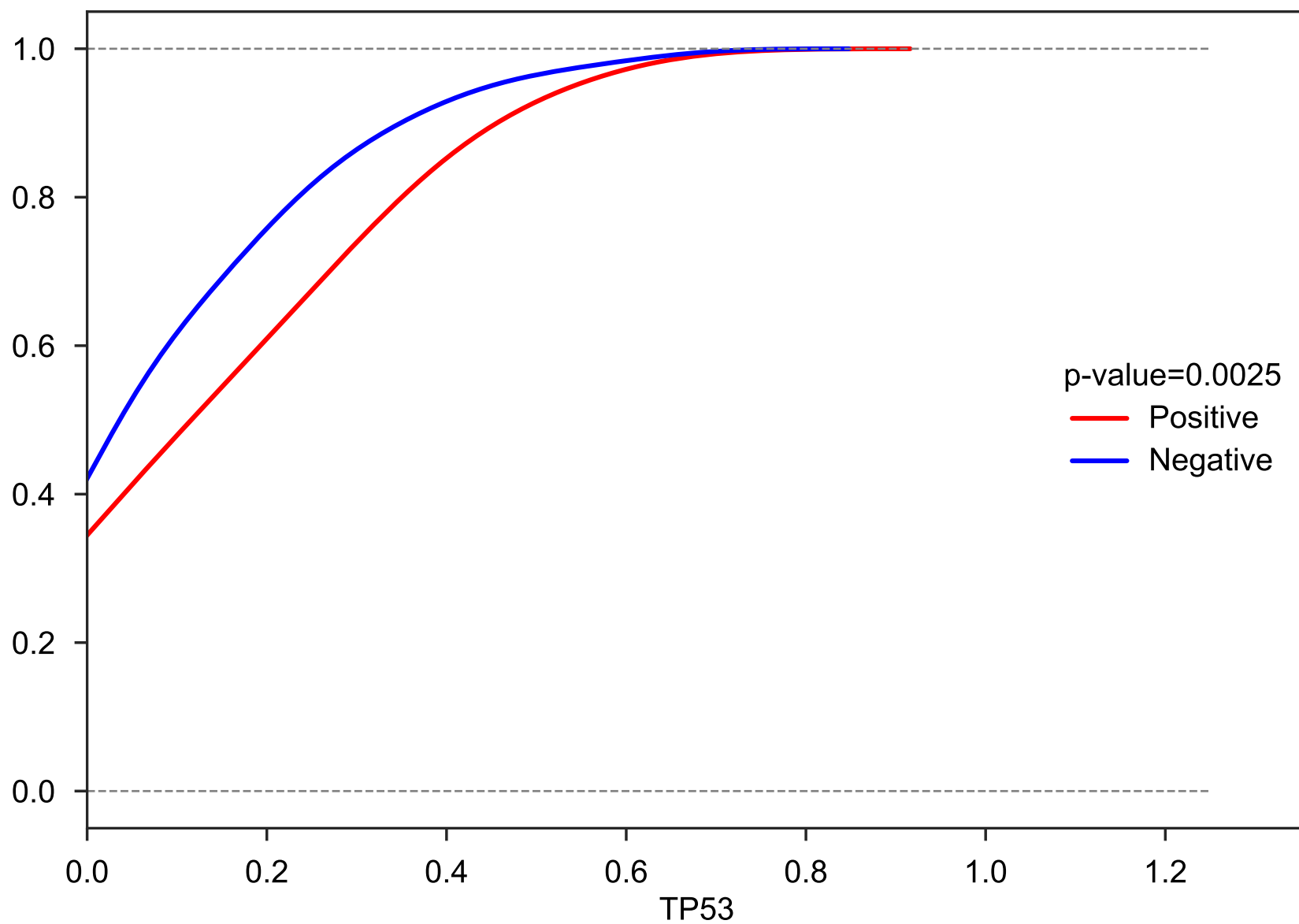

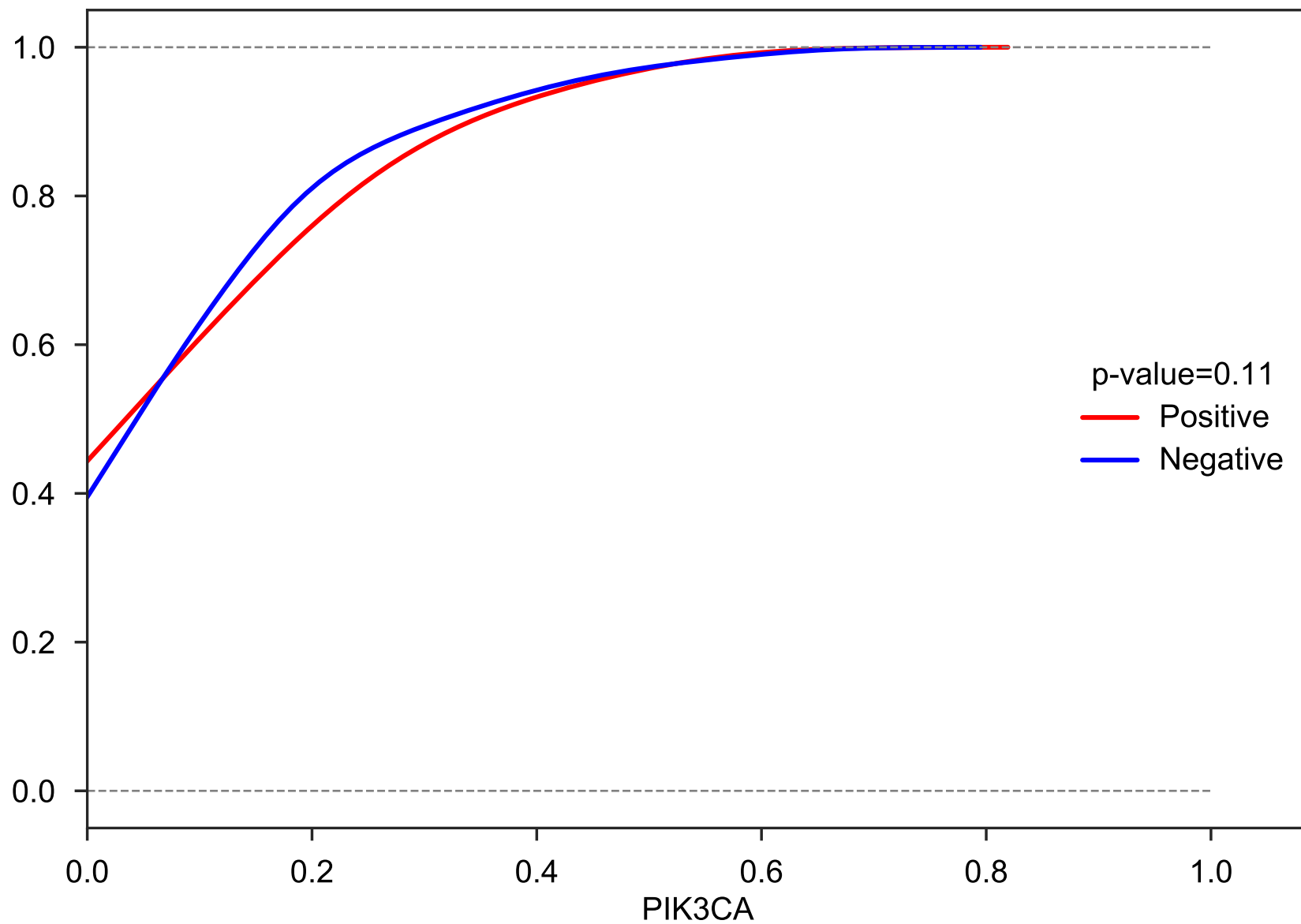

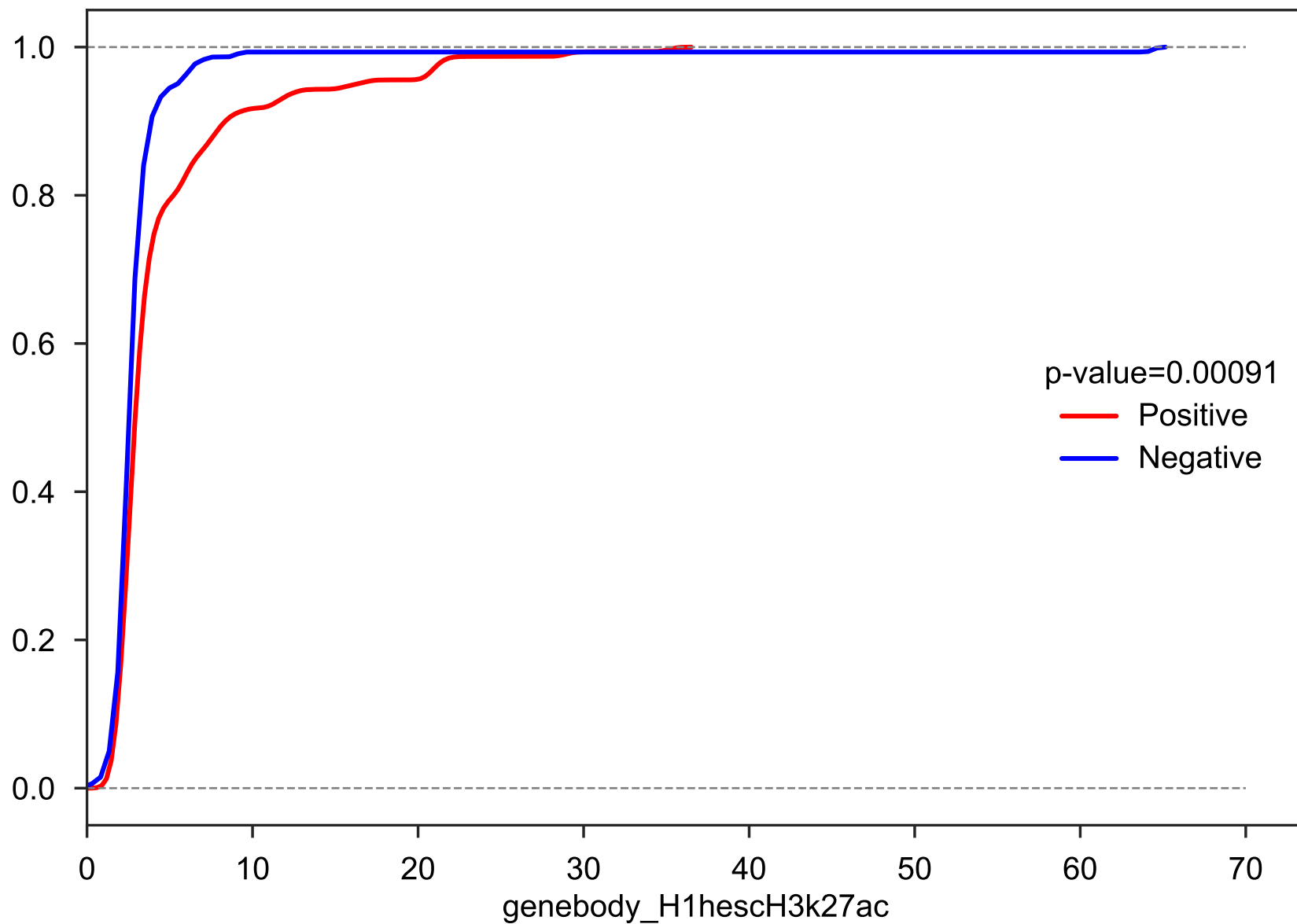

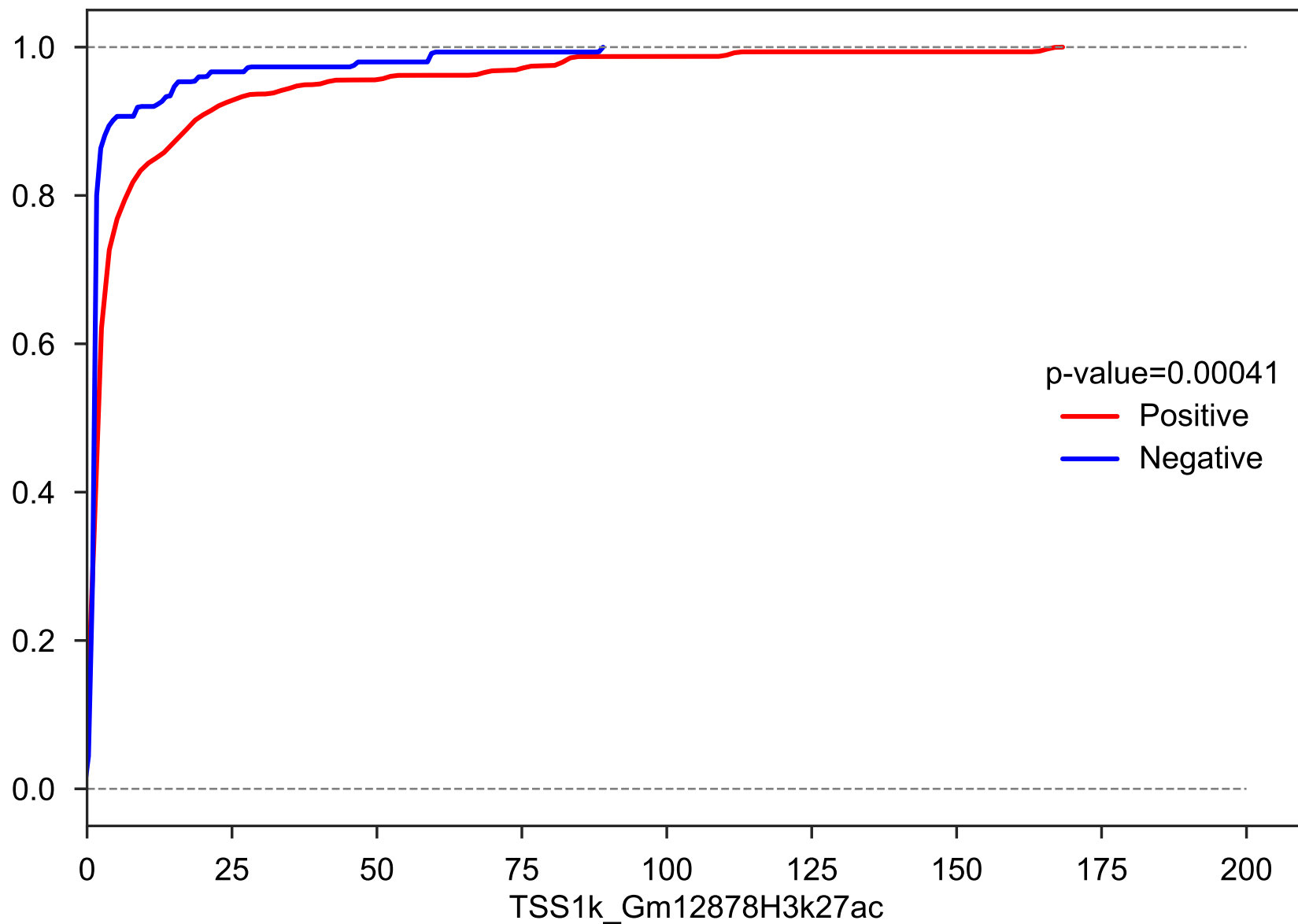

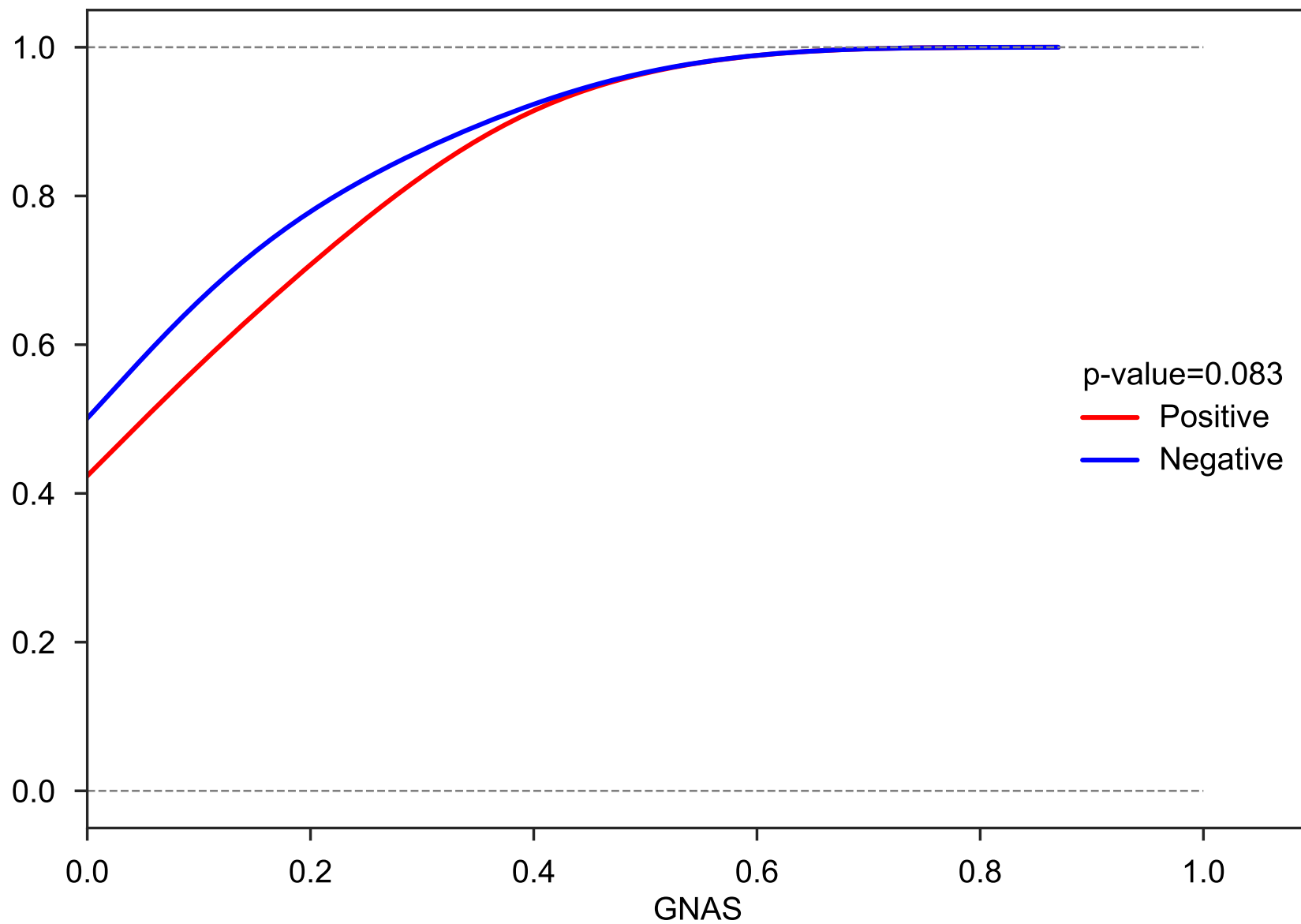

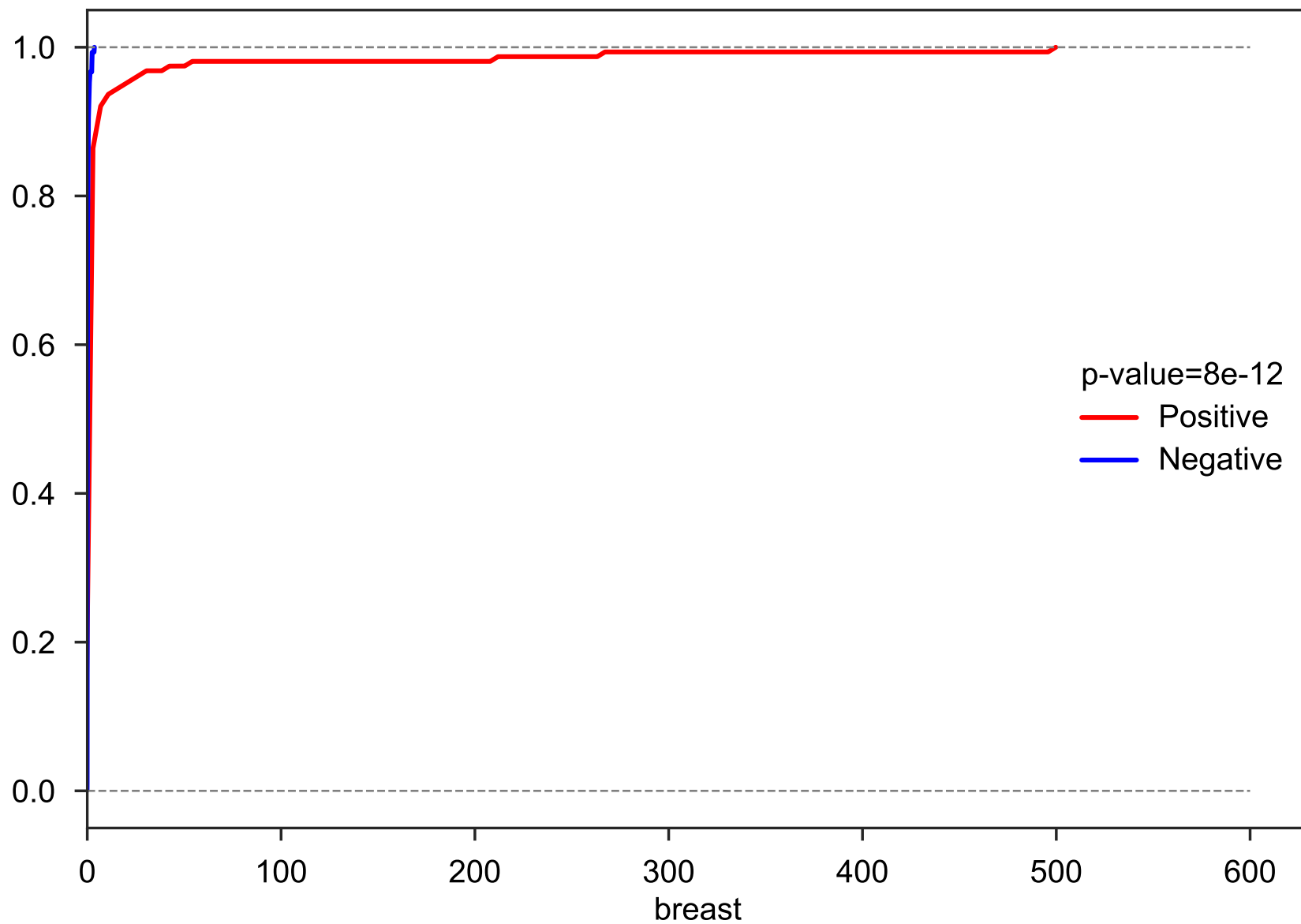

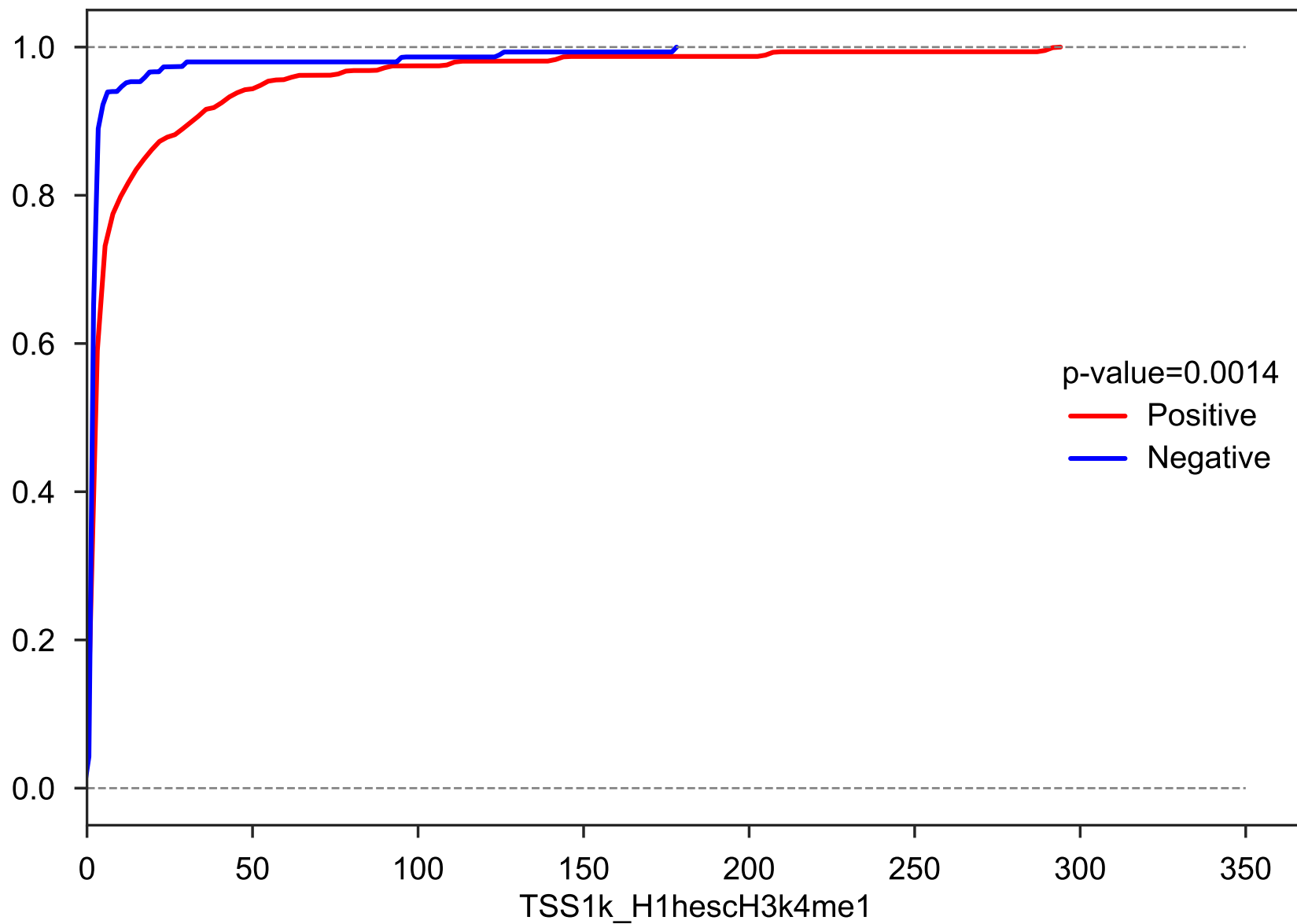

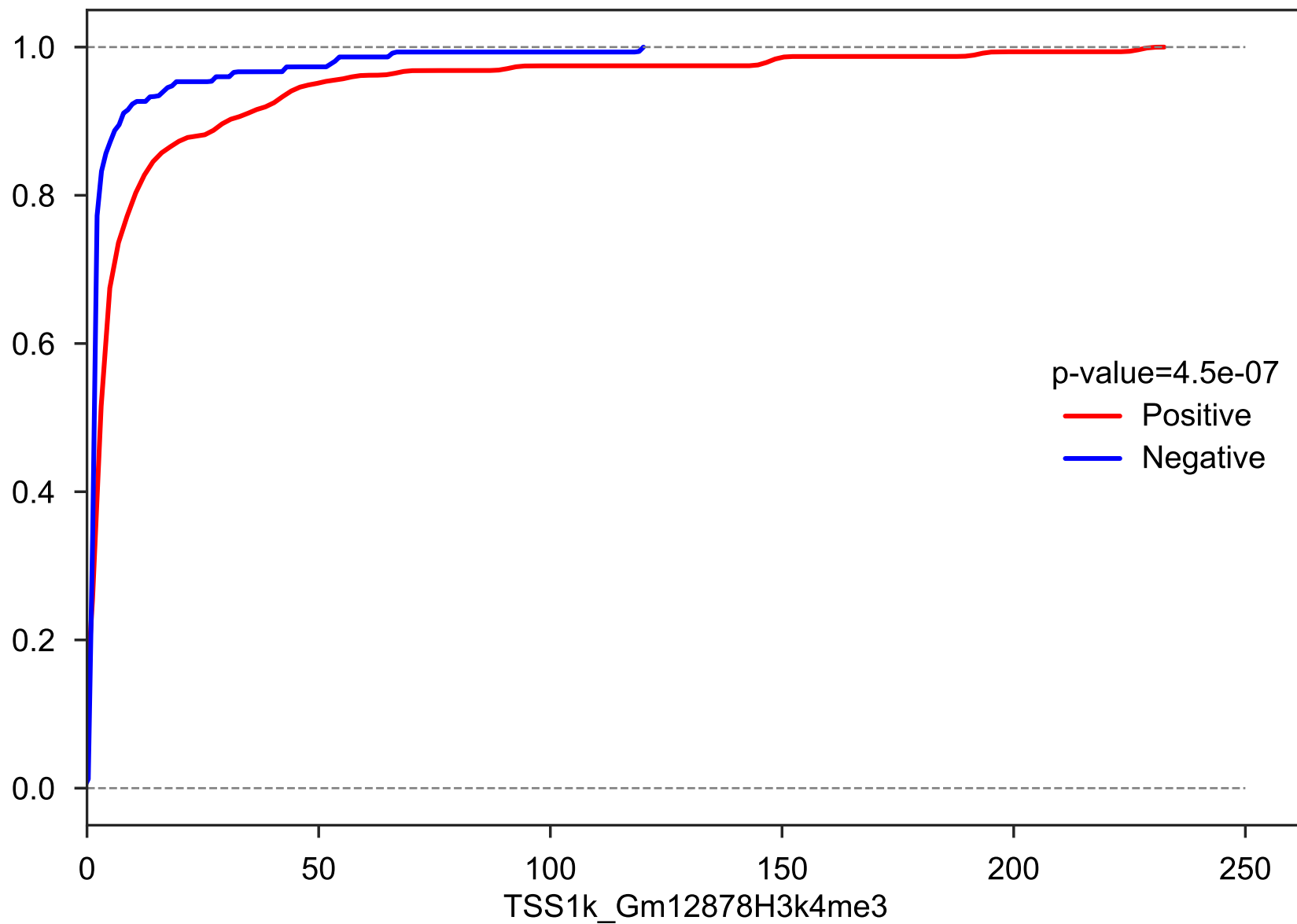

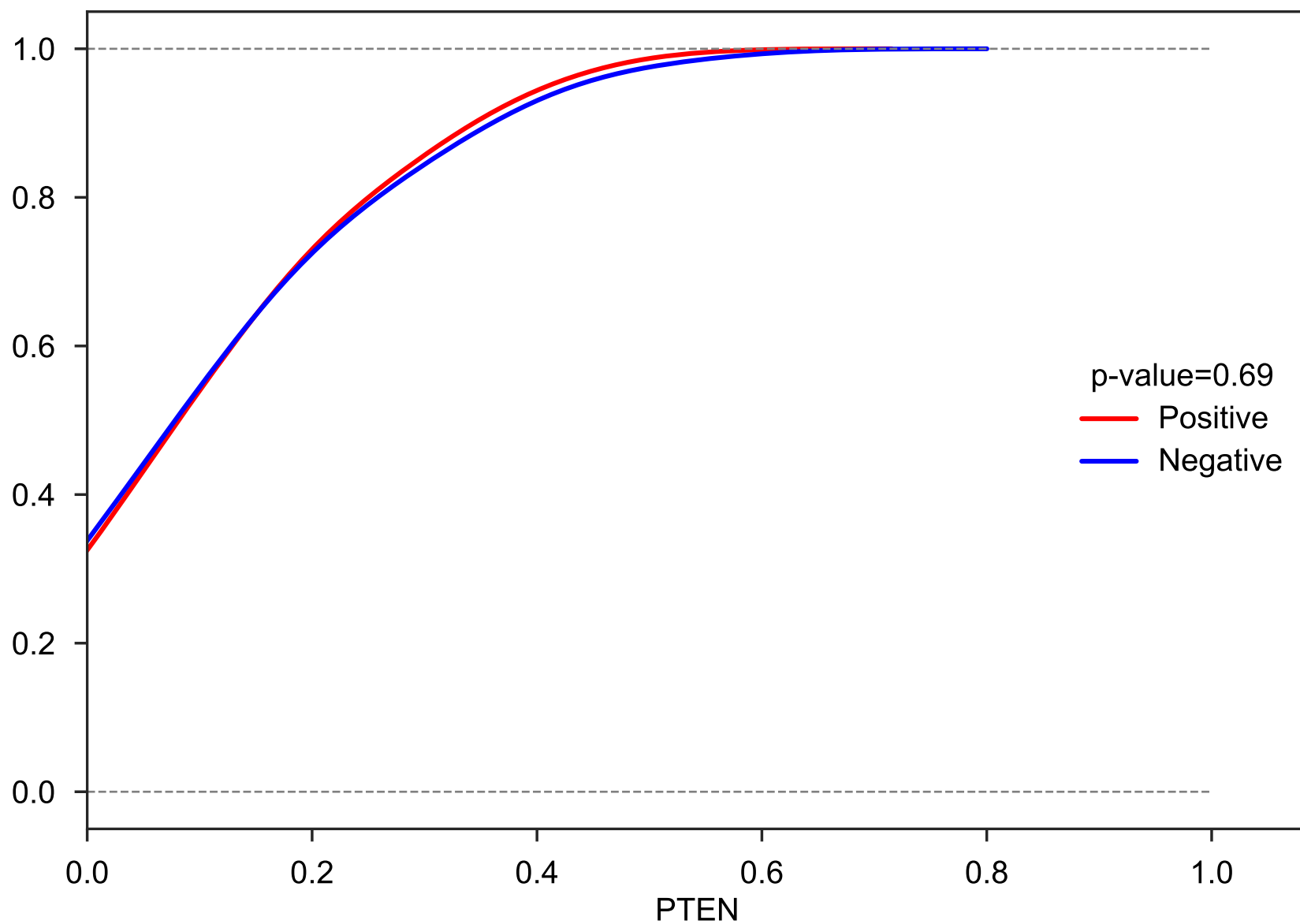

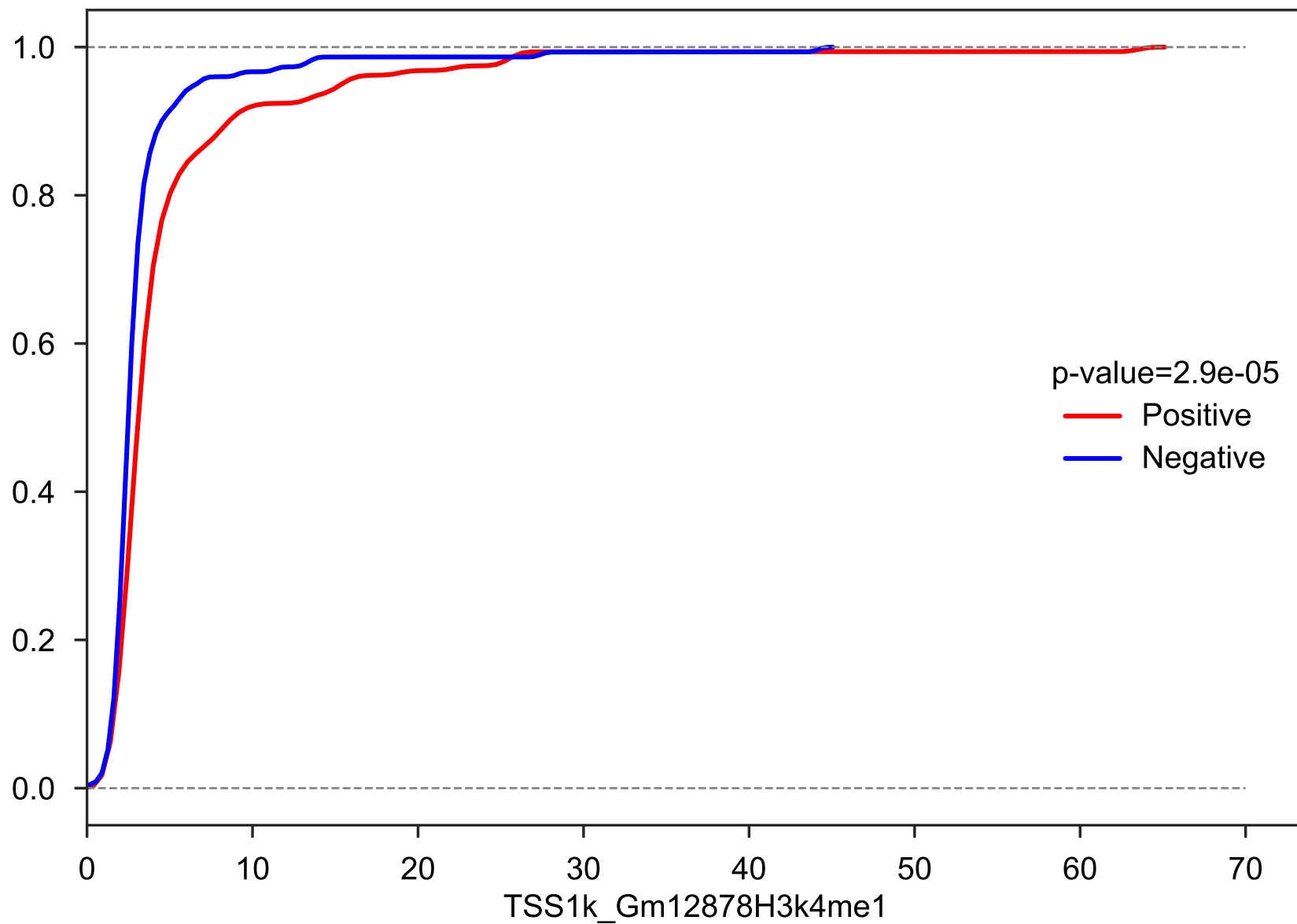

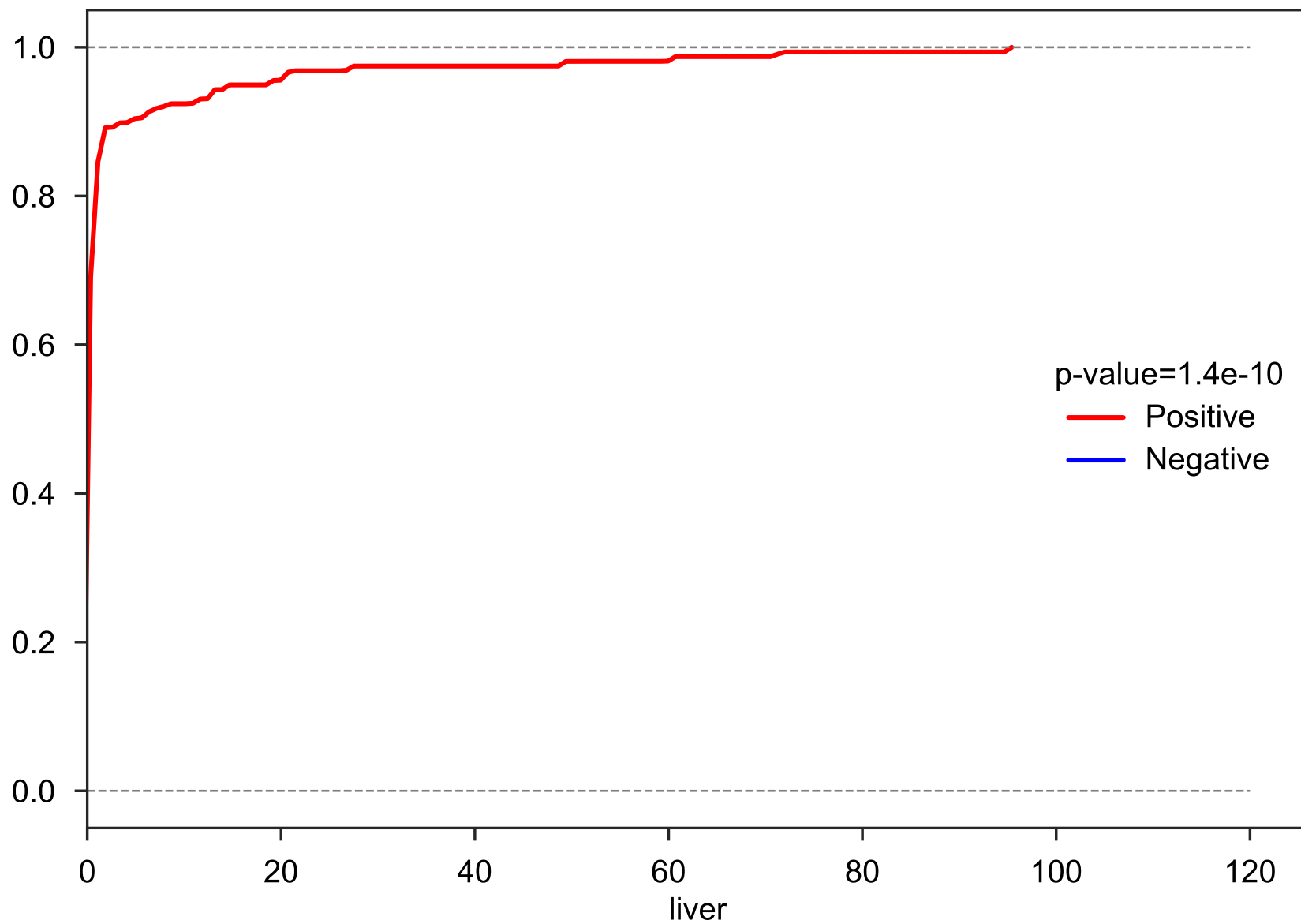

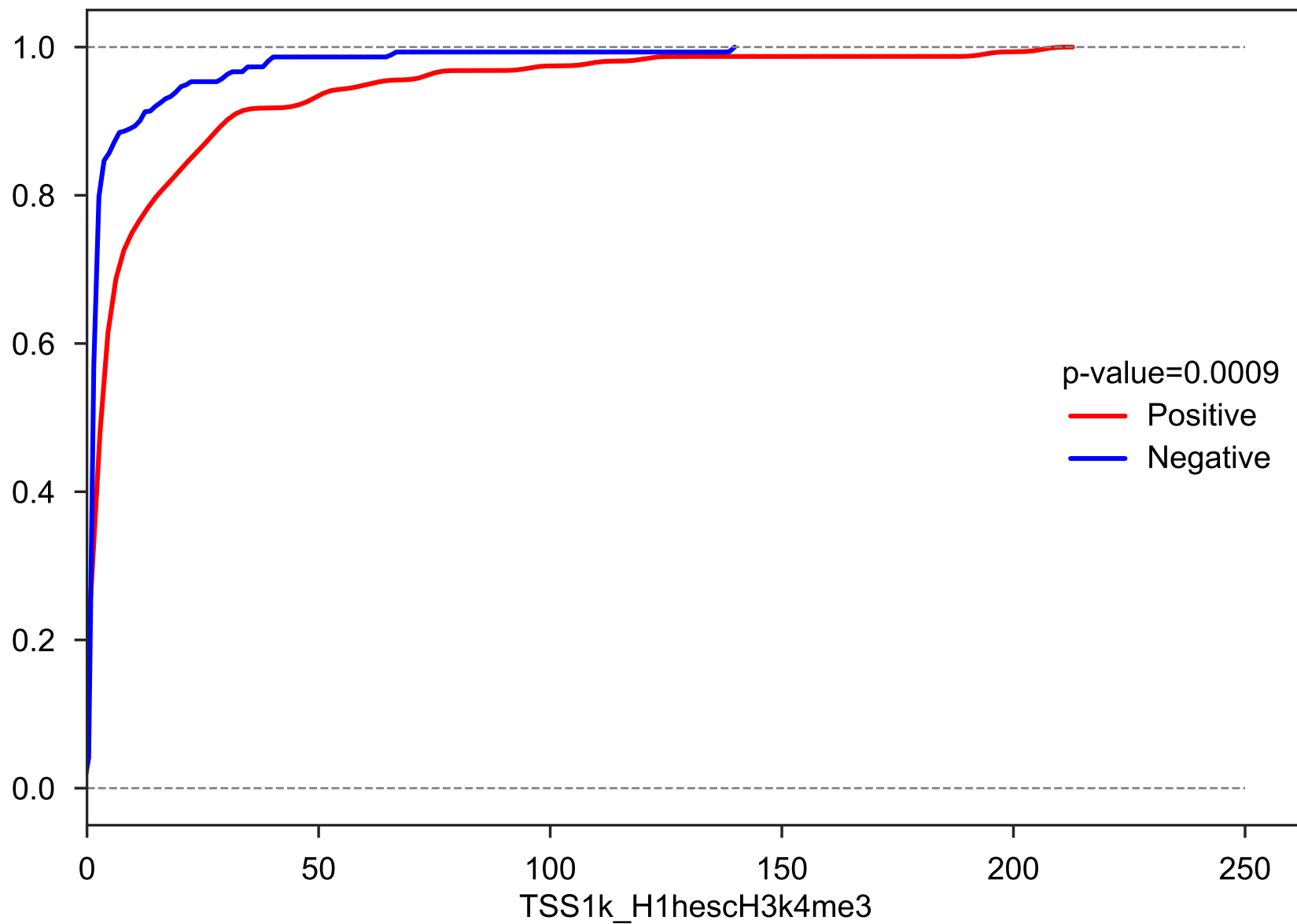

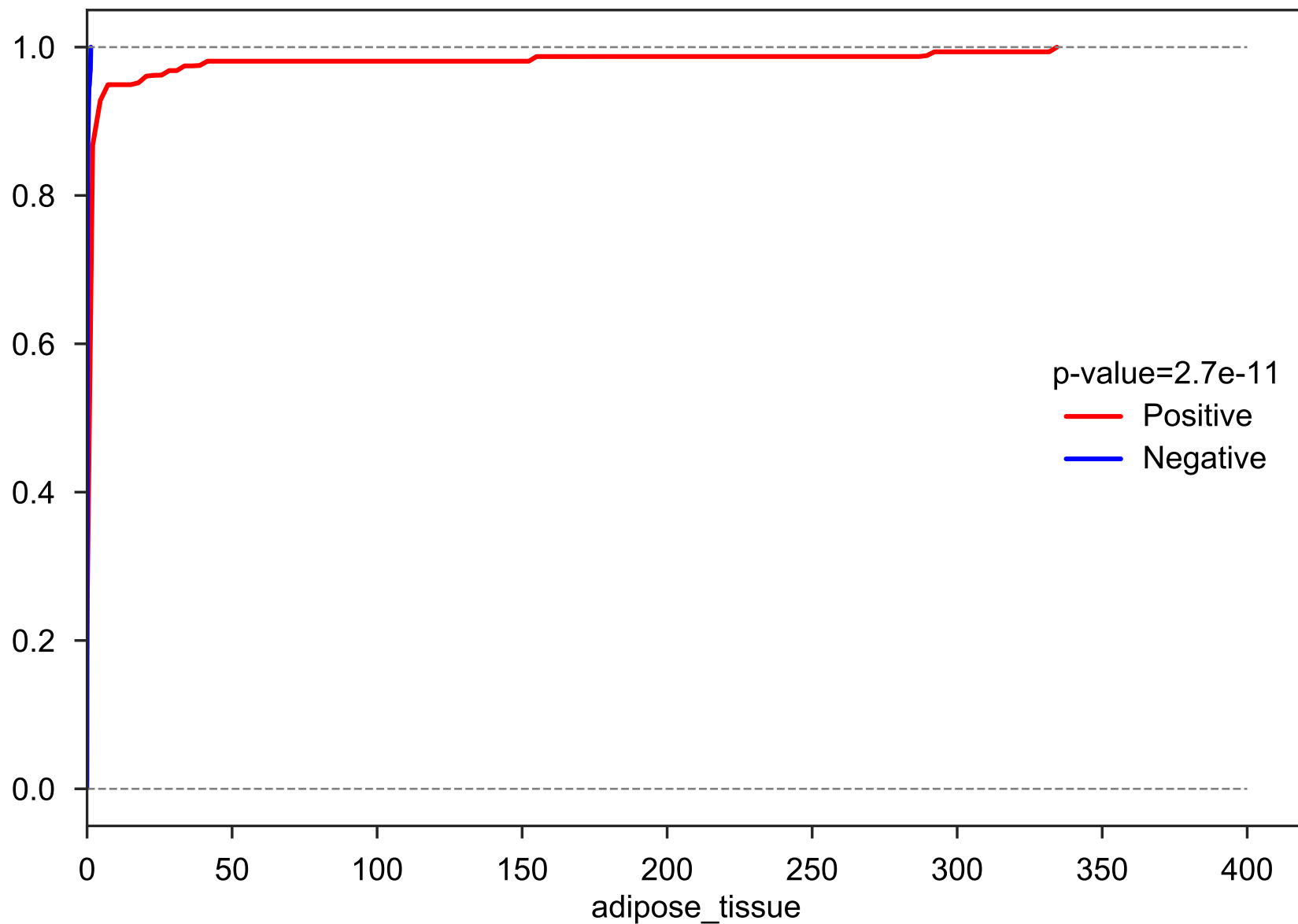

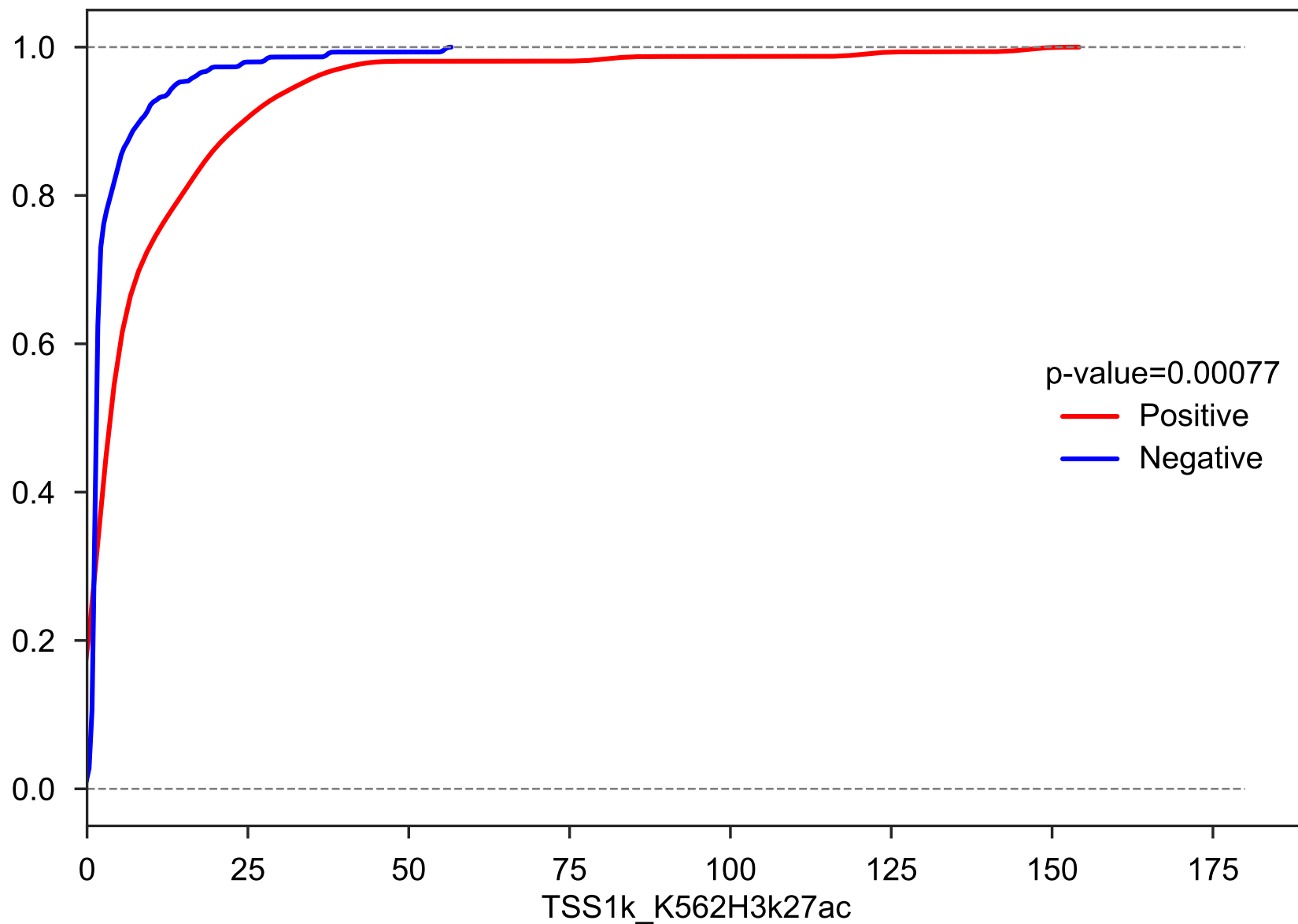

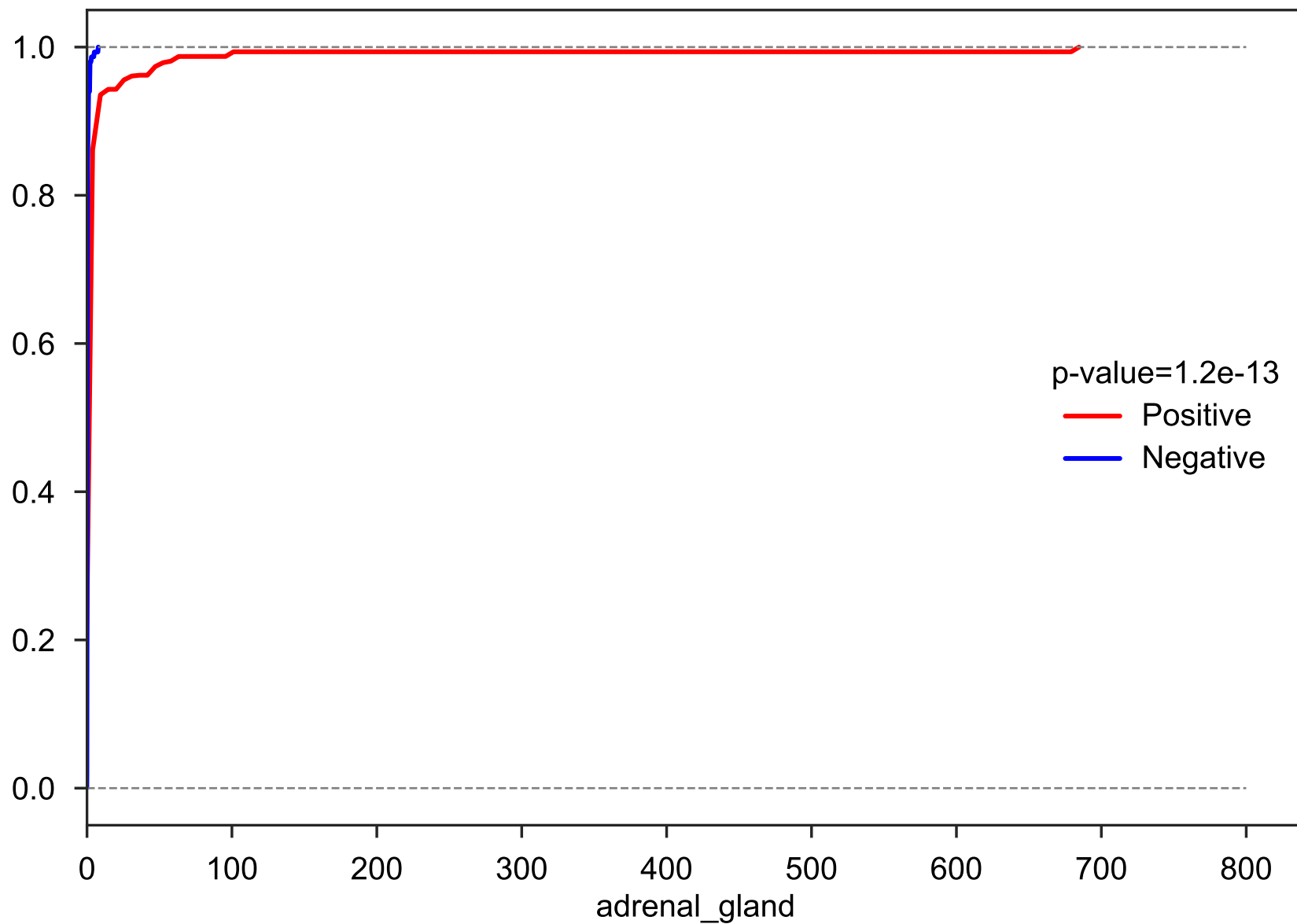

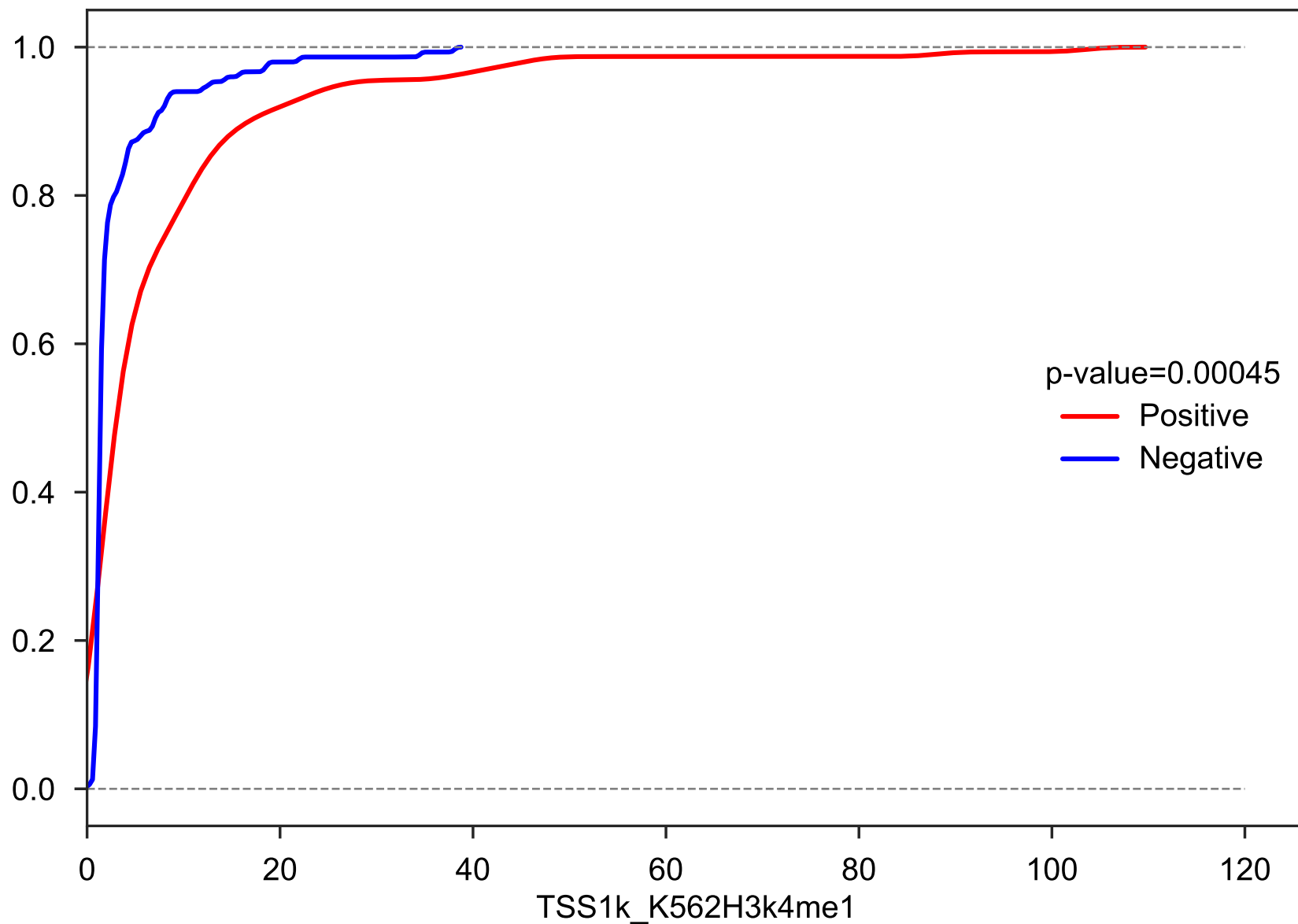

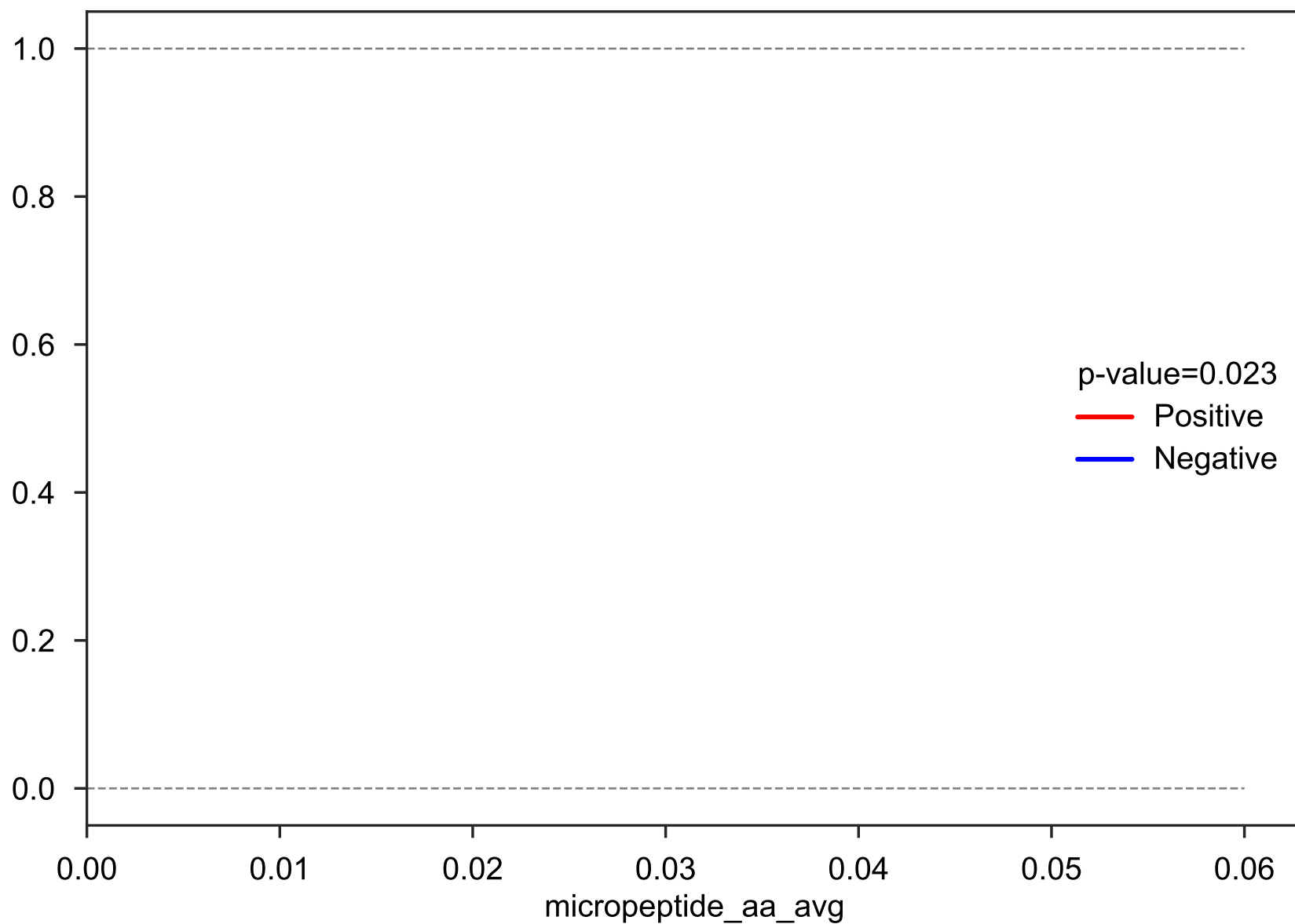

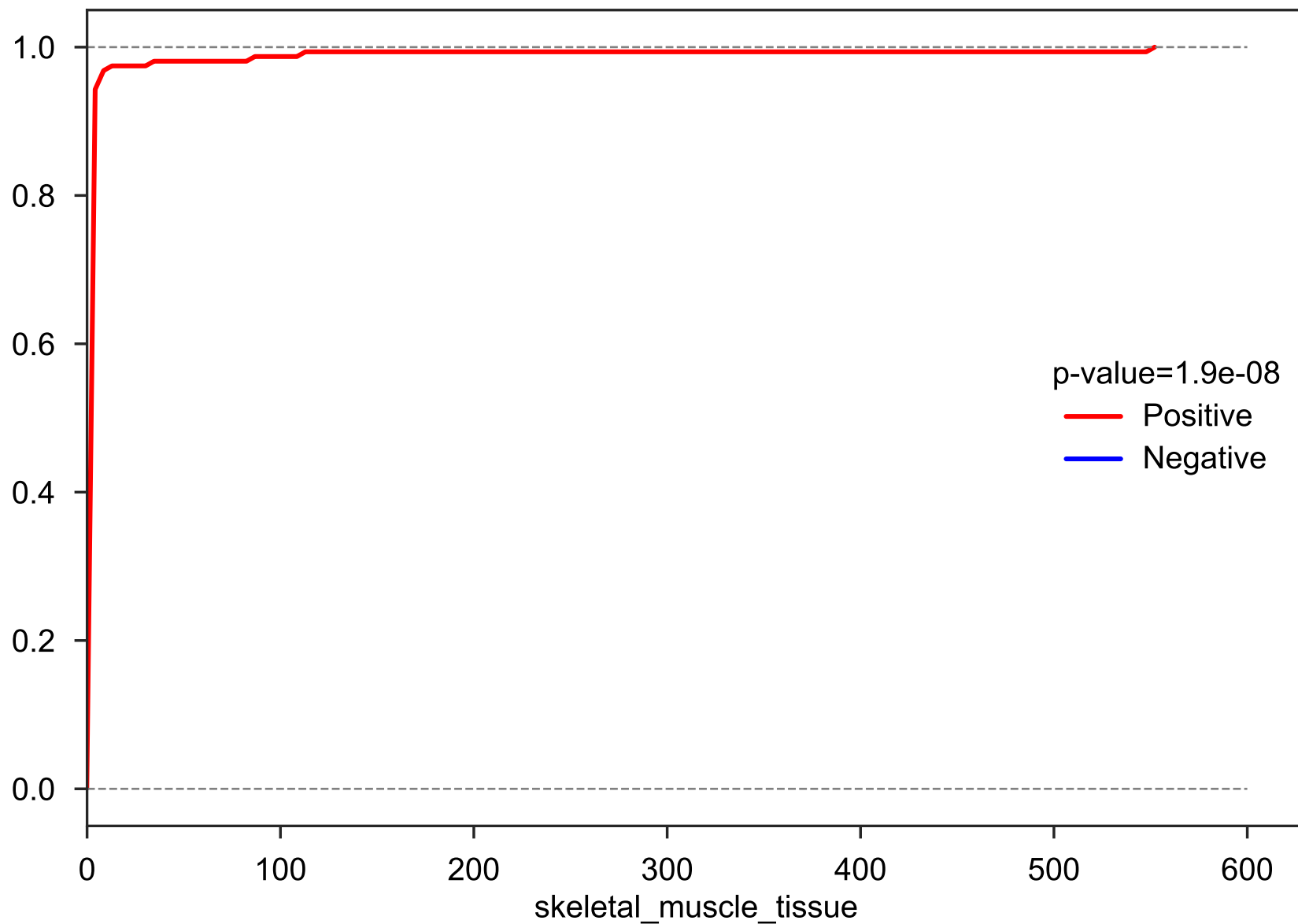

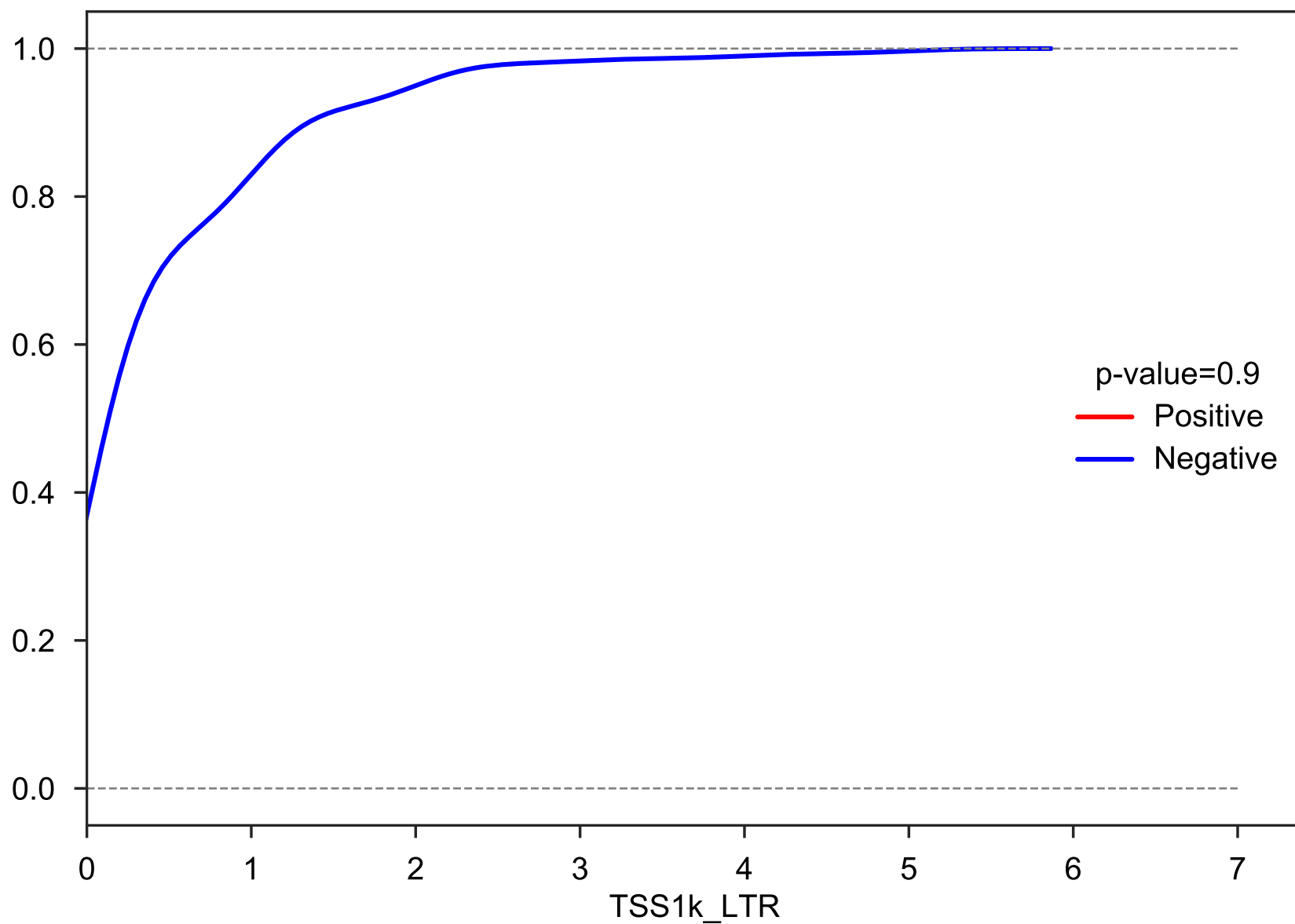

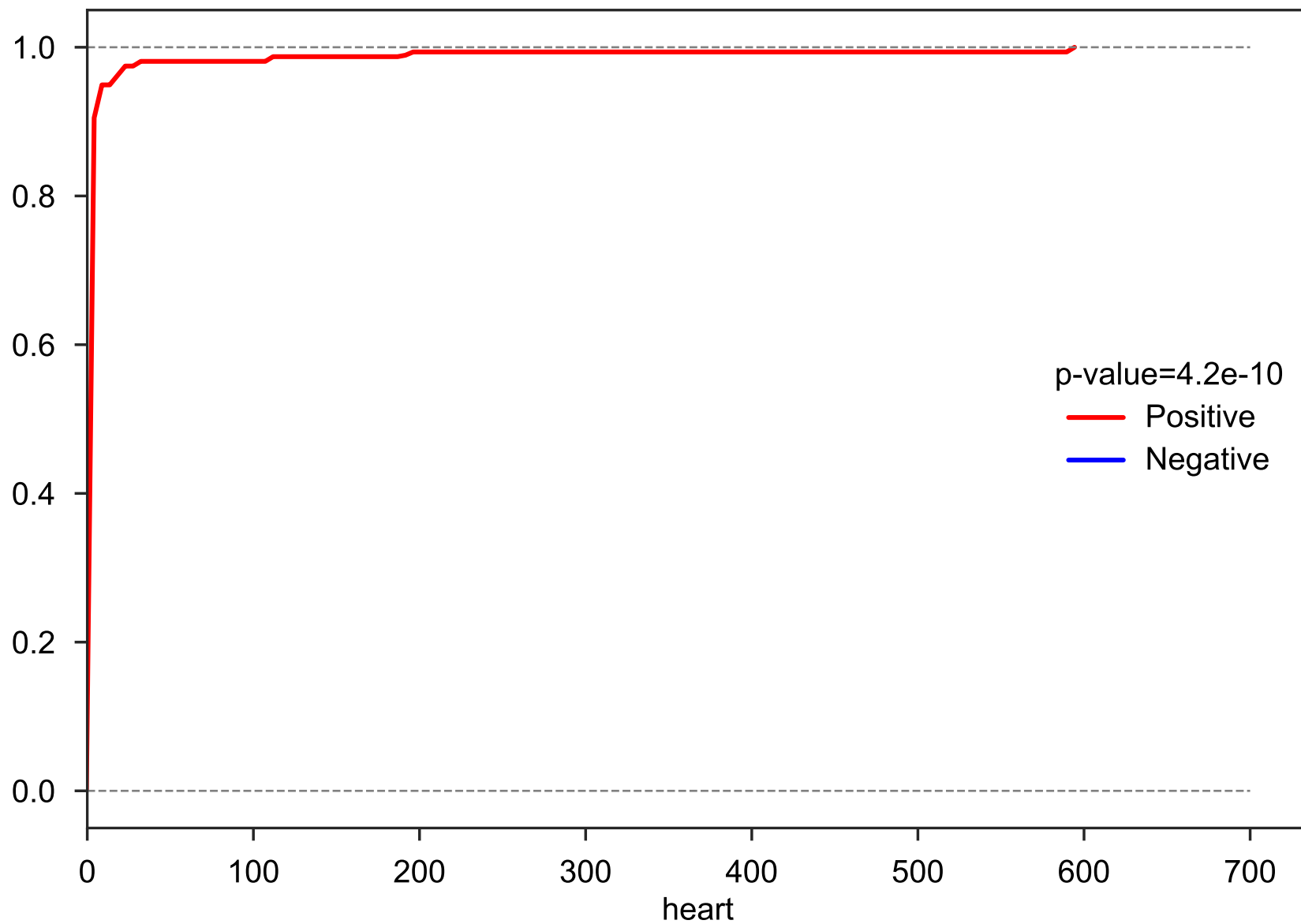

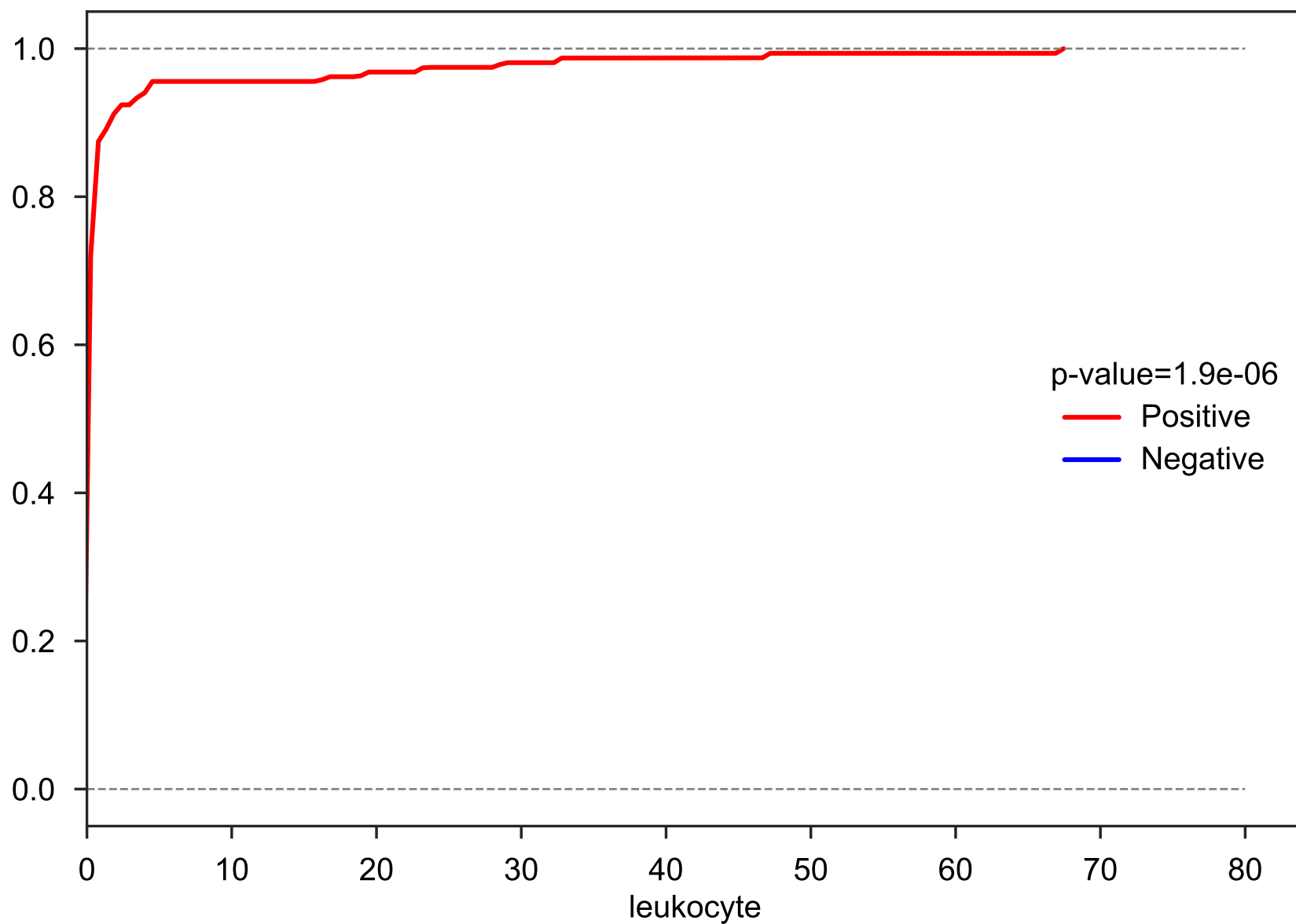

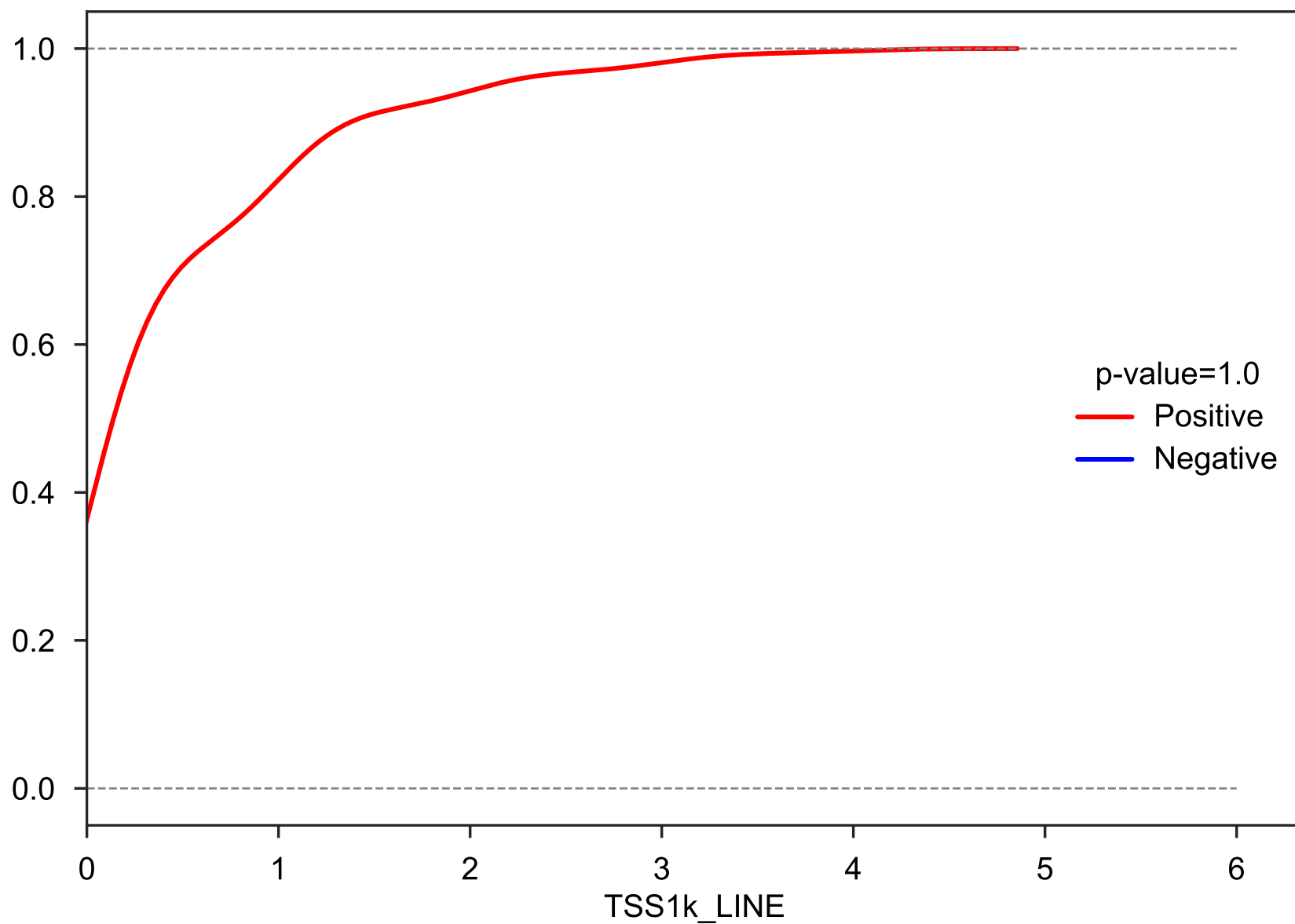

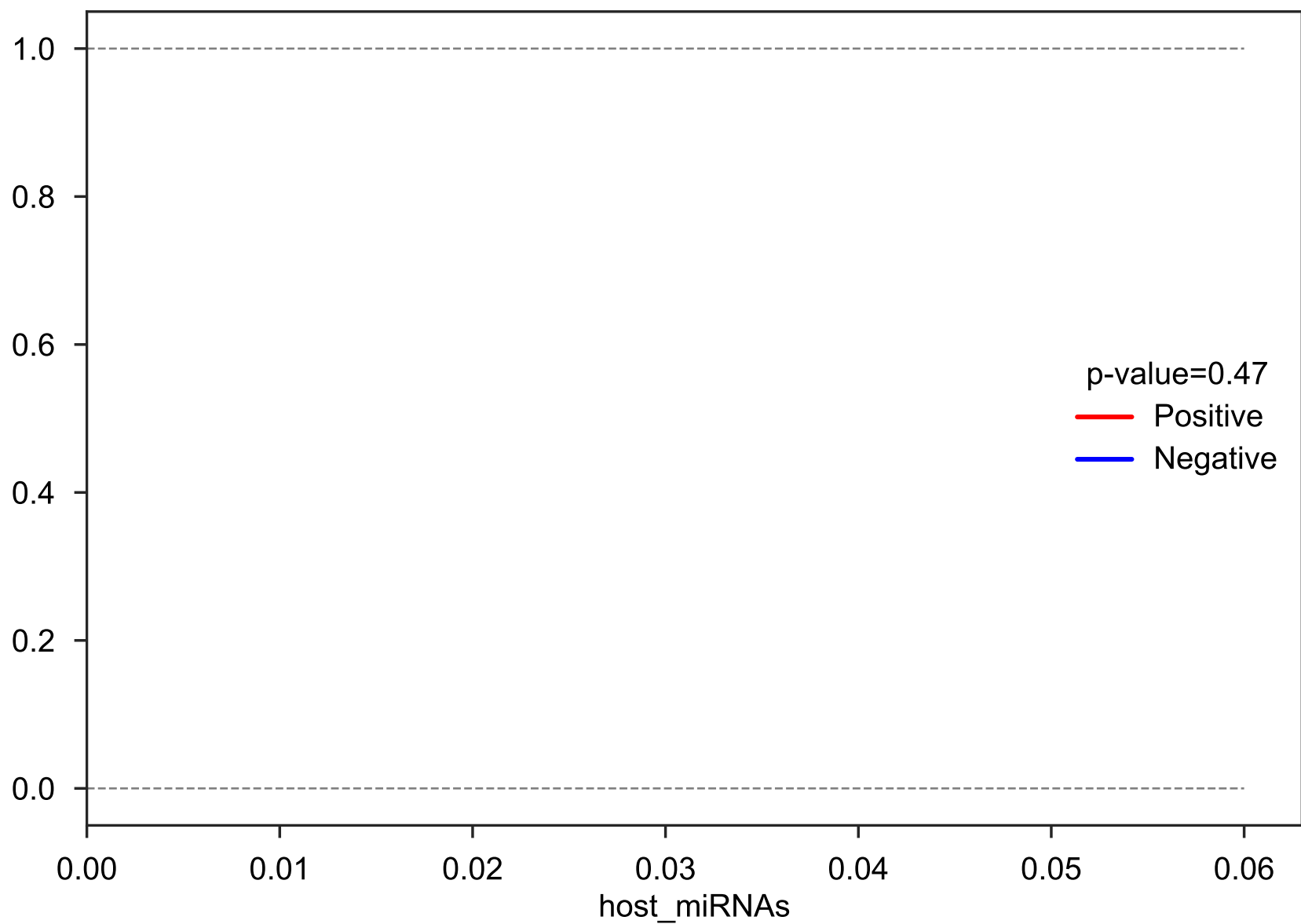

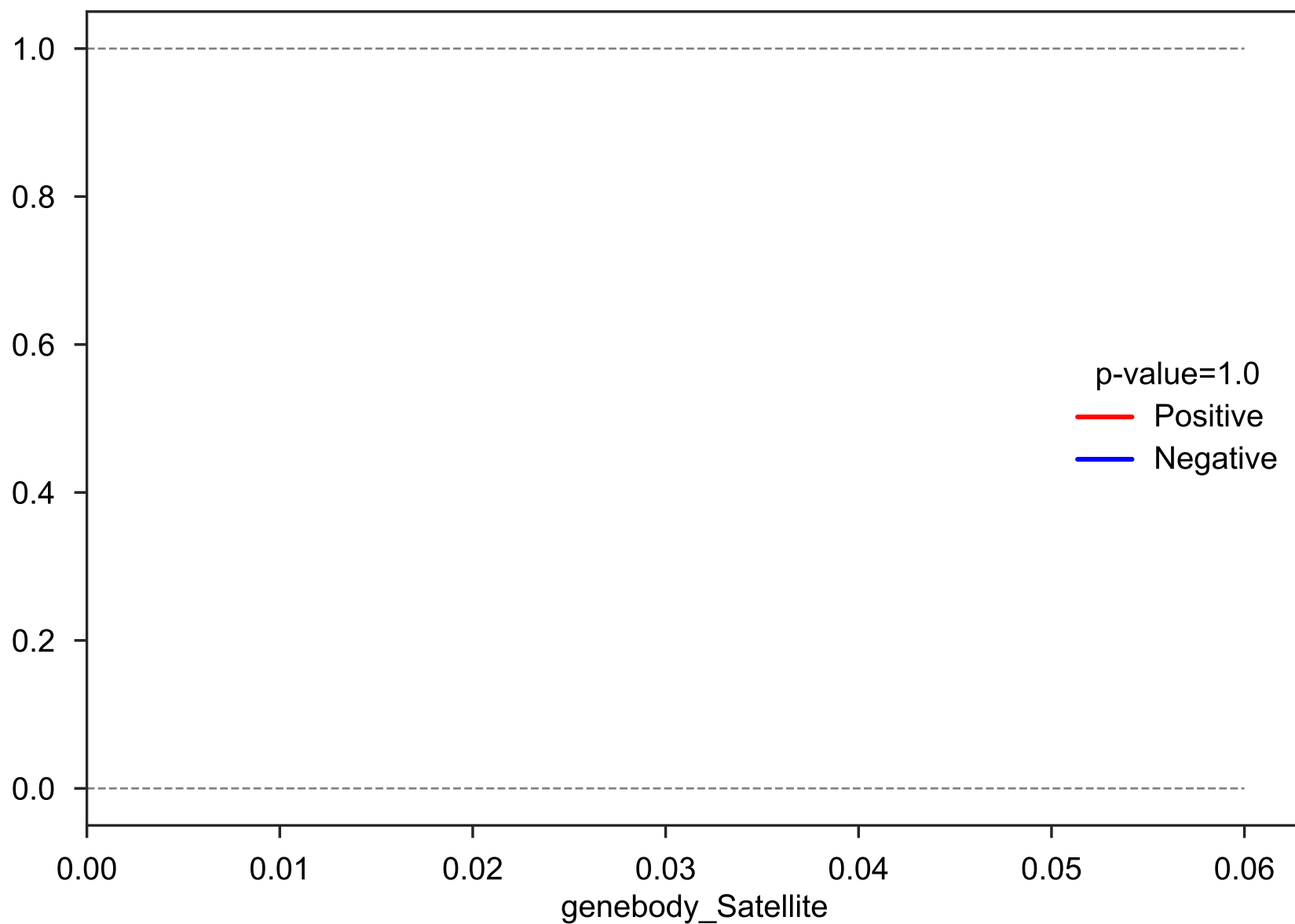

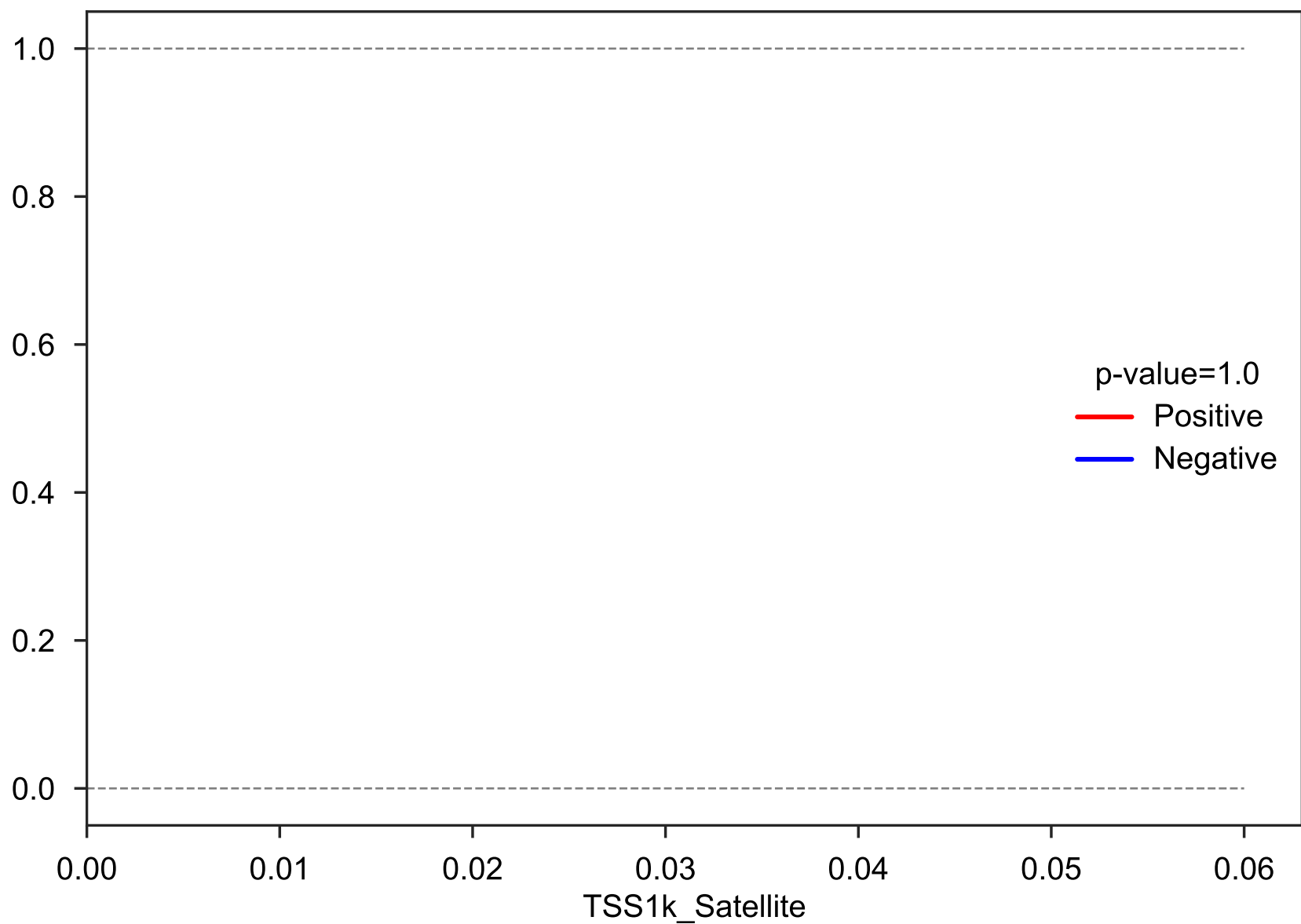

Supplement: Supplementary file 7 — Cumulative percentage curve of features. (PDF 208 kb) [file 12920_2018_436_MOESM7_ESM.pdf]

Gene length comparison

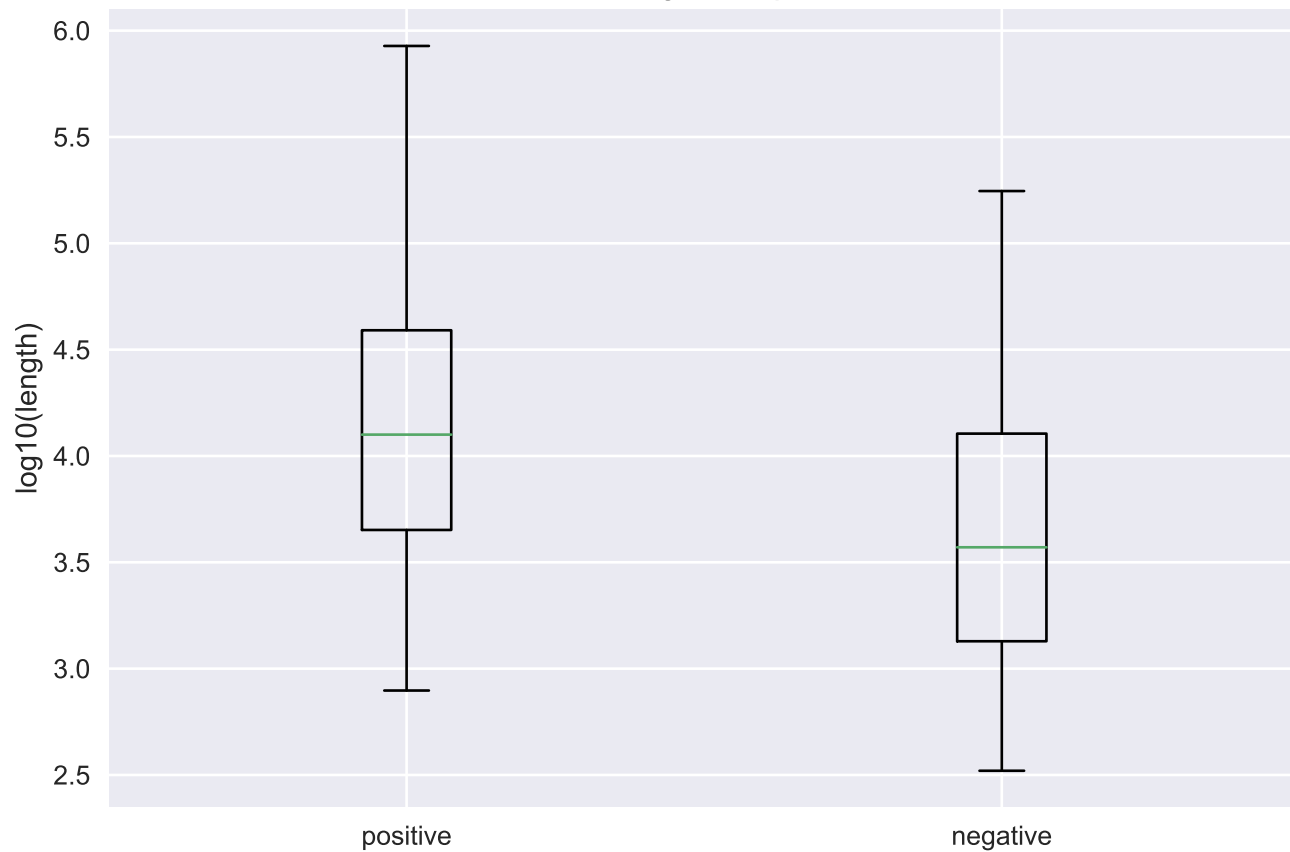

Transcript length comparison

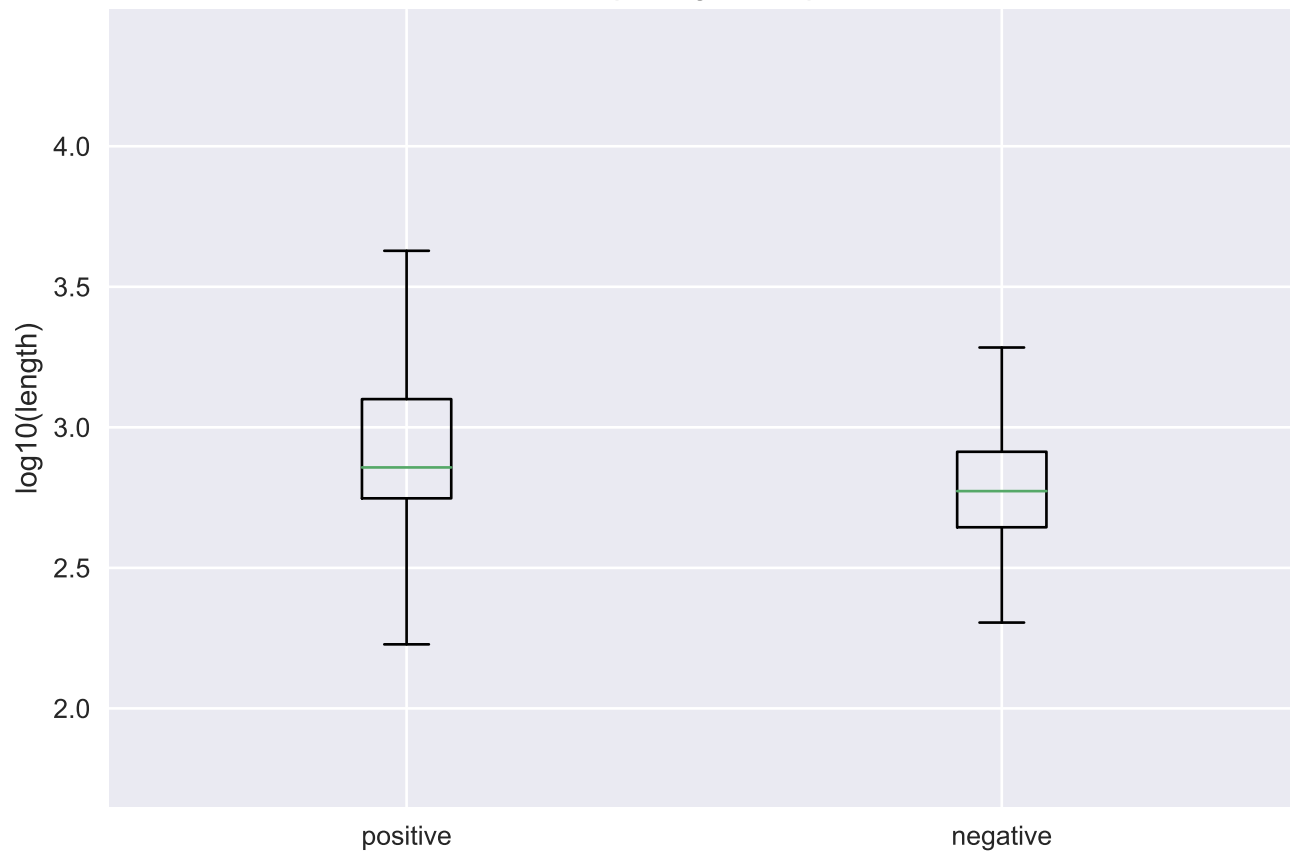

Supplement: Supplementary file 8 — Gene and transcript length distribution. (PDF 14 kb) [file 12920_2018_436_MOESM8_ESM.pdf]
