# Supplementary material for: CRlncRC: a machine learning-based method for cancer-related long noncoding RNA identification using integrated features
Source: BMC Med Genomics. 2018 Dec 31;11(Suppl 6):120. doi: 10.1186/s12920-018-0436-9 (PMC6311943; doi:10.1186/s12920-018-0436-9)
Supplement: Supplementary file 11 — Model parameters. (PDF 46 kb) [file 12920_2018_436_MOESM11_ESM.pdf]

Machine learning algorithms parameter setting for CRlncRC

RF (Random Forest)

- **n_estimators**: The number of trees in the forest was set to 100.
- **criterion**: The function to measure the quality of a split was set to 'gini'.
- **others**: Default.

```
clf = RandomForestClassifier(n_estimators=100, criterion='gini')
```

NB (Naïve Bayes)

- **alpha**: Additive (Laplace/Lidstone) smoothing parameter was set to 5.
- **others**: Default.

```
clf = BernoulliNB(alpha = 5)
```

SVM (Support Vector Machines)

GridSearchCV

- **clf**: This is assumed to implement the scikit-learn estimator interface. We use SVC with 'rbf' kenel.
- **param_grid**: This enables searching over any sequence of parameter settings. C_range, gamma_range was used.
- **cv**: The cross-validation splitting strategy was set to 10.
- **others**: Default.

```
clf = SVC(kernel = 'rbf', probability = True)
C_range = np.logspace(-2, 10 ,13)
gamma_range = np.logspace(-9, 3, num = 13)
param_grid = dict(gamma = gamma_range, C = C_range)
grid_search = GridSearchCV(clf, param_grid, cv=10, n_jobs=-1)
```

SVC

- **kernel**: Specifies the kernel type to be used in the algorithm.
- **C**: Penalty parameter C of the error term was set to the best C by GridSearchCV.
- **gamma**: Kernel coefficient was set to the best gamma by GridSearchCV.
- **others**: Default.

```
best_parameters = grid_search.best_params_
clf = SVC(kernel='rbf', C= best_parameters['C'], gamma = best_parameters['gamma'], probability = True)
```

LR (Logistic Regression)

- **C**: Inverse of regularization strength was set to 10.
- **others**: Default.

```
clf = LogisticRegression(C=10)
```

KNN (K-Nearest Neighbors)

- **n_neighbors**: Number of neighbors to use by default for kneighbors queries was set to 10.
- **others**: Default.

```
clf = KNeighborsClassifier(n_neighbors = 3)
```
